# Supplementary material for: A dynamic knowledge graph approach to distributed self-driving laboratories
Source: Nat Commun. 2024 Jan 23;15:462. doi: 10.1038/s41467-023-44599-9 (PMC10805810; doi:10.1038/s41467-023-44599-9)
Supplement: Supplementary file 1 — Supplementary Information [file 41467_2023_44599_MOESM1_ESM.pdf]

Supplementary Information for
A Dynamic Knowledge Graph Approach to Distributed Self-Driving Laboratories

Jiaru Bai[1], Sebastian Mosbach[1,2], Connor J. Taylor[3,4,8], Dogancan Karan[2], Kok Foong Lee[5], Simon D. Rihm[1,2], Jethro Akroyd[1,2], Alexei A. Lapkin[1,2,4], Markus Kraft[1,2,6,7,*]

[*]Corresponding author: mk306@cam.ac.uk

[1]Department of Chemical Engineering and Biotechnology, University of Cambridge, Philippa Fawcett Drive, Cambridge CB3 0AS, United Kingdom

[2]CARES, Cambridge Centre for Advanced, Research and Education in Singapore, 1 Create Way, CREATE Tower, #05-05, 138602 Singapore

[3]Astex Pharmaceuticals, 436 Cambridge Science Park Milton Road, Cambridge CB4 0QA, United Kingdom

[4]Innovation Centre in Digital Molecular Technologies, Yusuf Hamied Department of Chemistry, University of Cambridge, Lensfield Road, Cambridge CB2 1EW, United Kingdom

[5]CMCL Innovations, Sheraton House, Cambridge CB3 0AX, United Kingdom

[6]School of Chemical and Biomedical Engineering, Nanyang Technological University, 62 Nanyang Drive, 637459 Singapore

[7]The Alan Turing Institute, London NW1 2DB, United Kingdom

[8]Present address: Faculty of Engineering, University of Nottingham, University Park, Nottingham, NG7 2RD, United Kingdom

Contents

A Supplementary Information **3**

A.1 Ontological representation . . . 3

A.1.1 Namespaces . . . 3

A.1.2 OntoCAPE . . . 4

A.1.3 OntoReaction . . . 11

A.1.4 OntoDoE . . . 17

A.1.5 OntoLab . . . 20

A.1.6 OntoVapourtec . . . 24

A.1.7 OntoHPLC . . . 29

A.1.8 OntoDerivation . . . 33

A.1.9 OntoGoal . . . 36

A.1.10 Object mapping and type validation . . . 39

A.1.11 Description logic representation . . . 39

A.2 Agent framework . . . 52

A.2.1 Derived information framework . . . 52

A.2.2 DoE Agent . . . 52

A.2.3 VapourtecSchedule Agent . . . 53

A.2.4 Vapourtec Agent . . . 55

A.2.5 HPLC Agent . . . 57

A.2.6 HPLCPostPro Agent . . . 57

A.2.7 ROGI Agent . . . 61

A.2.8 ROG Agent . . . 63

A.2.9 Distributed deployment . . . 67

A.2.10 Derived information stepping . . . 69

A.3 Experimental . . . 74

A.3.1 HPLC calibration for benzylideneactone . . . 74

A.3.2 Cost of chemicals . . . 76

A.3.3 Reproducibility across laboratories . . . 76

A.3.4 Self-optimisation campaign . . . 77

A Supplementary Information

A.1 Ontological representation

This section provides technical details of the ontology development involved in this work. We first list the namespaces of the ontologies and then present their schematic representations, domain competency questions and example instantiation. We also discuss object mapping and type validation when processing the data queried from the knowledge graph. Finally, we provide description logic representations of the ontologies developed in this work.

A.1.1 Namespaces

The ontological representation involves several namespaces, which are categorised into four sections as shown in Supplementary Triples S1. These sections include common namespaces developed for general purposes, namespaces from the OntoCAPE ontology, namespaces developed as part of the World Avatar project, and some dummy namespaces used for example instantiation.

Supplementary Triples S1: *Namespaces involved in ontological representation.*

```
## common namespaces
@prefix rdf: <http://www.w3.org/1999/02/22-rdf-syntax-ns#> .
@prefix rdfs: <http://www.w3.org/2000/01/rdf-schema#> .
@prefix skos: <http://www.w3.org/2004/02/skos/core#> .
@prefix xsd: <http://www.w3.org/2001/XMLSchema#> .
@prefix time: <http://www.w3.org/2006/time#> .
@prefix om: <http://www.ontology-of-units-of-measure.org/resource/om-2/> .
@prefix owl: <http://www.w3.org/2002/07/owl#> .
@prefix saref: <https://saref.etsi.org/core/> .
@prefix yago: <http://dbpedia.org/class/yago/> .
@prefix dbo: <https://dbpedia.org/ontology/> .
@prefix dbr: <https://dbpedia.org/resource/> .

## OntoCAPE namespaces
@prefix OntoCAPE_Behavior: <http://www.theworldavatar.com/ontology/ontocape/
    chemical_process_system/CPS_behavior/behavior.owl#> .
@prefix OntoCAPE_Material: <http://www.theworldavatar.com/ontology/ontocape/
    material/material.owl#> .
@prefix OntoCAPE_Phase_System: <http://www.theworldavatar.com/ontology/ontocape/
    material/phase_system/phase_system.owl#> .
@prefix OntoCAPE_Reaction_Mechanism: <http://www.theworldavatar.com/ontology/
    ontocape/material/substance/reaction_mechanism.owl#> .
@prefix OntoCAPE_Substance: <http://www.theworldavatar.com/ontology/ontocape/
    material/substance/substance.owl#> .
@prefix OntoCAPE_System: <http://www.theworldavatar.com/ontology/ontocape/
    upper_level/system.owl#> .

## the world avatar namespaces
@prefix OntoSpecies: <http://www.theworldavatar.com/ontology/ontospecies/
    OntoSpecies.owl#> .
@prefix OntoKin: <http://www.theworldavatar.com/ontology/ontokin/OntoKin.owl#> .

@prefix OntoReaction: <https://www.theworldavatar.com/kg/ontoreaction/> .
@prefix OntoDoE: <https://www.theworldavatar.com/kg/ontodoe/> .
```

```
@prefix OntoLab: <https://www.theworldavatar.com/kg/ontolab/> .
@prefix OntoVapourtec: <https://www.theworldavatar.com/kg/ontovapourtec/> .
@prefix OntoHPLC: <https://www.theworldavatar.com/kg/ontohplc/> .
@prefix OntoDerivation: <https://www.theworldavatar.com/kg/ontoderivation/> .
@prefix OntoGoal: <https://www.theworldavatar.com/kg/ontogoal/> .
@prefix OntoAgent: <http://www.theworldavatar.com/ontology/ontoagent/MSM.owl#> .


## dummy namespaces for example instantiation
@prefix _phase: <https://www.theworldavatar.com/kg/_phase/> .
@prefix _chem: <https://www.theworldavatar.com/kg/_chem/> .
@prefix _exp: <httpss://www.theworldavatar.com/kg/_exp/> .
@prefix _doe: <https://www.theworldavatar.com/kg/_doe/> .
@prefix _lab1: <https://www.theworldavatar.com/kg/_lab1/> .
@prefix _lab2: <https://www.theworldavatar.com/kg/_lab2/> .
@prefix _derivation: <https://www.theworldavatar.com/kg/_derivation/> .
@prefix _agent: <https://www.theworldavatar.com/kg/_agent/> .
@prefix _goal: <https://www.theworldavatar.com/kg/_goal/> .
```

A.1.2 OntoCAPE

Reuse of relevant expressions OntoCAPE [1] connects the chemical representation in chemical process engineering domain from three aspects: conceptual description as `ChemicalSpecies`, contextual phase properties as `Material`, and physical existence as `MaterialAmount`. Previously, the World Avatar has used concepts from OntoCAPE in the areas of kinetic modelling [2, 3] and dispersion simulation [4]. Supplementary Figure S1 illustrates relevant concepts, relationships, and instances of OntoCAPE [1] re-used in the World Avatar to describe chemicals presented in a laboratory environment. Specifically, three concepts, `OntoSpecies:Species`, `OntoReaction:Chemical` and `OntoLab:ChemicalAmount`, are made as either an equivalent class or sub-class of their OntoCAPE counterparts. The rationale for not directly using the concepts from OntoCAPE is to allow other customised definitions introduced in the following sections.

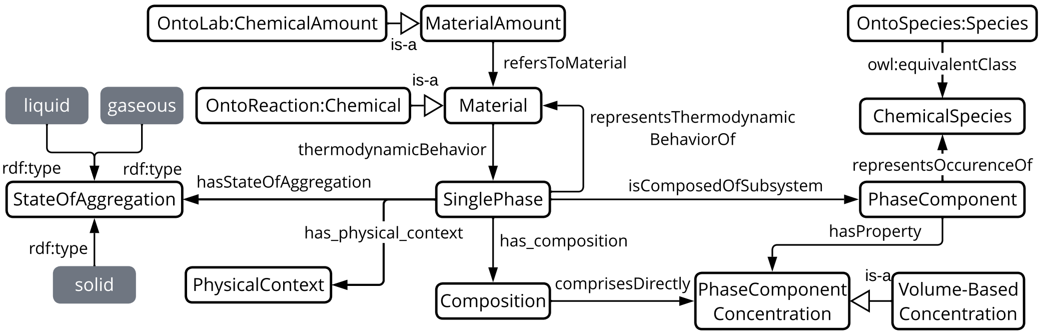

Supplementary Figure S1: *Ontological representation from OntoCAPE re-used for chemical representation in the World Avatar. For their corresponding namespaces please see Supplementary Table S1.*

Supplementary Table S1: *Short names and their corresponding namespaces in OntoCAPE.*

Short name	Corresponding namespace
MaterialAmount	OntoCAPE_Behavior
Material	OntoCAPE_Material
SinglePhase	OntoCAPE_Phase_System
PhysicalContext	OntoCAPE_Phase_System
StateOfAggregation	OntoCAPE_Phase_System
PhaseComponent	OntoCAPE_Phase_System
ChemicalSpecies	OntoCAPE_Substance
Composition	OntoCAPE_Phase_System
PhaseComponentConcentration	OntoCAPE_Phase_System
Volume-BasedConcentration	OntoCAPE_Phase_System
refersToMaterial	OntoCAPE_Behavior
thermodynamicBehavior	OntoCAPE_Material
representsThermodynamicBehaviorOf	OntoCAPE_Material
hasStateOfAggregation	OntoCAPE_Phase_System
has_physical_context	OntoCAPE_Phase_System
isComposedOfSubsystem	OntoCAPE_System
representsOccurenceOf	OntoCAPE_Phase_System
hasProperty	OntoCAPE_System
has_composition	OntoCAPE_Phase_System
comprisesDirectly	OntoCAPE_System
liquid	OntoCAPE_Phase_System
gaseous	OntoCAPE_Phase_System
solid	OntoCAPE_Phase_System

Example instantiation Supplementary Triples S2 describes phase concentrations derived from the starting materials and catalyst used by Jeraal et al. [5] but with a different internal standard. Specifically, naphthalene is replaced with biphenyl at a concentration of 0.06 M. In addition, the product concentration obtained during one of the experiments is presented. For unique identification, chemical species instantiated in OntoSpecies are employed throughout the phase representation. Minimum information on the species is provided to ensure that the example phase concentrations are self-contained.

Supplementary Triples S2: *Example instantiation for chemical representation using OntoCAPE and OntoSpecies.*

```
## chemical species instantiated using OntoSpecies for unique identification
# PubChem CID 240
<http://www.theworldavatar.com/kg/ontospecies/Species_54d8b46b-17bc-4bbd-a3cc-3
    b3a16d6ae4b>
   rdfs:label "C7H6O"^^xsd:string;
   skos:altLabel "benzaldehyde"^^xsd:string;
   OntoSpecies:hasCID <http://www.theworldavatar.com/kg/ontospecies/
       CID_1_Species_54d8b46b-17bc-4bbd-a3cc-3b3a16d6ae4b>;
.

<http://www.theworldavatar.com/kg/ontospecies/CID_1_Species_54d8b46b-17bc-4bbd-
```

```
    a3cc-3b3a16d6ae4b>
   rdf:type OntoSpecies:CID;
   OntoSpecies:value "240"^^xsd:string;
.

# PubChem CID 180
<http://www.theworldavatar.com/kg/ontospecies/Species_353d4667-e25d-476a-bd74-5
    c34723c8ea3>
   rdfs:label "C3H6O"^^xsd:string;
   skos:altLabel "acetone"^^xsd:string;
.

# PubChem CID 637759
<http://www.theworldavatar.com/kg/ontospecies/Species_f999de28-55dc-477e-8afc-
    e8802064e0d2>
   rdfs:label "C10H10O"^^xsd:string;
   skos:altLabel "benzalacetone"^^xsd:string;
.

# PubChem CID 640180
<http://www.theworldavatar.com/kg/ontospecies/Species_8765d201-0da9-4112-b653
    -3455002f535b>
   rdfs:label "C17H14O"^^xsd:string;
   skos:altLabel "dibenzylideneacetone"^^xsd:string;
.

# PubChem CID 14798
<http://www.theworldavatar.com/kg/ontospecies/Species_eab77458-560d-4ce9-9b5e
    -96650fc3e202>
   rdfs:label "HNaO"^^xsd:string;
   skos:altLabel "NaOH"^^xsd:string;
.

# PubChem CID 6342
<http://www.theworldavatar.com/kg/ontospecies/Species_0401f93b-b62d-488e-ba1f-7
    d5c37e365cb>
   rdfs:label "CH3CN"^^xsd:string;
   skos:altLabel "acetonitrile"^^xsd:string;
.

# PubChem CID 702
<http://www.theworldavatar.com/kg/ontospecies/Species_63fefc5a-d49d-4841-a946-2
    cdb5f356983>
   rdfs:label "C2H6O"^^xsd:string;
   skos:altLabel "ethanol"^^xsd:string;
.

# PubChem CID 931
<http://www.theworldavatar.com/kg/ontospecies/Species_4fa4fdea-ed3d-4b0a-aee5-1
    f4e97dd2340>
   rdfs:label "C10H8"^^xsd:string;
   skos:altLabel "naphthalene"^^xsd:string;
.

# PubChem CID 7095
<http://www.theworldavatar.com/kg/ontospecies/Species_471ce681-98f3-4245-be7d-3
    f38dfb6dd22>
   rdfs:label "C12H10"^^xsd:string;
   skos:altLabel "biphenyl"^^xsd:string;
```

```
.

## chemical phase instances - input chemicals
# phase for the solution of 0.5 M benzaldehyde in acetonitrile with 0.06 M
    biphenyl as the internal standard for further HPLC analysis
_phase:Phase_1
   rdf:type OntoCAPE_Phase_System:SinglePhase;
   OntoCAPE_Material:representsThermodynamicBehavior _exp:InputChemical_1;
   OntoCAPE_Phase_System:hasStateOfAggregation OntoCAPE_Phase_System:liquid;
   OntoCAPE_Phase_System:has_composition _phase:Composition_1;
   OntoCAPE_System:isComposedOfSubsystem _phase:PhaseComponent_1;
   OntoCAPE_System:isComposedOfSubsystem _phase:PhaseComponent_6;
   OntoCAPE_System:isComposedOfSubsystem _phase:PhaseComponent_IS_Input;
.

_phase:Composition_1
   rdf:type OntoCAPE_Phase_System:Composition;
   OntoCAPE_System:comprisesDirectly _phase:PhaseComponent_1_Property_1;
   OntoCAPE_System:comprisesDirectly _phase:PhaseComponent_6_Property_1;
   OntoCAPE_System:comprisesDirectly _phase:PhaseComponent_IS_Input_Property_1;
.

# phase for the solution of 6.73 M acetone in acetonitrile
_phase:Phase_2
   rdf:type OntoCAPE_Phase_System:SinglePhase;
   OntoCAPE_Material:representsThermodynamicBehavior _exp:InputChemical_2;
   OntoCAPE_Phase_System:hasStateOfAggregation OntoCAPE_Phase_System:liquid;
   OntoCAPE_Phase_System:has_composition _phase:Composition_2;
   OntoCAPE_System:isComposedOfSubsystem _phase:PhaseComponent_2;
   OntoCAPE_System:isComposedOfSubsystem _phase:PhaseComponent_6_2;
.

_phase:Composition_2
   rdf:type OntoCAPE_Phase_System:Composition;
   OntoCAPE_System:comprisesDirectly _phase:PhaseComponent_2_Property_1;
   OntoCAPE_System:comprisesDirectly _phase:PhaseComponent_6_Property_2;
.

# phase for the solution of 0.1 M NaOH in ethanol
_phase:Phase_3
   rdf:type OntoCAPE_Phase_System:SinglePhase;
   OntoCAPE_Material:representsThermodynamicBehavior _exp:InputChemical_3;
   OntoCAPE_Phase_System:hasStateOfAggregation OntoCAPE_Phase_System:liquid;
   OntoCAPE_Phase_System:has_composition _phase:Composition_3;
   OntoCAPE_System:isComposedOfSubsystem _phase:PhaseComponent_5;
   OntoCAPE_System:isComposedOfSubsystem _phase:PhaseComponent_7;
.

_phase:Composition_3
   rdf:type OntoCAPE_Phase_System:Composition;
   OntoCAPE_System:comprisesDirectly _phase:PhaseComponent_5_Property_1;
   OntoCAPE_System:comprisesDirectly _phase:PhaseComponent_7_Property_1;
.

# individual phase components and their concentration properties
_phase:PhaseComponent_1
   rdf:type OntoCAPE_Phase_System:PhaseComponent;
   OntoCAPE_Phase_System:representsOccurenceOf <http://www.theworldavatar.com/
       kg/ontospecies/Species_54d8b46b-17bc-4bbd-a3cc-3b3a16d6ae4b>;
```

```
    OntoCAPE_System:hasProperty _phase:PhaseComponent_1_Property_1;
.

_phase:PhaseComponent_2
    rdf:type OntoCAPE_Phase_System:PhaseComponent;
    OntoCAPE_Phase_System:representsOccurenceOf <http://www.theworldavatar.com/
        kg/ontospecies/Species_353d4667-e25d-476a-bd74-5c34723c8ea3>;
    OntoCAPE_System:hasProperty _phase:PhaseComponent_2_Property_1;
.

_phase:PhaseComponent_5
    rdf:type OntoCAPE_Phase_System:PhaseComponent;
    OntoCAPE_Phase_System:representsOccurenceOf <http://www.theworldavatar.com/
        kg/ontospecies/Species_eab77458-560d-4ce9-9b5e-96650fc3e202>;
    OntoCAPE_System:hasProperty _phase:PhaseComponent_5_Property_1;
.

_phase:PhaseComponent_6
    rdf:type OntoCAPE_Phase_System:PhaseComponent;
    OntoCAPE_Phase_System:representsOccurenceOf <http://www.theworldavatar.com/
        kg/ontospecies/Species_0401f93b-b62d-488e-ba1f-7d5c37e365cb>;
    OntoCAPE_System:hasProperty _phase:PhaseComponent_6_Property_1;
.

_phase:PhaseComponent_6_2
    rdf:type OntoCAPE_Phase_System:PhaseComponent;
    OntoCAPE_Phase_System:representsOccurenceOf <http://www.theworldavatar.com/
        kg/ontospecies/Species_0401f93b-b62d-488e-ba1f-7d5c37e365cb>;
    OntoCAPE_System:hasProperty _phase:PhaseComponent_6_Property_2;
.

_phase:PhaseComponent_7
    rdf:type OntoCAPE_Phase_System:PhaseComponent;
    OntoCAPE_Phase_System:representsOccurenceOf <http://www.theworldavatar.com/
        kg/ontospecies/Species_63fefc5a-d49d-4841-a946-2cdb5f356983>;
    OntoCAPE_System:hasProperty _phase:PhaseComponent_7_Property_1;
.

_phase:PhaseComponent_IS_Input
    rdf:type OntoCAPE_Phase_System:PhaseComponent;
    OntoCAPE_Phase_System:representsOccurenceOf <http://www.theworldavatar.com/
        kg/ontospecies/Species_471ce681-98f3-4245-be7d-3f38dfb6dd22>;
    OntoCAPE_System:hasProperty _phase:PhaseComponent_IS_Input_Property_1;
.

_phase:PhaseComponent_1_Property_1
    rdf:type OntoCAPE_Phase_System:Molarity;
    OntoCAPE_System:hasValue _phase:PhaseComponent_1_Property_1_ScalarValue_1;
.

_phase:PhaseComponent_1_Property_1_ScalarValue_1
    rdf:type OntoCAPE_System:ScalarValue;
    OntoCAPE_System:hasUnitOfMeasure om:molePerLitre;
    OntoCAPE_System:numericalValue "0.5"^^xsd:double;
.

_phase:PhaseComponent_2_Property_1
    rdf:type OntoCAPE_Phase_System:Molarity;
    OntoCAPE_System:hasValue _phase:PhaseComponent_2_Property_1_ScalarValue_1;
```

```
.

_phase:PhaseComponent_2_Property_1_ScalarValue_1
   rdf:type OntoCAPE_System:ScalarValue;
   OntoCAPE_System:hasUnitOfMeasure om:molePerLitre;
   OntoCAPE_System:numericalValue "6.73"^^xsd:double;
.

_phase:PhaseComponent_5_Property_1
   rdf:type OntoCAPE_Phase_System:Molarity;
   OntoCAPE_System:hasValue _phase:PhaseComponent_5_Property_1_ScalarValue_1;
.

_phase:PhaseComponent_5_Property_1_ScalarValue_1
   rdf:type OntoCAPE_System:ScalarValue;
   OntoCAPE_System:hasUnitOfMeasure om:molePerLitre;
   OntoCAPE_System:numericalValue "0.1"^^xsd:double;
.

_phase:PhaseComponent_6_Property_1
   rdf:type OntoCAPE_Phase_System:Molarity;
   OntoCAPE_System:hasValue _phase:PhaseComponent_6_Property_1_ScalarValue_1;
.

_phase:PhaseComponent_6_Property_1_ScalarValue_1
   rdf:type OntoCAPE_System:ScalarValue;
   OntoCAPE_System:hasUnitOfMeasure om:molePerLitre;
   OntoCAPE_System:numericalValue "18.1"^^xsd:double;
.

_phase:PhaseComponent_6_Property_2
   rdf:type OntoCAPE_Phase_System:Molarity;
   OntoCAPE_System:hasValue _phase:PhaseComponent_6_Property_2_ScalarValue_1;
.

_phase:PhaseComponent_6_Property_2_ScalarValue_1
   rdf:type OntoCAPE_System:ScalarValue;
   OntoCAPE_System:hasUnitOfMeasure om:molePerLitre;
   OntoCAPE_System:numericalValue "9.6"^^xsd:double;
.

_phase:PhaseComponent_7_Property_1
   rdf:type OntoCAPE_Phase_System:Molarity;
   OntoCAPE_System:hasValue _phase:PhaseComponent_7_Property_1_ScalarValue_1;
.

_phase:PhaseComponent_7_Property_1_ScalarValue_1
   rdf:type OntoCAPE_System:ScalarValue;
   OntoCAPE_System:hasUnitOfMeasure om:molePerLitre;
   OntoCAPE_System:numericalValue "17.1"^^xsd:double;
.

_phase:PhaseComponent_IS_Input_Property_1
   rdf:type OntoCAPE_Phase_System:Molarity;
   OntoCAPE_System:hasValue _phase:
       PhaseComponent_IS_Input_Property_1_ScalarValue_1;
.

_phase:PhaseComponent_IS_Input_Property_1_ScalarValue_1
```

```
    rdf:type OntoCAPE_System:ScalarValue;
    OntoCAPE_System:hasUnitOfMeasure om:molePerLitre;
    OntoCAPE_System:numericalValue "0.06"^^xsd:double;
.

## chemical phase instances - output chemical for one reaction experiment
_phase:Phase_OutputChemical_exp0
    rdf:type OntoCAPE_Phase_System:SinglePhase;
    OntoCAPE_Material:representsThermodynamicBehavior _exp:OutputChemical_4;
    OntoCAPE_Phase_System:hasStateOfAggregation OntoCAPE_Phase_System:liquid;
    OntoCAPE_Phase_System:has_composition _phase:Composition_OutputChemical_exp0;

    OntoCAPE_System:isComposedOfSubsystem _phase:PhaseComponent_3_exp0_oc;
    OntoCAPE_System:isComposedOfSubsystem _phase:PhaseComponent_IS_exp0_oc;
.

_phase:Composition_OutputChemical_exp0
    rdf:type OntoCAPE_Phase_System:Composition;
    OntoCAPE_System:comprisesDirectly _phase:PhaseComponent_3_exp0_oc_Property_1;

    OntoCAPE_System:comprisesDirectly _phase:
        PhaseComponent_InternalStandard_exp0_Property_1;
.

_phase:PhaseComponent_3_exp0_oc
    rdf:type OntoCAPE_Phase_System:PhaseComponent;
    OntoCAPE_Phase_System:representsOccurenceOf <http://www.theworldavatar.com/
        kg/ontospecies/Species_f999de28-55dc-477e-8afc-e8802064e0d2>;
    OntoCAPE_System:hasProperty _phase:PhaseComponent_3_exp0_oc_Property_1;
.

_phase:PhaseComponent_IS_exp0_oc
    rdf:type OntoCAPE_Phase_System:PhaseComponent;
    OntoCAPE_Phase_System:representsOccurenceOf <http://www.theworldavatar.com/
        kg/ontospecies/Species_471ce681-98f3-4245-be7d-3f38dfb6dd22>;
    OntoCAPE_System:hasProperty _phase:
        PhaseComponent_InternalStandard_exp0_Property_1;
.

_phase:PhaseComponent_3_exp0_oc_Property_1
    rdf:type OntoCAPE_Phase_System:Molarity;
    OntoCAPE_System:hasValue _phase:
        PhaseComponent_3_exp0_oc_Property_1_ScalarValue_1;
.

_phase:PhaseComponent_3_exp0_oc_Property_1_ScalarValue_1
    rdf:type OntoCAPE_System:ScalarValue;
    OntoCAPE_System:hasUnitOfMeasure om:molePerLitre;
    OntoCAPE_System:numericalValue "0.109"^^xsd:double;
.

_phase:PhaseComponent_InternalStandard_exp0_Property_1
    rdf:type OntoCAPE_Phase_System:Molarity;
    OntoCAPE_System:hasValue _phase:
        PhaseComponent_InternalStandard_exp0_Property_1_ScalarValue_1;
.

_phase:PhaseComponent_InternalStandard_exp0_Property_1_ScalarValue_1
    rdf:type OntoCAPE_System:ScalarValue;
```

```
    OntoCAPE_System:hasUnitOfMeasure om:molePerLitre;
    OntoCAPE_System:numericalValue "0.018"^^xsd:double;
.
```

A.1.3 OntoReaction

Key design considerations Supplementary Figure S2 illustrates OntoReaction (v1.10) ontology which is designed to represent the conceptual description of physical reaction experiments, including reaction scheme, reaction condition and performance indicator. Notably, this ontology is to be distinguished from OntoRXN [6], which is designed for reaction networks in computational chemistry applications and is covered by OntoKin [2] and OntoCompChem [7] in the World Avatar project.

OntoReaction emphasises data utilisation and sharing between different organisations, as well as formulating the generated data into an algorithmically accessible form. Therefore, the concepts and relationships are inspired by the schema of existing chemical reaction databases [8, 9] and reaction data mining studies [10, 11].

The key concept in OntoReaction is the `ReactionExperiment`, which represents a concrete occurrence of `ChemicalReaction`. Identifiers are added to facilitate reaction search and indexing, such as `ordID` and `hasRInChI`. Other available identifiers include `hasEquation`, `hasRDFILE`, `rxnSMILES`, `cdXML`, and `rxnCXSMILES`. Classification of reaction types can be made by linking the reaction instance to subclasses of `RXNO:MolecularProcess` [12].

To support flow chemistry applications, `Solvent` and `Catalyst` are added as subclasses of `OntoKin:Species`. This expands the World Avatar's coverage beyond gas-phase reactions. The `ReactionCondtion` and `PerformanceIndicator` are provided as generic concepts for extensions. As dimensional quantities, the extended concepts also inherit suitable subclasses of `om:Quantity` defined in the ontology of units of measure (OM) [13]. Additional reaction conditions and performance indicators relevant to other chemistry domains can be incorporated if necessary, such as those for photochemical and electrochemical reactions.

Domain competency questions

1. How many chemical identifiers can be used to represent a chemical reaction?
2. What are the possible reaction conditions that a reaction experiment can vary?
3. What are the available performance indicators that can be computed from a reaction experiment?
4. How many side products are expected for a given chemical reaction?
5. Which reaction condition is different between two given reaction experiments of the same chemical reaction?
6. At what temperature was the given reaction experiment conducted?
7. What is the yield of a given chemical reaction at a given reaction condition?
8. Which chemical species is a yield limiting species for a given reaction experiment?
9. If multiple measurements were sampled at a given reaction condition, what are the mean and variance of a particular performance indicator, *e.g.* reaction yield?

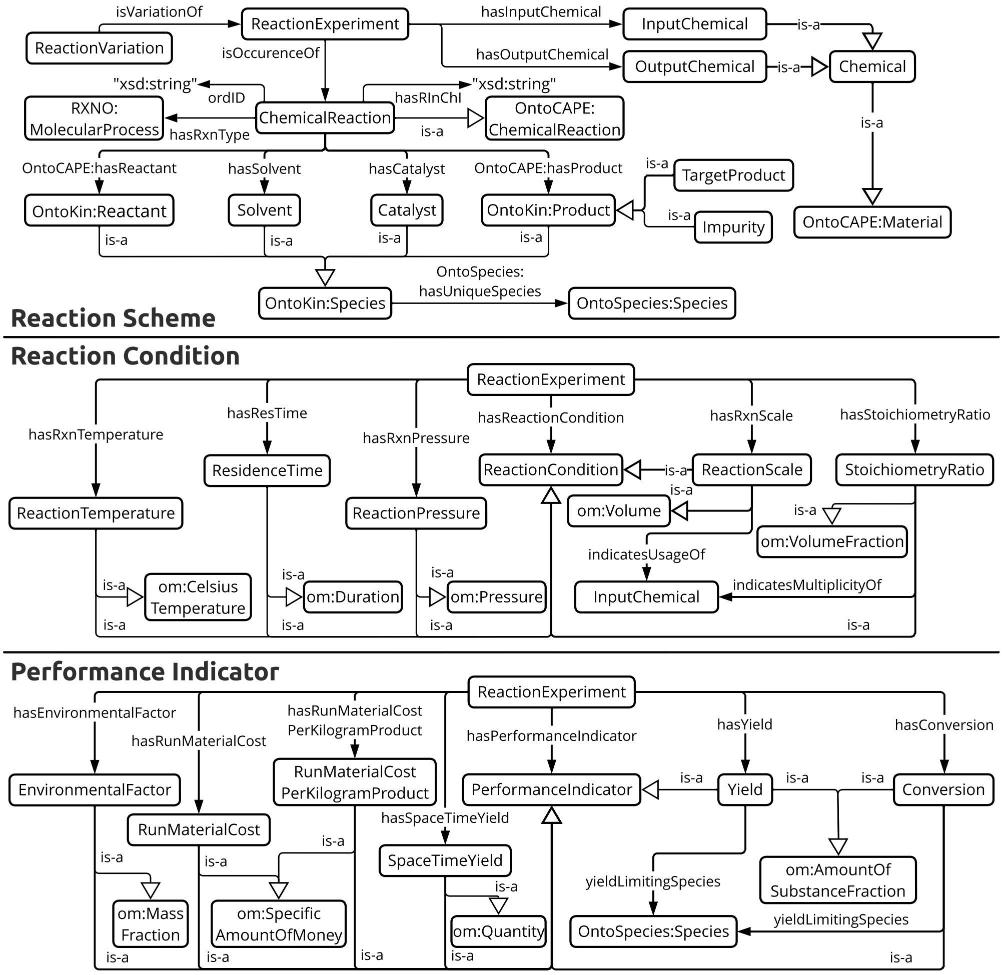

Supplementary Figure S2: *OntoReaction ontology for chemical reaction experiment. The relationship with hollow arrow "is-a" represents* `rdfs:subClassOf`*. The remaining concepts and relationships are under the OntoReaction namespace if not stated otherwise. The complete IRI for concept* `RXNO:MolecularProcess` *reads* `http://purl.obolibrary.org/obo/MOP_0000543`.

10. Which reaction condition is the most significant factor for a given performance indicator?
11. Which solvent is most commonly used for a given chemical reaction?
12. Which catalyst gives the best performance for a given chemical reaction?

Example instantiation The instantiation of the chemical reaction used in this study, along with one of its reaction experiments, are presented in Supplementary Triples S3. It is worth noting that the chemical species involved in the reaction are uniquely identified by the property `OntoSpecies:hasUniqueSpecies`. The input and output chemicals of the reaction experiment correspond to the phase concentrations described in Supplementary Triples S2. The experiment was conducted with a benzaldehyde to acetone to NaOH stoichiometry ratio of 1 : 22.5 : 0.12, a residence time of 10 minutes at a reactor temperature of 50 C, resulting in a run material cost of 330.58 £ L^{-1} and a reaction yield of 71.4%.

Supplementary Triples S3: *Example instantiation for chemical reaction experiment using OntoReaction.*

```
## chemical reaction
_chem:ChemRxn_1
   rdf:type OntoReaction:ChemicalReaction;
   OntoKin:hasEquation "C6H5CHO + CH3COCH3 = C10H10O"^^xsd:string;
   OntoCAPE_Reaction_Mechanism:hasReactant _chem:Species_1;
   OntoCAPE_Reaction_Mechanism:hasReactant _chem:Species_2;
   OntoCAPE_Reaction_Mechanism:hasProduct _chem:Species_3;
   OntoCAPE_Reaction_Mechanism:hasProduct _chem:Species_4;
   OntoReaction:hasCatalyst _chem:Species_5;
   OntoReaction:hasSolvent _chem:Species_6;
   OntoReaction:hasSolvent _chem:Species_7;
.

## species involved in the chemical reaction
_chem:Species_1
   rdf:type OntoKin:Reactant;
   OntoSpecies:hasUniqueSpecies <http://www.theworldavatar.com/kg/ontospecies/
       Species_54d8b46b-17bc-4bbd-a3cc-3b3a16d6ae4b>;
.

_chem:Species_2
   rdf:type OntoKin:Reactant;
   OntoSpecies:hasUniqueSpecies <http://www.theworldavatar.com/kg/ontospecies/
       Species_353d4667-e25d-476a-bd74-5c34723c8ea3>;
.

_chem:Species_3
   rdf:type OntoReaction:TargetProduct;
   OntoSpecies:hasUniqueSpecies <http://www.theworldavatar.com/kg/ontospecies/
       Species_f999de28-55dc-477e-8afc-e8802064e0d2>;
.

_chem:Species_4
   rdf:type OntoReaction:Impurity;
   OntoSpecies:hasUniqueSpecies <http://www.theworldavatar.com/kg/ontospecies/
       Species_8765d201-0da9-4112-b653-3455002f535b>;
.
```

```

_chem:Species_5
   rdf:type OntoReaction:Catalyst;
   OntoSpecies:hasUniqueSpecies <http://www.theworldavatar.com/kg/ontospecies/
       Species_eab77458-560d-4ce9-9b5e-96650fc3e202>;
.

_chem:Species_6
   rdf:type OntoReaction:Solvent;
   OntoSpecies:hasUniqueSpecies <http://www.theworldavatar.com/kg/ontospecies/
       Species_0401f93b-b62d-488e-ba1f-7d5c37e365cb>;
.

_chem:Species_7
   rdf:type OntoReaction:Solvent;
   OntoSpecies:hasUniqueSpecies <http://www.theworldavatar.com/kg/ontospecies/
       Species_63fefc5a-d49d-4841-a946-2cdb5f356983>;
.

## reaction experiment 0
_exp:ReactionExperiment_0
   rdf:type OntoReaction:ReactionExperiment;
   # relation to chemical reaction
   OntoReaction:isOccurenceOf _chem:ChemRxn_1;
   # input chemical
   OntoReaction:hasInputChemical _exp:InputChemical_1;
   OntoReaction:hasInputChemical _exp:InputChemical_2;
   OntoReaction:hasInputChemical _exp:InputChemical_3;
   # reaction conditions
   OntoReaction:hasResTime _exp:ResidenceTime_1;
   OntoReaction:hasRxnTemperature _exp:RxnTemperature_1;
   OntoReaction:hasStoichiometryRatio _exp:StoiRatio_1;
   OntoReaction:hasStoichiometryRatio _exp:StoiRatio_2;
   OntoReaction:hasStoichiometryRatio _exp:StoiRatio_3;
   OntoReaction:hasRxnScale _exp:RxnScale_1;
   # output chemical
   OntoReaction:hasOutputChemical _exp:OutputChemical_4;
   # performance indicator
   OntoReaction:hasYield _exp:Yield_1;
   OntoReaction:hasRunMaterialCost _exp:RunMaterialCost_1;
.

## input chemical
_exp:InputChemical_1
   rdf:type OntoReaction:InputChemical;
   OntoCAPE_Material:thermodynamicBehavior _phase:Phase_1;
.

_exp:InputChemical_2
   rdf:type OntoReaction:InputChemical;
   OntoCAPE_Material:thermodynamicBehavior _phase:Phase_2;
.

_exp:InputChemical_3
   rdf:type OntoReaction:InputChemical;
   OntoCAPE_Material:thermodynamicBehavior _phase:Phase_3;
.

## reaction conditions
```

```
_exp:StoiRatio_1
   rdf:type OntoReaction:StoichiometryRatio;
   OntoDoE:positionalID "http://www.theworldavatar.com/kg/ontospecies/
       Species_54d8b46b-17bc-4bbd-a3cc-3b3a16d6ae4b"^^xsd:string;
   om:hasPhenomenon _exp:ReactionExperiment_0;
   OntoReaction:indicatesMultiplicityOf _exp:InputChemical_1;
   om:hasValue _exp:StoiRatio_1_Measure_1;
.

_exp:StoiRatio_1_Measure_1
   rdf:type om:Measure;
   om:hasUnit om:one;
   om:hasNumericalValue "1.0"^^xsd:double;
.

_exp:StoiRatio_2
   rdf:type OntoReaction:StoichiometryRatio;
   OntoDoE:positionalID "http://www.theworldavatar.com/kg/ontospecies/
       Species_353d4667-e25d-476a-bd74-5c34723c8ea3"^^xsd:string;
   om:hasPhenomenon _exp:ReactionExperiment_0;
   OntoReaction:indicatesMultiplicityOf _exp:InputChemical_2;
   om:hasValue _exp:StoiRatio_2_Measure_1;
.

_exp:StoiRatio_2_Measure_1
   rdf:type om:Measure;
   om:hasUnit om:one;
   om:hasNumericalValue "22.5"^^xsd:double;
.

_exp:StoiRatio_3
   rdf:type OntoReaction:StoichiometryRatio;
   OntoDoE:positionalID "http://www.theworldavatar.com/kg/ontospecies/
       Species_eab77458-560d-4ce9-9b5e-96650fc3e202"^^xsd:string;
   om:hasPhenomenon _exp:ReactionExperiment_0;
   OntoReaction:indicatesMultiplicityOf _exp:InputChemical_3;
   om:hasValue _exp:StoiRatio_3_Measure_1;
.

_exp:StoiRatio_3_Measure_1
   rdf:type om:Measure;
   om:hasUnit om:one;
   om:hasNumericalValue "0.12"^^xsd:double;
.

_exp:RxnScale_1
   rdf:type OntoReaction:ReactionScale;
   OntoDoE:positionalID "http://www.theworldavatar.com/kg/ontospecies/
       Species_54d8b46b-17bc-4bbd-a3cc-3b3a16d6ae4b"^^xsd:string;
   om:hasPhenomenon _exp:ReactionExperiment_0;
   OntoReaction:indicatesUsageOf _exp:InputChemical_1;
   om:hasValue _exp:RxnScale_1_Measure_1;
.

_exp:RxnScale_1_Measure_1
   rdf:type om:Measure;
   om:hasUnit om:millilitre;
   om:hasNumericalValue "2"^^xsd:double;
.
```

```
_exp:ResidenceTime_1
   rdf:type OntoReaction:ResidenceTime;
   om:hasPhenomenon _exp:ReactionExperiment_0;
   om:hasValue _exp:ResidenceTime_1_Measure_1;
.

_exp:ResidenceTime_1_Measure_1
   rdf:type om:Measure;
   om:hasUnit om:minute-Time;
   om:hasNumericalValue "10"^^xsd:double;
.

_exp:RxnTemperature_1
   rdf:type OntoReaction:ReactionTemperature;
   om:hasPhenomenon _exp:ReactionExperiment_0;
   om:hasValue _exp:RxnTemperature_1_Measure_1;
.

_exp:RxnTemperature_1_Measure_1
   rdf:type om:Measure;
   om:hasUnit om:degreeCelsius;
   om:hasNumericalValue "50"^^xsd:double;
.

## output chemical
_exp:OutputChemical_4
   rdf:type OntoReaction:OutputChemical;
   OntoCAPE_Material:thermodynamicBehavior _phase:Phase_OutputChemical_exp0;
.

## performance indicators
_exp:Yield_1
   rdf:type OntoReaction:Yield;
   om:hasPhenomenon _exp:ReactionExperiment_0;
   om:hasValue _exp:Yield_1_Measure_1;
.

_exp:Yield_1_Measure_1
   rdf:type om:Measure;
   om:hasUnit om:percent;
   om:hasNumericalValue "71.4"^^xsd:double;
.

_exp:RunMaterialCost_1
   rdf:type OntoReaction:RunMaterialCost;
   om:hasPhenomenon _exp:ReactionExperiment_0;
   om:hasValue _exp:RunMaterialCost_1_Measure_1;
.

_exp:RunMaterialCost_1_Measure_1
   rdf:type om:Measure;
   om:hasUnit om:poundSterlingPerLitre;
   om:hasNumericalValue "330.58"^^xsd:double;
.
```

A.1.4 OntoDoE

Key design considerations OntoDoE (v1.11) is an ontological markup designed for the conceptualisation of design of experiments (DoE) studies. In contrast to EXPO ontology [14], which provides a comprehensive description of scientific experiments, OntoDoE provides a suitable description to capture the metadata of DoE conducted by software packages. In this regard, ODE [15] is a relevant ontology developed for the numerical DoE, but it is not publicly available and thus direct reuse is not possible. Hence, we create our own OntoDoE.

Supplementary Figure S3 maps the core concepts and relationships of OntoDoE, which follows the abstraction adopted by Garud et al. [16]. To construct a DoE study, a domain needs to be defined. It can comprise both design variables, either continuous or categorical, and fixed parameters. The primary objective of a DoE study is to suggest a new set of conditions in the search space that optimises the desired system responses based on available historical data. This process may involve different sampling strategies, which are highly relevant to the chosen modelling tools. In this work, we employ the Python package `summit` [17], and thus OntoDoE also reflects its available algorithms and the naming conventions of its data classes.

OntoDoE is intended to be used in conjunction with OntoReaction. Specifically, instances of `OntoReaction:ReactionExperiment` are used to represent both historical or new experiments. If no prior data is available, OntoDoE can still be used by providing a DoE instance as a template for the `OntoReaction:ChemicalReaction` to be optimised.

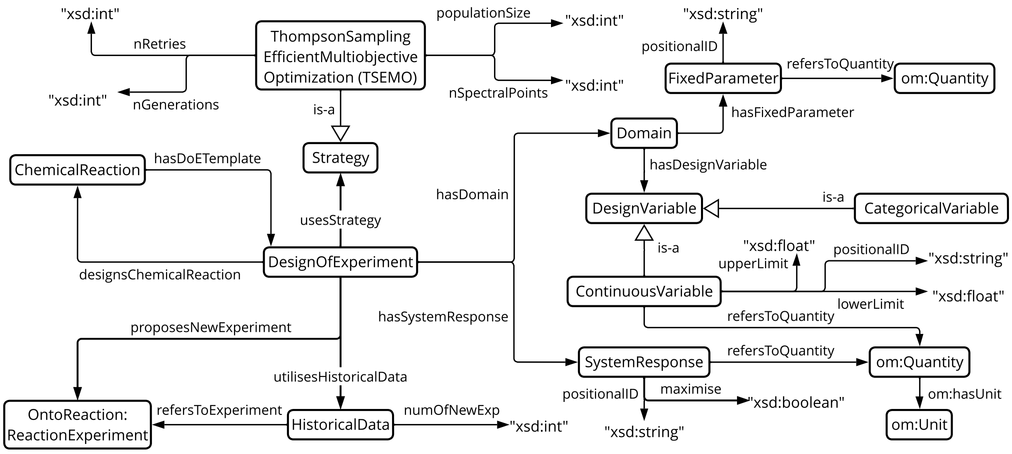

Supplementary Figure S3: *OntoDoE ontology for the design of experiments. The relationship with hollow arrow "is-a" represents* `rdfs:subClassOf`*. The remaining concepts and relationships are under the OntoDoE namespace if not stated otherwise.*

Domain competency questions

1. What types of strategies exist?
2. What are the key hyperparameters that can be configured for a given strategy?
3. What types of variables can be contained in a domain?

4. Is a given reaction experiment utilised by any DoE study?
5. How was a given reaction experiment suggested from the DoE study?
6. What strategy was used to suggest a given reaction experiment?
7. What chemical reaction is a given DoE study designing?
8. What are the objectives (system responses) of a given DoE study?
9. What are the design variables of a given DoE domain?
10. Is there any fixed parameter of a given DoE domain?
11. What is the most common DoE strategy used when optimising a given chemical reaction?
12. Which DoE strategy performs best for a given chemical reaction?

Example instantiation Supplementary Triples S4 lists the machine-readable version of a DoE study instantiated using OntoDoE. It designs the chemical reaction and utilises the experiment presented in Supplementary Triples S3 as historical data. The aim is to propose a new reaction experiment to be executed in the next iteration. The TSEMO algorithm is employed as a strategy with its default settings for this purpose. The study domain comprises four continuous variables, including the stoichiometric ratio of reactant acetone and the catalyst NaOH, as well as the residence time and reaction temperature. Additionally, one parameter is fixed, which is the stoichiometric ratio of the primary starting material, benzaldehyde. The stoichiometric ratio of each inlet chemical stream is uniquely identified by the `OntoSpecies:Species` IRI of its main solute. The objective is to maximise the reaction yield while minimising the cost of the run material.

Supplementary Triples S4: *Example instantiation for design of experiments using OntoDoE.*

```
## DoE instance
_doe:DesignOfExperiment_1
   rdf:type OntoDoE:DesignOfExperiment;
   OntoDoE:designsChemicalReaction _chem:ChemRxn_1;
   OntoDoE:usesStrategy _doe:Strategy_1;
   OntoDoE:hasDomain _doe:Domain_1;
   OntoDoE:hasSystemResponse _doe:SystemResponse_1;
   OntoDoE:hasSystemResponse _doe:SystemResponse_2;
   OntoDoE:utilisesHistoricalData _doe:HistoricalData_1;
.

## strategy
_doe:Strategy_1
   rdf:type OntoDoE:TSEMO;
   OntoDoE:nRetries "10"^^xsd:integer;
   OntoDoE:nSpectralPoints "1500"^^xsd:integer;
   OntoDoE:nGenerations "1000"^^xsd:integer;
   OntoDoE:populationSize "100"^^xsd:integer;
.

## domain
_doe:Domain_1
   rdf:type OntoDoE:Domain;
   OntoDoE:hasDesignVariable _doe:ContinuousVariable_1;
```

```
    OntoDoE:hasDesignVariable _doe:ContinuousVariable_2;
    OntoDoE:hasDesignVariable _doe:ContinuousVariable_3;
    OntoDoE:hasDesignVariable _doe:ContinuousVariable_4;
    OntoDoE:hasFixedParameter _doe:FixedParameter_1;
.

## continuous variables
_doe:ContinuousVariable_1
    rdf:type OntoDoE:ContinuousVariable;
    OntoDoE:refersToQuantity _doe:StoiRatio;
    OntoDoE:positionalID "http://www.theworldavatar.com/kg/ontospecies/
        Species_353d4667-e25d-476a-bd74-5c34723c8ea3"^^xsd:string;
    OntoDoE:upperLimit "40"^^xsd:double;
    OntoDoE:lowerLimit "5"^^xsd:double;
.

_doe:ContinuousVariable_2
    rdf:type OntoDoE:ContinuousVariable;
    OntoDoE:refersToQuantity _doe:StoiRatio;
    OntoDoE:positionalID "http://www.theworldavatar.com/kg/ontospecies/
        Species_eab77458-560d-4ce9-9b5e-96650fc3e202"^^xsd:string;
    OntoDoE:upperLimit "0.2"^^xsd:double;
    OntoDoE:lowerLimit "0.05"^^xsd:double;
.

_doe:StoiRatio
    rdf:type OntoReaction:StoichiometryRatio;
    om:hasUnit om:one;
.

_doe:ContinuousVariable_3
    rdf:type OntoDoE:ContinuousVariable;
    OntoDoE:refersToQuantity _doe:ResTime;
    OntoDoE:upperLimit "15"^^xsd:double;
    OntoDoE:lowerLimit "5"^^xsd:double;
.

_doe:ResTime
    rdf:type OntoReaction:ResidenceTime;
    om:hasUnit om:minute-Time;
.

_doe:ContinuousVariable_4
    rdf:type OntoDoE:ContinuousVariable;
    OntoDoE:refersToQuantity _doe:RxnTemp;
    OntoDoE:upperLimit "70"^^xsd:double;
    OntoDoE:lowerLimit "30"^^xsd:double;
.

_doe:RxnTemp
    rdf:type OntoReaction:ReactionTemperature;
    om:hasUnit om:degreeCelsius;
.

## fixed parameters
_doe:FixedParameter_1
    rdf:type OntoDoE:FixedParameter;
    OntoDoE:refersToQuantity _doe:StoiRatio_1;
    OntoDoE:positionalID "http://www.theworldavatar.com/kg/ontospecies/
```

```
        Species_54d8b46b-17bc-4bbd-a3cc-3b3a16d6ae4b"^^xsd:string;
.

_doe:StoiRatio_1
   rdf:type OntoReaction:StoichiometryRatio;
   om:hasValue _doe:StoiRatio_1_Measure_1;
.

_doe:StoiRatio_1_Measure_1
   rdf:type om:Measure;
   om:hasUnit om:one;
   om:hasNumericalValue "1.0"^^xsd:double;
.

## system responses
_doe:SystemResponse_1
   rdf:type OntoDoE:SystemResponse;
   OntoDoE:refersToQuantity OntoReaction:Yield;
   OntoDoE:maximise "true"^^xsd:boolean;
.

_doe:SystemResponse_2
   rdf:type OntoDoE:SystemResponse;
   OntoDoE:refersToQuantity OntoReaction:RunMaterialCost;
   OntoDoE:maximise "false"^^xsd:boolean;
.

## historical data
_doe:HistoricalData_1
   rdf:type OntoDoE:HistoricalData;
   OntoDoE:refersToExperiment _exp:ReactionExperiment_0;
   OntoDoE:numOfNewExp "1"^^xsd:integer;
.
```

A.1.5 OntoLab

Key design considerations OntoLab (v1.9) is an ontological markup for the digital twin of laboratories. The current iteration focuses on the functional aspects of the laboratories, including laboratory equipment and chemical containers as illustrated in Supplementary Fig. S4. The geospatial and visualisation aspects of laboratories are omitted for simplification. To the best of our knowledge, there is no single ontology for laboratory environments that is readily available and fits our purpose. Therefore, concepts from different ontologies are utilised wherever suitable.

We describe a piece of `LabEquipment` from three perspectives, including its static specifications, the dynamic configuration for actuation, and the measurements that can be acquired from its sensors. The inspiration comes from the process of abstracting relevant information on the hardware involved in Jeraal et al. [5]. The specifications are normally dimensional quantities, *e.g.* height, width, and price, which we use concepts from OM. The other two perspectives align with the Smart Applications REFerence (SAREF) [18] ontology developed for the Internet of Things (IoT). We thus define the `LabEquipment` as a subclass of `saref:Device`, which allows inheriting other useful concepts and relationships provided in the SAREF ontology. The dynamic configuration part connects the abstract-level data

expressed in OntoReaction with concrete equipment realisation by translating the reaction conditions to `ParameterSetting` and further assembling `EquipmentSettings` for corresponding lab equipment. The measurement obtained from the lab equipment varies across kits that serve different purposes. In practice, both configuration and data collection are done by the software agent that manages each piece of equipment. For those designs please refer to later sections.

A few relevant concepts exist in OntoCAPE for describing containers, however, they are defined at the level of a chemical plant and also not generic enough to cover the different types of containers in a lab environment. We thus create `ChemicalContainer` class. Following a similar naming convention, `ChemicalAmount` is proposed to represent the physical existence of chemicals in the container. Different quantitative amounts can be attached to track the consumption and refill of the chemicals.

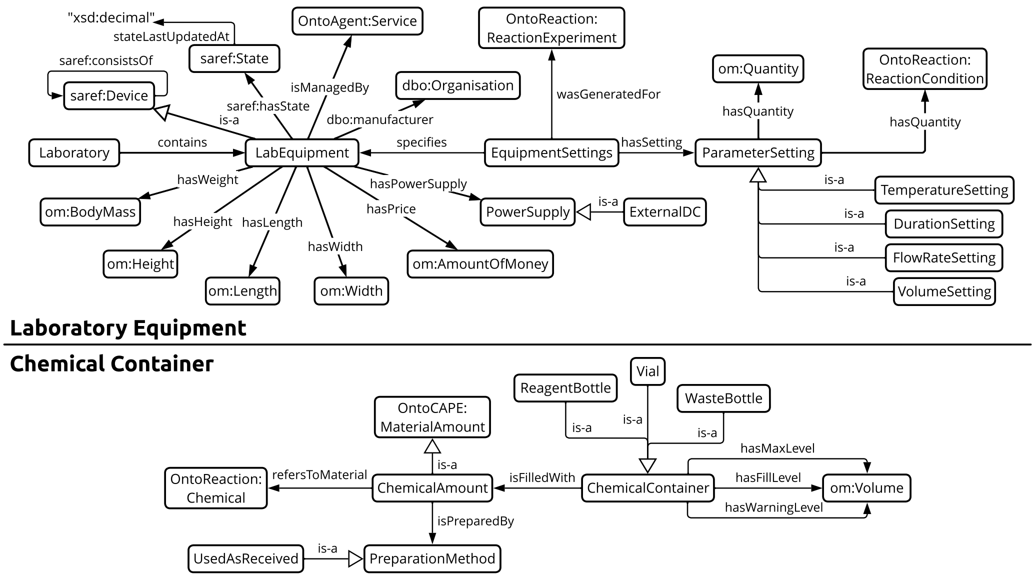

Supplementary Figure S4: *OntoLab ontology for laboratory digital twin. The relationship with hollow arrow "is-a" represents `rdfs:subClassOf`. The remaining concepts and relationships are under the OntoLab namespace if not stated otherwise.*

Domain competency questions

1. What are the possible equipment settings for a given lab equipment?
2. Who is the manufacturer of a given lab equipment?
3. Are there any sub-parts of a given lab equipment?
4. What is the power supply mode of a given lab equipment?
5. How many preparation methods exist?
6. How many chemical containers exist in a given laboratory?
7. How many bottles are available for a given chemical in a given laboratory?

8. Does a chemical container contain a given chemical?
9. What is the maximum filling level of a given chemical container?
10. What is the current filling level of a given chemical container?
11. Does a given chemical container need a refill?
12. How was the chemical in a given chemical container prepared?

Example instantiation Supplementary Triples S5 lists a snippet of statements for the two labs in Cambridge and Singapore. For simplification, only the existence of lab equipment is included in the example. A vial and a reagent bottle are also instantiated as examples of chemical containers.

Supplementary Triples S5: *Example instantiation for laboratories in Cambridge and Singapore using OntoLab.*

```
## Cambridge lab
# existence of lab equipment
_lab1:Lab_1
   rdf:type OntoLab:Laboratory;
   # Vapourtec flow chemistry platform
   OntoLab:contains _lab1:VapourtecRS400;
   OntoLab:contains _lab1:VapourtecR4;
   OntoLab:contains _lab1:VapourtecR2_1;
   OntoLab:contains _lab1:VapourtecR2_2;
   OntoLab:contains _lab1:VapourtecR2_3;
   OntoLab:contains _lab1:VapourtecR2_4;
   OntoLab:contains _lab1:AutoSampler;
   # HPLC
   OntoLab:contains _lab1:Shimadzu_HPLC;
.

# chemical containers - vial
_lab1:Vial_1
   rdf:type OntoLab:Vial;
   OntoLab:isFilledWith _lab1:ChemicalAmount_1;
   OntoLab:hasMaxLevel _lab1:Vial_1_level_max;
   OntoLab:hasWarningLevel _lab1:Vial_1_level_warning;
   OntoLab:hasFillLevel _lab1:Vial_1_level_fill;
.

_lab1:ChemicalAmount_1
   rdf:type OntoLab:ChemicalAmount;
   OntoCAPE_Behavior:refersToMaterial _lab1:Chemical_1;
   OntoLab:containsUnidentifiedComponent "false"^^xsd:boolean;
.

_lab1:Chemical_1
   rdf:type OntoReaction:Chemical;
   OntoCAPE_Material:thermodynamicBehavior _phase:Phase_1;
.

_lab1:Vial_1_level_max
   rdf:type om:Volume;
   om:hasValue _lab1:Vial_1_level_max_value;
.

```

```
_lab1:Vial_1_level_max_value
   rdf:type om:Measure;
   om:hasUnit om:millilitre;
   om:hasNumericalValue "35"^^xsd:double;
.

_lab1:Vial_1_level_warning
  rdf:type om:Volume;
  om:hasValue _lab1:Vial_1_level_warning_value;
.

_lab1:Vial_1_level_warning_value
  rdf:type om:Measure;
  om:hasUnit om:millilitre;
  om:hasNumericalValue "5"^^xsd:double;
.

_lab1:Vial_1_level_fill
   rdf:type om:Volume;
   om:hasValue _lab1:Vial_1_level_fill_value;
.

_lab1:Vial_1_level_fill_value
   rdf:type om:Measure;
   om:hasUnit om:millilitre;
   om:hasNumericalValue "35"^^xsd:double;
.

## Singapore lab
# existence of lab equipment
_lab2:Lab_2
   rdf:type OntoLab:Laboratory;
   # Vapourtec flow chemistry platform
   OntoLab:contains _lab2:VapourtecRS400;
   OntoLab:contains _lab2:VapourtecR4;
   OntoLab:contains _lab2:VapourtecR2_1;
   OntoLab:contains _lab2:VapourtecR2_2;
   OntoLab:contains _lab2:VapourtecR2_3;
   OntoLab:contains _lab2:VapourtecR2_4;
   # HPLC
   OntoLab:contains _lab2:Agilent_HPLC;
.

# chemical containers - reagent bottle
_lab2:ReagentBottle_1
   rdf:type OntoLab:ReagentBottle;
   OntoLab:isFilledWith _lab2:ChemicalAmount_1;
   OntoLab:hasMaxLevel _lab2:ReagentBottle_1_level_max;
   OntoLab:hasWarningLevel _lab2:ReagentBottle_1_level_warning;
   OntoLab:hasFillLevel _lab2:ReagentBottle_1_level_fill;
.

_lab2:ReagentBottle_1_level_fill
   rdf:type om:Volume;
   om:hasValue _lab2:ReagentBottle_1_level_fill_Value;
.

_lab2:ReagentBottle_1_level_fill_Value
   rdf:type om:Measure;
```

```
    om:hasUnit om:millilitre;
    om:hasNumericalValue "200"^^xsd:double;
.

# for simplification, _lab2:ReagentBottle_1_level_max and _lab2:
    ReagentBottle_1_level_warning are omitted here
```

A.1.6 OntoVapourtec

Key design considerations OntoVapourtec (v1.9) is an ontological markup developed for the Vapourtec flow chemistry system employed in this case study. The core concepts are the individual equipment that extends `OntoLab:LabEquipment`, *i.e.* the integrated system `VapourtecRS400` that consists of tube reactor, pumps, and optionally an autosampler depending on how the chemicals are sourced. The configuration of the equipment relies on the available application programming interfaces (APIs). Each parameter will be translated to construct the final experiment file for execution. More details are covered in Supplementary Section A.2.4.

Domain competency questions

1. What are the possible states of the Vapourtec flow chemistry system?
2. What parameters can I set to configure the Vapourtec system?
3. What is the current state of a given Vapourtec reactor?
4. What is the set point for the flow rate of a given Vapourtec pump?
5. How many sites do a given autosampler have?
6. What is the internal volume of a given Vapourtec reactor?
7. Is the material of a given Vapourtec reactor compatible with a given reactant?
8. What is the operation limit of a given Vapourtec reactor?
9. Is this Vapourtec reactor able to perform a given reaction experiment?
10. Where is a given Vapourtec reactor located?
11. Which Vapourtec reactor was used for a given reaction experiment?
12. Is a given Vapourtec setup able to provide input chemicals required for a given reaction experiment?

Example instantiation The example instantiation of two Vapourtec setups located in the Cambridge and Singapore labs is presented in Supplementary Triples S6. For simplicity, we consider a reactor with a volume of 10 mL and an operating temperature range of 20 to 150 C. In the example, the pump in Cambridge sources chemicals from an autosampler site that can be dynamically configured, while the pump in Singapore pumps from a reagent bottle.

Supplementary Triples S6: *Example instantiation for flow chemistry system in both Cambridge and Singapore lab using OntoVapourtec.*

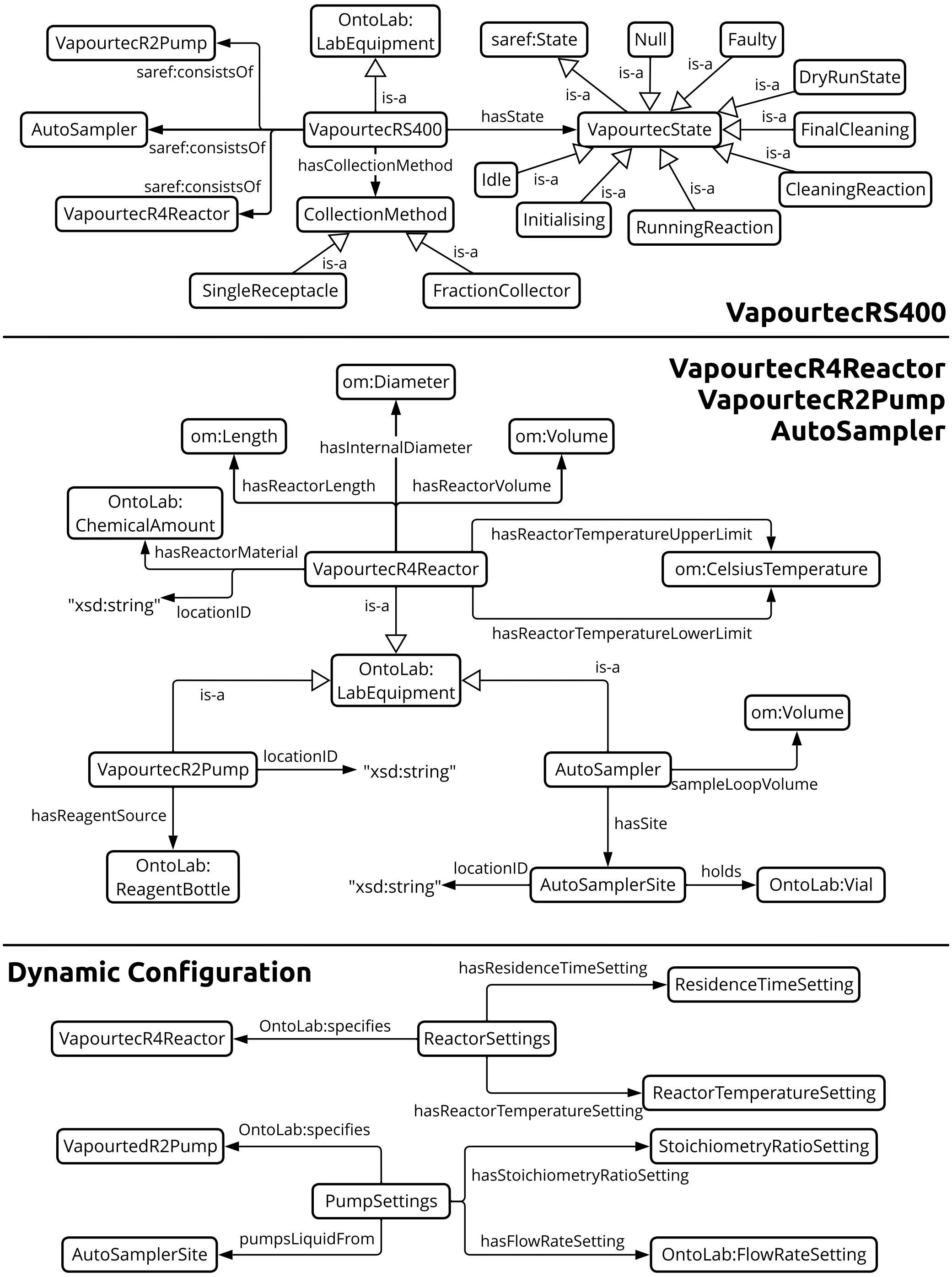

Supplementary Figure S5: *Core concepts and relationships in OntoVapourtec ontology for Vapourtec flow chemistry platform. The relationship with hollow arrow "is-a" represents* `rdfs:subClassOf`*. The remaining concepts and relationships are under the OntoVapourtec namespace if not stated otherwise.*

```
## Cambridge lab - Vapourtec
# vapourtec rs400
_lab1:VapourtecRS400
   rdf:type OntoVapourtec:VapourtecRS400;
   # state
   saref:hasState _lab1:VapourtecRS400_state;
   # equipment parts
   saref:consistsOf _lab1:VapourtecR4;
   saref:consistsOf _lab1:VapourtecR2_1;
   saref:consistsOf _lab1:VapourtecR2_2;
   saref:consistsOf _lab1:VapourtecR2_3;
   saref:consistsOf _lab1:VapourtecR2_4;
   saref:consistsOf _lab1:AutoSampler;
   # managing agent
   OntoLab:isManagedBy _agent:VapourtecAgent_Lab1;
.

# state
_lab1:VapourtecRS400_state
   rdf:type OntoVapourtec:Idle;
   OntoLab:stateLastUpdatedAt 1678887921;
.

# vapourtec r4 reactor
_lab1:VapourtecR4
   rdf:type OntoVapourtec:VapourtecR4Reactor;
   OntoVapourtec:locationID "4"^^xsd:string;
   OntoVapourtec:hasReactorVolume _lab1:VapourtecR4_ReactorVolume;
   OntoVapourtec:hasReactorTemperatureLowerLimit _lab1:
       VapourtecR4_ReactorTemperatureLower;
   OntoVapourtec:hasReactorTemperatureUpperLimit _lab1:
       VapourtecR4_ReactorTemperatureUpper;
.

_lab1:VapourtecR4_ReactorVolume
   rdf:type om:Volume;
   om:hasValue _lab1:VapR4_ReactorVolume_Value;
.

_lab1:VapR4_ReactorVolume_Value
   rdf:type om:Measure;
   om:hasUnit om:millilitre;
   om:hasNumericalValue "10"^^xsd:double;
.

_lab1:VapourtecR4_ReactorTemperatureLower
   rdf:type om:CelsiusTemperature;
   om:hasValue _lab1:VapR4_ReactorTempLower_Value;
.

_lab1:VapR4_ReactorTempLower_Value
   rdf:type om:Measure;
   om:hasUnit om:degreeCelsius;
   om:hasNumericalValue "20"^^xsd:double;
.

_lab1:VapourtecR4_Dummy_ReactorTemperatureUpper
   rdf:type om:CelsiusTemperature;
   om:hasValue _lab1:VapR4_ReactorTempUpper_Value;
```

```
.

_lab1:VapR4_ReactorTempUpper_Value
   rdf:type om:Measure;
   om:hasUnit om:degreeCelsius;
   om:hasNumericalValue "150"^^xsd:double;
.

# vapourtec r2 pump - only present one here as an example
_lab1:VapourtecR2_1
   rdf:type OntoVapourtec:VapourtecR2Pump;
   OntoVapourtec:locationID "A"^^xsd:string;
   # this pump is not connected to any ReagentBottle, so it will be considered
       as sourcing from the autosampler
.

## Singapore lab - Vapourtec
# vapourtec rs400
_lab2:VapourtecRS400
   rdf:type OntoVapourtec:VapourtecRS400;
   # state
   saref:hasState _lab2:VapourtecRS400_state;
   # equipment parts
   saref:consistsOf _lab2:VapourtecR4;
   saref:consistsOf _lab2:VapourtecR2_1;
   saref:consistsOf _lab2:VapourtecR2_2;
   saref:consistsOf _lab2:VapourtecR2_3;
   saref:consistsOf _lab2:VapourtecR2_4;
   # managing agent
   OntoLab:isManagedBy _agent:VapourtecAgent_Lab2;
.

# state
_lab2:VapourtecRS400_state
   rdf:type OntoVapourtec:DryRunState;
   OntoLab:stateLastUpdatedAt 1678887921;
.

# vapourtec r2 pump - only present one here as an example
_lab2:VapourtecR2_1
   rdf:type OntoVapourtec:VapourtecR2Pump;
   OntoVapourtec:locationID "A"^^xsd:string;
   OntoVapourtec:hasReagentSource _lab2:ReagentBottle_1; # this pump is
       sourcing from reagent bottle
.
```

In the context of chemical experiments, it is common to use an autosampler to source chemicals from multiple vials, each with a limited volume. For starting materials that are prepared in large amounts, the concentration of chemicals can be the same across multiple vials. Supplementary Triples S7 presents an example where three vials contain a solution for benzaldehyde. This representation allows for the dynamic selection of the chemical source in such cases, especially when deciding which vial to be excluded if there is not enough amount. This representation can also be useful for categorical optimisation, such as when the type of catalyst is a design variable.

Supplementary Triples S7: *Example instantiation for autosampler sites in Cambridge lab using OntoVapourtec.*

```
# autosampler - chemical source from autosampler sites
_lab1:AutoSampler
    rdf:type OntoVapourtec:AutoSampler;
    OntoVapourtec:hasSite _lab1:Site_1;
    OntoVapourtec:hasSite _lab1:Site_2;
    OntoVapourtec:hasSite _lab1:Site_3;
.

# autosampler sites
_lab1:Site_1
    a OntoVapourtec:AutoSamplerSite;
    OntoVapourtec:locationID "1"^^xsd:string;
    OntoVapourtec:holds _lab1:Vial_1;
.

_lab1:Site_2
    a OntoVapourtec:AutoSamplerSite;
    OntoVapourtec:locationID "2"^^xsd:string;
    OntoVapourtec:holds _lab1:Vial_2;
.

_lab1:Site_3
    rdf:type OntoVapourtec:AutoSamplerSite;
    OntoVapourtec:locationID "3"^^xsd:string;
    OntoVapourtec:holds _lab1:Vial_3;
.

# vials
_lab1:Vial_2
    a OntoLab:Vial;
    OntoLab:isFilledWith _lab1:ChemicalAmount_2;
    OntoLab:hasMaxLevel _lab1:Vial_2_level_max; # omitted here for simplicity
    OntoLab:hasWarningLevel _lab1:Vial_2_level_warning;
    OntoLab:hasFillLevel _lab1:Vial_2_level_fill;
.

_lab1:Vial_2_level_warning
   rdf:type om:Volume;
   om:hasValue _lab1:Vial_2_level_warning_value;
.

_lab1:Vial_2_level_warning_value
   rdf:type om:Measure;
   om:hasUnit om:millilitre;
   om:hasNumericalValue "5"^^xsd:double;
.

_lab1:Vial_2_level_fill
    rdf:type om:Volume;
    om:hasValue _lab1:Vial_2_level_fill_value;
.

_lab1:Vial_2_level_fill_value
    rdf:type om:Measure;
    om:hasUnit om:millilitre;
    om:hasNumericalValue "35"^^xsd:double;
```

```
.

_lab1:Vial_3
    rdf:type OntoLab:Vial;
    OntoLab:isFilledWith _lab1:ChemicalAmount_3;
    OntoLab:hasMaxLevel _lab1:Vial_3_level_max; # omitted here for simplicity
    OntoLab:hasWarningLevel _lab1:Vial_3_level_warning;
    OntoLab:hasFillLevel _lab1:Vial_3_level_fill;
.

_lab1:Vial_3_level_warning
   rdf:type om:Volume;
   om:hasValue _lab1:Vial_3_level_warning_value;
.

_lab1:Vial_3_level_warning_value
   rdf:type om:Measure;
   om:hasUnit om:millilitre;
   om:hasNumericalValue "5"^^xsd:double;
.

_lab1:Vial_3_level_fill
    rdf:type om:Volume;
    om:hasValue _lab1:Vial_3_level_fill_value;
.

_lab1:Vial_3_level_fill_value
    rdf:type om:Measure;
    om:hasUnit om:millilitre;
    om:hasNumericalValue "35"^^xsd:double;
.

# chemical amount - _lab1:ChemicalAmount_1 is already listed in the previous example instantiation
_lab1:ChemicalAmount_2
    rdf:type OntoLab:ChemicalAmount;
    OntoCAPE_Behavior:refersToMaterial _lab1:Chemical_1;
    OntoLab:containsUnidentifiedComponent "false"^^xsd:boolean;
.

_lab1:ChemicalAmount_3
    rdf:type OntoLab:ChemicalAmount;
    OntoCAPE_Behavior:refersToMaterial _lab1:Chemical_1;
    OntoLab:containsUnidentifiedComponent "false"^^xsd:boolean;
.
```

A.1.7 OntoHPLC

Key design considerations OntoHPLC (v1.8) is an ontology designed to provide a simplified representation of HPLC. Its goal is not to provide a full-fledged representation of HPLC, as the Vapourtec `FlowCommander` already "automates" the HPLC analysis by sending a serial command at the peak of the reaction steady-state stream as a trigger for sample injection. Instead, OntoHPLC demonstrates the proof-of-concept for the knowledge graph approach with a focus on `ChromatogramPoints` captured in the `HPLCReport`. The components presented in the reaction outlet stream can be identified by matching the retention time at

which the peak appears with those documented in the `HPLCMethod`. Moreover, the raw HPLC reports are also preserved in the knowledge graph via `remoteFilePath`, which points to a remote file server for easy human inspection.

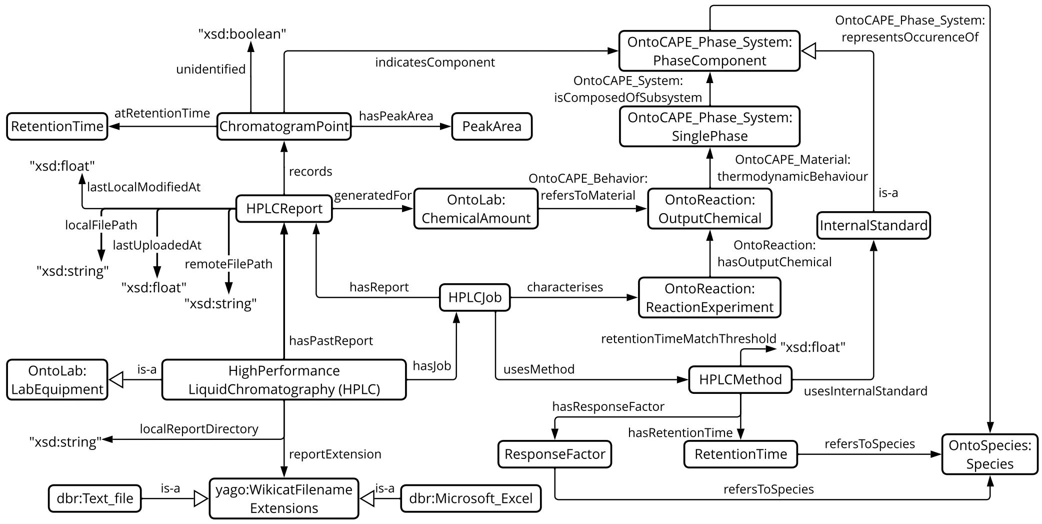

Supplementary Figure S6: *OntoHPLC ontology for HPLC. The relationship with hollow arrow "is-a" represents* `rdfs:subClassOf`*. The remaining concepts and relationships are under the OntoHPLC namespace if not stated otherwise.*

Domain competency questions

1. What is the file format of the HPLC report for a given HPLC?
2. Which HPLC method is used to generate a given HPLC report?
3. What is the retention time of a given chemical species when using a given HPLC method?
4. What is the response factor of a given chemical species when using a given HPLC method?
5. How many chemical species can be identified using a given HPLC method?
6. How many HPLC jobs have been conducted on the given HPLC?
7. What internal standard is used in the given HPLC method?
8. How many chemical species are identified from a given HPLC report?
9. Where is the HPLC report located?
10. What reaction conditions are associated with a given HPLC report?
11. Are there any side products identified from a given HPLC report?
12. Are these any unidentified peaks that have significant peak areas?

Example instantiation Supplementary Triples S8 presents a snippet of instantiation for HPLC systems. The HPLC method is simplified to include only the target product and internal standard. When processing the HPLC report, a peak is considered matched to a species if its retention time falls within the matching threshold specified in the HPLC method. For example, the peak collected at 6.630 minutes is within 6.611 ± 0.10 minutes, therefore is identified as the target product.

Supplementary Triples S8: *Example instantiation for HPLC in both Cambridge and Singapore lab using OntoHPLC.*

```
## Cambridge lab - HPLC
# HPLC
_lab1:Shimadzu_HPLC
   rdf:type OntoHPLC:HighPerformanceLiquidChromatography;
   OntoHPLC:localReportDirectory "/app/CHEM32/"^^xsd:string;
   OntoHPLC:reportExtension dbr:Text_file;
   OntoHPLC:hasJob _lab1:HPLC_Job_1;
   OntoLab:isManagedBy _agent:HPLCAgent_Lab1;
.

# HPLC method
_lab1:HPLCMethod_Aldol
   rdf:type OntoHPLC:HPLCMethod;
   # retention time
   OntoHPLC:hasRetentionTime _lab1:HPLCMethod_RetentionTime_1;
   OntoHPLC:hasRetentionTime _lab1:HPLCMethod_RetentionTime_2;
   # response factor
   OntoHPLC:hasResponseFactor _lab1:HPLCMethod_ResponseFactor_1;
   OntoHPLC:hasResponseFactor _lab1:HPLCMethod_ResponseFactor_2;
   # internal standard
   OntoHPLC:usesInternalStandard _lab1:InternalStandard;
   # threshold for species matching
   OntoHPLC:retentionTimeMatchThreshold "0.10"^^xsd:double;
.

# HPLC method - retention time 1 for product benzalacetone
_lab1:HPLCMethod_RetentionTime_1
   rdf:type OntoHPLC:RetentionTime;
   OntoHPLC:refersToSpecies <http://www.theworldavatar.com/kg/ontospecies/
       Species_f999de28-55dc-477e-8afc-e8802064e0d2>;
   om:hasValue _lab1:HPLCMethod_RetentionTime_1_value;
.

_lab1:HPLCMethod_RetentionTime_1_value
   rdf:type om:Measure;
   om:hasUnit om:minute-Time;
   om:hasNumericalValue "6.611"^^xsd:double;
.

# HPLC method - retention time 2 for internal standard
_lab1:HPLCMethod_RetentionTime_2
   rdf:type OntoHPLC:RetentionTime;
   OntoHPLC:refersToSpecies <http://www.theworldavatar.com/kg/ontospecies/
       Species_471ce681-98f3-4245-be7d-3f38dfb6dd22>;
   om:hasValue _lab1:HPLCMethod_RetentionTime_2_value;
.
```

```
_lab1:HPLCMethod_RetentionTime_2_value
   rdf:type om:Measure;
   om:hasUnit om:minute-Time;
   om:hasNumericalValue "13.300"^^xsd:double;
.

# HPLC method - internal standard
_lab1:InternalStandard
   rdf:type OntoHPLC:InternalStandard;
   OntoCAPE_Phase_System:representsOccurenceOf <http://www.theworldavatar.com/
       kg/ontospecies/Species_471ce681-98f3-4245-be7d-3f38dfb6dd22>;
   OntoCAPE_System:hasProperty _lab1:InternalStandard_Property_1;
.

_lab1:InternalStandard_Property_1
   rdf:type OntoCAPE_Phase_System:Molarity;
   OntoCAPE_System:hasValue _lab1:InternalStandard_Property_1_ScalarValue_1;
.

_lab1:InternalStandard_Property_1_ScalarValue_1
   rdf:type OntoCAPE_System:ScalarValue;
   OntoCAPE_System:hasUnitOfMeasure om:molePerLitre;
   OntoCAPE_System:numericalValue "0.06"^^xsd:double;
.

# HPLC job
_lab1:HPLC_Job_1
   rdf:type OntoHPLC:HPLCJob;
   OntoHPLC:hasReport _lab1:HPLCReport_1;
   OntoHPLC:usesMethod _lab1:HPLCMethod_Aldol;
   OntoHPLC:characterises _exp:ReactionExperiment_0;
.

# HPLC report
_lab1:HPLCReport_1
   rdf:type OntoHPLC:HPLCReport;
   # chromatogram points
   OntoHPLC:records _lab1:ChromatogramPoint_1;
   OntoHPLC:records _lab1:ChromatogramPoint_2;
   # information about file server
   OntoHPLC:lastLocalModifiedAt 1673623824;
   OntoHPLC:lastUploadedAt 1673623826;
   OntoHPLC:localFilePath "/app/CHEM32/ASCIIData000.txt"^^xsd:string;
   OntoHPLC:remoteFilePath "http://lab1:8080/FileServer/Shimadzu_HPLC/app/
       CHEM32/ASCIIData000_1673623828705.txt"^^xsd:anyURI;
.

# chromatogram point 1
_lab1:ChromatogramPoint_1
   rdf:type OntoHPLC:ChromatogramPoint;
   OntoHPLC:atRetentionTime _lab1:RetentionTime_1;
   OntoHPLC:hasPeakArea _lab1:PeakArea_1;
.

_lab1:RetentionTime_1
   rdf:type OntoHPLC:RetentionTime;
   om:hasValue _lab1:RetentionTime_1_value;
.

```

```
_lab1:RetentionTime_1_value
   rdf:type om:Measure;
   om:hasUnit om:minute-Time;
   om:hasNumericalValue "6.630"^^xsd:double;
.

# chromatogram point 2
_lab1:ChromatogramPoint_2
   rdf:type OntoHPLC:ChromatogramPoint;
   OntoHPLC:atRetentionTime _lab1:RetentionTime_2;
   OntoHPLC:hasPeakArea _lab1:PeakArea_2;
.

_lab1:RetentionTime_2
   rdf:type OntoHPLC:RetentionTime;
   om:hasValue _lab1:RetentionTime_2_value;
.

_lab1:RetentionTime_2_value
   rdf:type om:Measure;
   om:hasUnit om:minute-Time;
   om:hasNumericalValue "13.363"^^xsd:double;
.

## Singapore lab - HPLC
# HPLC
_lab2:Agilent_HPLC
   rdf:type OntoHPLC:HighPerformanceLiquidChromatography;
   OntoHPLC:localReportDirectory "/app/CHEM32/"^^xsd:string;
   OntoHPLC:reportExtension dbr:Microsoft_Excel;
   OntoLab:isManagedBy _agent:HPLCAgent_Lab2;
.
```

A.1.8 OntoDerivation

Rationale for adoption OntoDerivation (v1.10) is an ontological markup developed as part of the derived information framework for a dynamic knowledge graph. For detailed documentation please refer to Bai et al. [19]. In this work, OntoDerivation is employed to instantiate provenance records for both data and material flow in the design-make-test-analyse (DMTA) cycle. It does so by marking up each step in the workflow as an instance of `DerivationAsyn` with the instance corresponding to its previous step as input. For the very first step in the workflow, *i.e.* DoE, the instance of `OntoDoE:DesignOfExperiment` is marked as input. This forms a chain of asynchronous job requests with their data dependencies recorded in the knowledge graph. The outputs of each job in the workflow will be populated back to the knowledge graph at its completion and trigger the next corresponding agent. By utilising OntoDerivation, queries can be performed to track the progress of the iterative workflow.

Domain competency questions

1. When was a given job started?
2. What is the status of a given job?

3. Is there any prior experiment in the queue for execution before a given reaction experiment?
4. Has the HPLC report been generated for the HPLC job?
5. Has the characterisation results of the given reaction experiment been post-processed to calculate yield?
6. What steps are left to be completed for the current DMTA cycle of a given reaction experiment?

Example instantiation Supplementary Triples S9 instantiates one iteration in a DMTA cycle starting from the DoE instance presented in Supplementary Triples S4. As no outputs are generated at the time of job requests, all derivation instances are initialised with a timestamp of 0, which will be replaced with the starting timestamp of the job at its completion. The outputs are also populated back to the derivation chain as the workflow progresses. The detailed stepping is discussed in Supplementary Section A.2.10.

Supplementary Triples S9: *Example instantiation for a DMTA cycle request using OntoDerivation.*

```
## DoE job request
_derivation:DoEDerivation_i1
   rdf:type OntoDerivation:DerivationAsyn;
   OntoDerivation:isDerivedFrom _doe:DesignOfExperiment_1;
   OntoDerivation:isDerivedFrom _lab1:Lab_1;
   OntoDerivation:isDerivedUsing _agent:DoEAgent;
   OntoDerivation:hasStatus _derivation:DoE_1_status;
   time:hasTime _derivation:DoE_1_time;
.

_derivation:DoE_1_time
   rdf:type time:Instant;
   time:inTimePosition _derivation:DoE_1_time_position;
.

_derivation:DoE_1_time_position
   rdf:type time:TimePosition;
   time:hasTRS <http://dbpedia.org/resource/Unix_time>;
   time:numericPosition 0;
.

_derivation:DoE_1_status
   rdf:type OntoDerivation:Requested;
.

## Scheduling job request
_derivation:ScheduleDerivation_i1
   rdf:type OntoDerivation:DerivationAsyn;
   OntoDerivation:isDerivedFrom _derivation:DoEDerivation_i1;
   OntoDerivation:isDerivedFrom _lab1:Lab_1;
   OntoDerivation:isDerivedUsing _agent:VapourtecScheduleAgent;
   OntoDerivation:hasStatus _derivation:Schedule_1_status;
   time:hasTime _derivation:Schedule_1_time;
.

_derivation:Schedule_1_time
```

```
    rdf:type time:Instant;
    time:inTimePosition _derivation:Schedule_1_time_position;
.

_derivation:Schedule_1_time_position
    rdf:type time:TimePosition;
    time:hasTRS <http://dbpedia.org/resource/Unix_time>;
    time:numericPosition 0;
.

_derivation:Schedule_1_status
    rdf:type OntoDerivation:Requested;
.

## Post-processing job request
_derivation:PostProcessingDerivation_i1
    rdf:type OntoDerivation:DerivationAsyn;
    OntoDerivation:isDerivedFrom _derivation:ScheduleDerivation_i1;
    OntoDerivation:isDerivedUsing _agent:HPLCPostProAgent;
    OntoDerivation:hasStatus _derivation:PostProcessing_1_status;
    time:hasTime _derivation:PostProcessing_1_time;
.

_derivation:PostProcessing_1_time
    rdf:type time:Instant;
    time:inTimePosition _derivation:PostProcessing_1_time_position;
.

_derivation:PostProcessing_1_time_position
    rdf:type time:TimePosition;
    time:hasTRS <http://dbpedia.org/resource/Unix_time>;
    time:numericPosition 0;
.

_derivation:PostProcessing_1_status
    rdf:type OntoDerivation:Requested;
.

## Reaction job request to Cambridge lab - assume that _doe:
     DesignOfExperiment_1 is finished by _agent:DoEAgent and suggested _doe:
     ReactionExperiment_i1
_derivation:VapourtecDerivation_i1
    rdf:type OntoDerivation:DerivationAsyn;
    OntoDerivation:isDerivedFrom _doe:ReactionExperiment_i1;
    OntoDerivation:isDerivedUsing _agent:VapourtecAgent_Lab1;
    OntoDerivation:hasStatus _derivation:Vapourtec_1_status;
    time:hasTime _derivation:Vapourtec_1_time;
.

_derivation:Vapourtec_1_time
    rdf:type time:Instant;
    time:inTimePosition _derivation:Vapourtec_1_time_position;
.

_derivation:Vapourtec_1_time_position
    rdf:type time:TimePosition;
    time:hasTRS <http://dbpedia.org/resource/Unix_time>;
    time:numericPosition 0;
.
```

```

_derivation:Vapourtec_1_status
   rdf:type OntoDerivation:Requested;
.

## Characterisation job request to Cambridge lab - assume that _doe:
    DesignOfExperiment_1 is finished by _agent:DoEAgent and suggested _doe:
    ReactionExperiment_i1
_derivation:HPLCDerivation_i1
   rdf:type OntoDerivation:DerivationAsyn;
   OntoDerivation:isDerivedFrom _doe:ReactionExperiment_i1;
   OntoDerivation:isDerivedFrom _derivation:VapourtecDerivation_i1;
   OntoDerivation:isDerivedUsing _agent:HPLCAgent_Lab1;
   OntoDerivation:hasStatus _derivation:HPLC_1_status;
   time:hasTime _derivation:HPLC_1_time;
.

_derivation:HPLC_1_time
   rdf:type time:Instant;
   time:inTimePosition _derivation:HPLC_1_time_position;
.

_derivation:HPLC_1_time_position
   rdf:type time:TimePosition;
   time:hasTRS <http://dbpedia.org/resource/Unix_time>;
   time:numericPosition 0;
.

_derivation:HPLC_1_status
   rdf:type OntoDerivation:Requested;
.
```

A.1.9 OntoGoal

Key design considerations OntoGoal (v1.3) is an ontological markup for the process of pursuing research goals by autonomous agents. It is inspired by a type of rational agent architecture, namely the belief–desire-intention (BDI) [20, 21] architecture rooted in cognitive psychology theory developed by Bratman [22]. In their definition, ‘beliefs’ are the agent’s knowledge about itself and its surrounding environment, ‘desires’ are objectives that they would like to achieve, and ‘intentions’ are possible actions that can be adopted to achieve the committed goal. Specifically, ‘goal’ is an instantiation of the ‘desire’ but with more emphasis on the achievable side and ‘plan’ is a concrete realisation of ‘intentions‘ as a sequence of actions.

Following these definitions, we construct a `GoalSet` to accommodate individual `Goal` (objective) from the multi-objective optimisation problem. Each goal desires certain dimensional quantities and can be achieved by a `Plan` which consists of multiple `Step` that each can be performed by a corresponding agent. When the plan is executed, the state of the world changes. The beliefs, in this case `OntoReaction:ReactionExperiment`, are updated and reflected as historical data in the next iteration. In practice, the resources available to pursue a goal are almost always limited. The current design incorporates the `cycleAllowance` and `deadline` as two limiting factors. Future development can be made to take the classic metric when bench-marking the performance of optimisation algorithms

into consideration [17], *i.e.* improvement of the hypervolume.

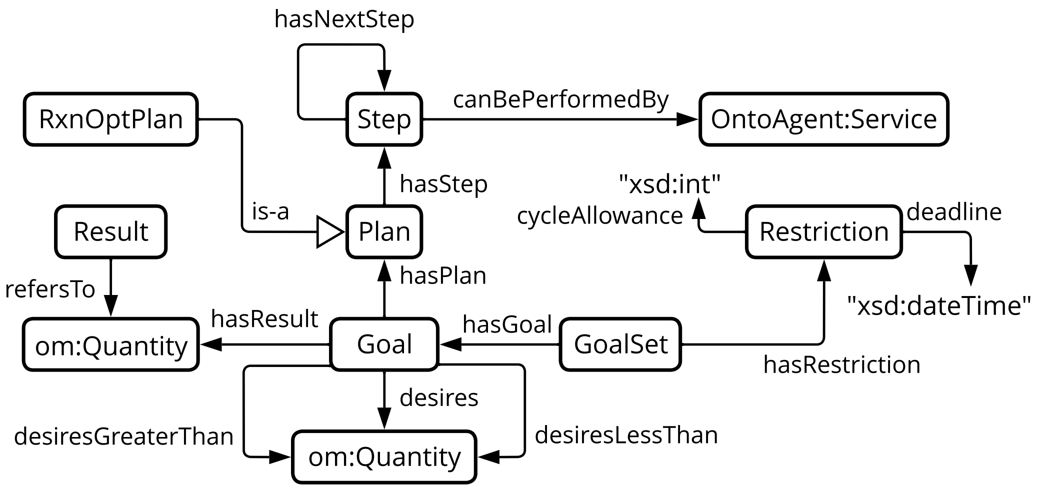

Supplementary Figure S7: *OntoGoal ontology for research goals. The relationship with hollow arrow "is-a" represents* `rdfs:subClassOf`*. The remaining concepts and relationships are under the OntoGoal namespace if not stated otherwise.*

Domain competency questions

1. What is the plan that can be employed to achieve a given goal?
2. Which agent can be employed for a given step in a plan?
3. How many goals (objectives) does the given goal set have?
4. How many iterations have been done?
5. Is there still enough iteration allowance for pursuing a given goal?
6. When is the deadline for pursuing a given goal?
7. Has a goal been met?
8. Is the best performance of a chemical reaction meeting the research goal?

Example instantiation Supplementary Triples S10 lists an example plan that can be adopted for closed-loop optimisation. Notably, agents that manage the individual equipment in the lab are not included in the plan as they are delegated by the agent that performs the scheduling.

Supplementary Triples S10: *Example instantiation for an optimisation plan using OntoGoal.*

```
## optimisation plan
_goal:ReactionOptimisationPlan
   rdf:type OntoGoal:RxnOptPlan;
   OntoGoal:hasStep _goal:DoE_Step;
   OntoGoal:hasStep _goal:Schedule_Step;
```

```
    OntoGoal:hasStep _goal:PostProcessing_Step;
.

_goal:DoE_Step
    rdf:type OntoGoal:DesignOfExperiment;
    OntoGoal:hasNextStep _goal:Schedule_Step;
    OntoGoal:canBePerformedBy _agent:DoEAgent;
.

_goal:Schedule_Step
    rdf:type OntoGoal:RxnExpExecution;
    OntoGoal:hasNextStep _goal:PostProcessing_Step;
    OntoGoal:canBePerformedBy _agent:VapourtecScheduleAgent;
.

_goal:PostProcessing_Step
    rdf:type OntoGoal:PostProcessing;
    OntoGoal:canBePerformedBy _agent:HPLCPostProAgent;
.
```

Supplementary Triples S11 presents a goal set instantiated upon a goal request from a scientist. The request comes in as a multi-objective optimisation problem which has a target of reaching run material cost below 200 £ L^{-1} and reaction yield above 80%. The restrictions are 50 iterations before the end of 2023 Greenwich Mean Time, whichever comes earlier.

Supplementary Triples S11: *Example instantiation for a goal set upon goal request from scientists using OntoGoal.*

```
## goal set
_goal:GoalSet_1
    rdf:type OntoGoal:GoalSet;
    OntoGoal:hasGoal _goal:Goal_1;
    OntoGoal:hasGoal _goal:Goal_2;
    OntoGoal:hasRestriction _goal:Restriction_1;
.

## the first goal
_goal:Goal_1
    rdf:type OntoGoal:Goal;
    OntoGoal:desiresGreaterThan _goal:Desired_Quantity_1;
    OntoGoal:hasPlan _goal:ReactionOptimisationPlan;
.

_goal:Desired_Quantity_1
    rdf:type OntoReaction:Yield;
    om:hasValue _goal:Desired_Quantity_Measure_1;
.

_goal:Desired_Quantity_Measure_1
    rdf:type om:Measure;
    om:hasUnit om:percent;
    om:hasNumericalValue "80"^^xsd:double;
.

## the second goal
_goal:Goal_2
    rdf:type OntoGoal:Goal;
```

```
    OntoGoal:desiresLessThan _goal:Desired_Quantity_2;
    OntoGoal:hasPlan _goal:ReactionOptimisationPlan;
.

_goal:Desired_Quantity_2
    rdf:type OntoReaction:RunMaterialCost;
    om:hasValue _goal:Desired_Quantity_Measure_2;
.

_goal:Desired_Quantity_Measure_2
    rdf:type om:Measure;
    om:hasUnit om:poundSterlingPerLitre;
    om:hasNumericalValue "200"^^xsd:double;
.

## restriction
_goal:Restriction_1
    rdf:type OntoGoal:Restriction;
    OntoGoal:cycleAllowance 50;
    OntoGoal:deadline "2023-12-31T23:59:59+0000"^^xsd:dateTime;
.
```

A.1.10 Object mapping and type validation

As demonstrated in the example instantiation, the knowledge graph is highly intertwined. It can be tedious also error-prone to write boilerplate codes to process the knowledge statements if the same data is required in different agents. Therefore, we implement object mapping and type validation as a persistence layer between the knowledge graph and the agents. Utility functions are provided as constructors for the ontological concepts so that the agents (hence their developers) can deal with canonical Python objects directly. For technical details please refer to `chemistry-and-robots` Python package [23].

A.1.11 Description logic representation

The description logic representations of the ontologies developed in this work are provided below.

A.1.11.1 OntoReaction

Classes:

OntoReaction:Base $\sqsubseteq$ OntoKin:Species
OntoReaction:Catalyst $\sqsubseteq$ OntoKin:Species
OntoReaction:Chemical $\sqsubseteq$ OntoCAPE_Material:Material
OntoReaction:ChemicalReaction $\sqsubseteq$ OntoCAPE_Reaction_Mechanism:ChemicalReaction
OntoReaction:Conversion $\sqsubseteq$ om:AmountOfSubstanceFraction
OntoReaction:Conversion $\sqsubseteq$ OntoReaction:PerformanceIndicator
OntoReaction:Conversion $\sqsubseteq = 1$ OntoReaction:yieldLimitingSpecies.OntoSpecies:Species
OntoReaction:EcoScore $\sqsubseteq$ om:QuantityOfDimensionOne
OntoReaction:EcoScore $\sqsubseteq$ OntoReaction:PerformanceIndicator
OntoReaction:EnvironmentalFactor $\sqsubseteq$ om:MassFraction

OntoReaction:EnvironmentalFactor ⊑ OntoReaction:PerformanceIndicator
OntoReaction:Impurity ⊑ OntoKin:Product
OntoReaction:InputChemical ⊑ OntoReaction:Chemical
OntoReaction:OutputChemical ⊑ OntoReaction:Chemical
OntoReaction:ReactionExperiment ⊑ = 1 OntoReaction:hasResTime.OntoReaction:ResidenceTime
OntoReaction:ReactionExperiment ⊑ = 1 OntoReaction:hasRxnPressure.OntoReaction:ReactionPressure
OntoReaction:ReactionExperiment ⊑ = 1 OntoReaction:hasRxnScale.OntoReaction:ReactionScale
OntoReaction:ReactionExperiment ⊑ = 1 OntoReaction:hasRxnTemperature.OntoReaction:ReactionTemperature
OntoReaction:ReactionExperiment ⊑ = 1 OntoReaction:isOccurenceOf.OntoReaction:ChemicalReaction
OntoReaction:ReactionPressure ⊑ om:Pressure
OntoReaction:ReactionPressure ⊑ OntoReaction:ReactionCondition
OntoReaction:ReactionScale ⊑ om:Volume
OntoReaction:ReactionScale ⊑ OntoReaction:ReactionCondition
OntoReaction:ReactionScale ⊑ = 1 OntoReaction:indicatesUsageOf.OntoReaction:InputChemical
OntoReaction:ReactionTemperature ⊑ om:CelsiusTemperature
OntoReaction:ReactionTemperature ⊑ OntoReaction:ReactionCondition
OntoReaction:ReactionVariation ⊑ OntoReaction:ReactionExperiment
OntoReaction:ReactionVariation ⊑ = 1 OntoReaction:isVariationOf.OntoReaction:ReactionExperiment
OntoReaction:ResidenceTime ⊑ om:Duration
OntoReaction:ResidenceTime ⊑ OntoReaction:ReactionCondition
OntoReaction:RunMaterialCost ⊑ om:SpecificAmountOfMoney
OntoReaction:RunMaterialCost ⊑ OntoReaction:PerformanceIndicator
OntoReaction:RunMaterialCostPerKilogramProduct ⊑ om:SpecificAmountOfMoney
OntoReaction:RunMaterialCostPerKilogramProduct ⊑ OntoReaction:PerformanceIndicator
OntoReaction:Solvent ⊑ OntoKin:Species
OntoReaction:SpaceTimeYield ⊑ om:Quantity
OntoReaction:SpaceTimeYield ⊑ OntoReaction:PerformanceIndicator
OntoReaction:StoichiometryRatio ⊑ om:VolumeFraction
OntoReaction:StoichiometryRatio ⊑ OntoReaction:ReactionCondition
OntoReaction:StoichiometryRatio ⊑ = 1 OntoReaction:indicatesMultiplicityOf.OntoReaction:InputChemical
OntoReaction:TargetProduct ⊑ OntoKin:Product
OntoReaction:Yield ⊑ om:AmountOfSubstanceFraction
OntoReaction:Yield ⊑ OntoReaction:PerformanceIndicator
OntoReaction:Yield ⊑ = 1 OntoReaction:yieldLimitingSpecies.OntoSpecies:Species

Object properties:

OntoReaction:hasCatalyst ⊑ OntoCAPE_Reaction_Mechanism:hasCatalyst
OntoReaction:hasConversion ⊑ OntoReaction:hasPerformanceIndicator
OntoReaction:hasEcoScore ⊑ OntoReaction:hasPerformanceIndicator

OntoReaction:hasEnvironmentalFactor $\sqsubseteq$ OntoReaction:hasPerformanceIndicator
OntoReaction:hasResTime $\sqsubseteq$ OntoReaction:hasReactionCondition
OntoReaction:hasRunMaterialCost $\sqsubseteq$ OntoReaction:hasPerformanceIndicator
OntoReaction:hasRunMaterialCostPerKilogramProduct $\sqsubseteq$ OntoReaction:hasPerformanceIndicator
OntoReaction:hasRxnPressure $\sqsubseteq$ OntoReaction:hasReactionCondition
OntoReaction:hasRxnScale $\sqsubseteq$ OntoReaction:hasReactionCondition
OntoReaction:hasRxnTemperature $\sqsubseteq$ OntoReaction:hasReactionCondition
OntoReaction:hasSpaceTimeYield $\sqsubseteq$ OntoReaction:hasPerformanceIndicator
OntoReaction:hasStoichiometryRatio $\sqsubseteq$ OntoReaction:hasReactionCondition
OntoReaction:hasYield $\sqsubseteq$ OntoReaction:hasPerformanceIndicator
$\exists$ OntoReaction:hasBase.$\top$ $\sqsubseteq$ OntoReaction:ChemicalReaction
$\exists$ OntoReaction:hasCatalyst.$\top$ $\sqsubseteq$ OntoReaction:ChemicalReaction
$\exists$ OntoReaction:hasConversion.$\top$ $\sqsubseteq$ OntoReaction:ReactionExperiment
$\exists$ OntoReaction:hasEcoScore.$\top$ $\sqsubseteq$ OntoReaction:ReactionExperiment
$\exists$ OntoReaction:hasEnvironmentalFactor.$\top$ $\sqsubseteq$ OntoReaction:ReactionExperiment
$\exists$ OntoReaction:hasInputChemical.$\top$ $\sqsubseteq$ OntoReaction:ReactionExperiment
$\exists$ OntoReaction:hasOutputChemical.$\top$ $\sqsubseteq$ OntoReaction:ReactionExperiment
$\exists$ OntoReaction:hasPerformanceIndicator.$\top$ $\sqsubseteq$ OntoReaction:ReactionExperiment
$\exists$ OntoReaction:hasReactionCondition.$\top$ $\sqsubseteq$ OntoReaction:ReactionExperiment
$\exists$ OntoReaction:hasResTime.$\top$ $\sqsubseteq$ OntoReaction:ReactionExperiment
$\exists$ OntoReaction:hasRunMaterialCost.$\top$ $\sqsubseteq$ OntoReaction:ReactionExperiment
$\exists$ OntoReaction:hasRunMaterialCostPerKilogramProduct.$\top$ $\sqsubseteq$ OntoReaction:ReactionExperiment
$\exists$ OntoReaction:hasRxnPressure.$\top$ $\sqsubseteq$ OntoReaction:ReactionExperiment
$\exists$ OntoReaction:hasRxnScale.$\top$ $\sqsubseteq$ OntoReaction:ReactionExperiment
$\exists$ OntoReaction:hasRxnTemperature.$\top$ $\sqsubseteq$ OntoReaction:ReactionExperiment
$\exists$ OntoReaction:hasRxnType.$\top$ $\sqsubseteq$ OntoReaction:ChemicalReaction
$\exists$ OntoReaction:hasSolvent.$\top$ $\sqsubseteq$ OntoReaction:ChemicalReaction
$\exists$ OntoReaction:hasSpaceTimeYield.$\top$ $\sqsubseteq$ OntoReaction:ReactionExperiment
$\exists$ OntoReaction:hasStoichiometryRatio.$\top$ $\sqsubseteq$ OntoReaction:ReactionExperiment
$\exists$ OntoReaction:hasYield.$\top$ $\sqsubseteq$ OntoReaction:ReactionExperiment
$\exists$ OntoReaction:indicatesMultiplicityOf.$\top$ $\sqsubseteq$ OntoReaction:StoichiometryRatio
$\exists$ OntoReaction:indicatesUsageOf.$\top$ $\sqsubseteq$ OntoReaction:ReactionScale
$\exists$ OntoReaction:isAssignedTo.$\top$ $\sqsubseteq$ OntoReaction:ReactionExperiment
$\exists$ OntoReaction:isOccurenceOf.$\top$ $\sqsubseteq$ OntoReaction:ReactionExperiment
$\exists$ OntoReaction:isVariationOf.$\top$ $\sqsubseteq$ OntoReaction:ReactionVariation
$\exists$ OntoReaction:yieldLimitingSpecies.$\top$ $\sqsubseteq$ (OntoReaction:Conversion $\sqcup$ OntoReaction:Yield)
$\top$ $\sqsubseteq$ $\forall$ OntoReaction:hasBase.OntoReaction:Base
$\top$ $\sqsubseteq$ $\forall$ OntoReaction:hasCatalyst.OntoReaction:Catalyst
$\top$ $\sqsubseteq$ $\forall$ OntoReaction:hasConversion.OntoReaction:Conversion
$\top$ $\sqsubseteq$ $\forall$ OntoReaction:hasEcoScore.OntoReaction:EcoScore
$\top$ $\sqsubseteq$ $\forall$ OntoReaction:hasEnvironmentalFactor.OntoReaction:EnvironmentalFactor
$\top$ $\sqsubseteq$ $\forall$ OntoReaction:hasInputChemical.OntoReaction:InputChemical
$\top$ $\sqsubseteq$ $\forall$ OntoReaction:hasOutputChemical.OntoReaction:OutputChemical
$\top$ $\sqsubseteq$ $\forall$ OntoReaction:hasPerformanceIndicator.OntoReaction:PerformanceIndicator
$\top$ $\sqsubseteq$ $\forall$ OntoReaction:hasReactionCondition.OntoReaction:ReactionCondition

$\top \sqsubseteq \forall$ OntoReaction:hasResTime.OntoReaction:ResidenceTime
$\top \sqsubseteq \forall$ OntoReaction:hasRunMaterialCost.OntoReaction:RunMaterialCost
$\top \sqsubseteq \forall$ OntoReaction:hasRunMaterialCostPerKilogramProduct.OntoReaction:RunMaterialCostPerKilogramProduct
$\top \sqsubseteq \forall$ OntoReaction:hasRxnPressure.OntoReaction:ReactionPressure
$\top \sqsubseteq \forall$ OntoReaction:hasRxnScale.OntoReaction:ReactionScale
$\top \sqsubseteq \forall$ OntoReaction:hasRxnTemperature.OntoReaction:ReactionTemperature
$\top \sqsubseteq \forall$ OntoReaction:hasRxnType.RXNO:MolecularProcess
$\top \sqsubseteq \forall$ OntoReaction:hasSolvent.OntoReaction:Solvent
$\top \sqsubseteq \forall$ OntoReaction:hasSpaceTimeYield.OntoReaction:SpaceTimeYield
$\top \sqsubseteq \forall$ OntoReaction:hasStoichiometryRatio.OntoReaction:StoichiometryRatio
$\top \sqsubseteq \forall$ OntoReaction:hasYield.OntoReaction:Yield
$\top \sqsubseteq \forall$ OntoReaction:indicatesMultiplicityOf.OntoReaction:InputChemical
$\top \sqsubseteq \forall$ OntoReaction:indicatesUsageOf.OntoReaction:InputChemical
$\top \sqsubseteq \forall$ OntoReaction:isAssignedTo.OntoVapourtec:VapourtecR4Reactor
$\top \sqsubseteq \forall$ OntoReaction:isOccurenceOf.OntoReaction:ChemicalReaction
$\top \sqsubseteq \forall$ OntoReaction:isVariationOf.OntoReaction:ReactionExperiment
$\top \sqsubseteq \forall$ OntoReaction:yieldLimitingSpecies.OntoSpecies:Species

Data properties:
$\exists$ OntoReaction:cdXML.$\top \sqsubseteq$ OntoReaction:ChemicalReaction
$\exists$ OntoReaction:hasRDFILE.$\top \sqsubseteq$ OntoReaction:ChemicalReaction
$\exists$ OntoReaction:hasRInChI.$\top \sqsubseteq$ OntoReaction:ChemicalReaction
$\exists$ OntoReaction:ordID.$\top \sqsubseteq$ OntoReaction:ChemicalReaction
$\exists$ OntoReaction:rxnCXSMILES.$\top \sqsubseteq$ OntoReaction:ChemicalReaction
$\exists$ OntoReaction:rxnSMILES.$\top \sqsubseteq$ OntoReaction:ChemicalReaction
$\top \sqsubseteq \forall$ OntoReaction:cdXML.xsd:string
$\top \sqsubseteq \forall$ OntoReaction:hasRDFILE.xsd:string
$\top \sqsubseteq \forall$ OntoReaction:hasRInChI.xsd:string
$\top \sqsubseteq \forall$ OntoReaction:ordID.xsd:string
$\top \sqsubseteq \forall$ OntoReaction:rxnCXSMILES.xsd:string
$\top \sqsubseteq \forall$ OntoReaction:rxnSMILES.xsd:string

A.1.11.2 OntoDoE

Classes:
OntoDoE:CategoricalVariable $\sqsubseteq$ OntoDoE:DesignVariable
OntoDoE:CategoricalVariable $\sqsubseteq = 1$ OntoDoE:refersToQuantity.om:Quantity
OntoDoE:Center $\sqsubseteq$ OntoDoE:Criterion
OntoDoE:CenterMaximum $\sqsubseteq$ OntoDoE:Criterion
OntoDoE:ContinuousVariable $\sqsubseteq$ OntoDoE:DesignVariable
OntoDoE:ContinuousVariable $\sqsubseteq = 1$ OntoDoE:refersToQuantity.om:Quantity
OntoDoE:Correlation $\sqsubseteq$ OntoDoE:Criterion
OntoDoE:DesignOfExperiment $\sqsubseteq \geq 1$ OntoDoE:hasSystemResponse.OntoDoE:SystemResponse
OntoDoE:DesignOfExperiment $\sqsubseteq = 1$ OntoDoE:hasDomain.OntoDoE:Domain

OntoDoE:DesignOfExperiment $\sqsubseteq$ = 1 OntoDoE:usesStrategy.OntoDoE:Strategy
OntoDoE:DesignOfExperiment $\sqsubseteq$ = 1 OntoDoE:utilisesHistoricalData.OntoDoE:Historical Data
OntoDoE:Domain $\sqsubseteq$ $\geq$ 1 OntoDoE:hasDesignVariable.OntoDoE:DesignVariable
OntoDoE:FixedParameter $\sqsubseteq$ = 1 OntoDoE:refersToQuantity.om:Quantity
OntoDoE:LHS $\sqsubseteq$ OntoDoE:Strategy
OntoDoE:Maximum $\sqsubseteq$ OntoDoE:Criterion
OntoDoE:NewSTBO $\sqsubseteq$ OntoDoE:Strategy
OntoDoE:SystemResponse $\sqsubseteq$ = 1 OntoDoE:refersToQuantity.om:Quantity
OntoDoE:TSEMO $\sqsubseteq$ OntoDoE:Strategy

Object properties:
$\exists$ OntoDoE:designsChemicalReaction.$\top$ $\sqsubseteq$ OntoDoE:DesignOfExperiment
$\exists$ OntoDoE:hasDesignVariable.$\top$ $\sqsubseteq$ OntoDoE:Domain
$\exists$ OntoDoE:hasDoETemplate.$\top$ $\sqsubseteq$ OntoReaction:ChemicalReaction
$\exists$ OntoDoE:hasDomain.$\top$ $\sqsubseteq$ OntoDoE:DesignOfExperiment
$\exists$ OntoDoE:hasFixedParameter.$\top$ $\sqsubseteq$ OntoDoE:Domain
$\exists$ OntoDoE:hasSystemResponse.$\top$ $\sqsubseteq$ OntoDoE:DesignOfExperiment
$\exists$ OntoDoE:proposesNewExperiment.$\top$ $\sqsubseteq$ OntoDoE:DesignOfExperiment
$\exists$ OntoDoE:refersToExperiment.$\top$ $\sqsubseteq$ OntoDoE:HistoricalData
$\exists$ OntoDoE:refersToQuantity.$\top$ $\sqsubseteq$ (OntoDoE:CategoricalVariable $\sqcup$ OntoDoE:Continuous Variable $\sqcup$ OntoDoE:FixedParameter $\sqcup$ OntoDoE:SystemResponse)
$\exists$ OntoDoE:usesStrategy.$\top$ $\sqsubseteq$ OntoDoE:DesignOfExperiment
$\exists$ OntoDoE:utilisesHistoricalData.$\top$ $\sqsubseteq$ OntoDoE:DesignOfExperiment
$\top$ $\sqsubseteq$ $\forall$ OntoDoE:designsChemicalReaction.OntoReaction:ChemicalReaction
$\top$ $\sqsubseteq$ $\forall$ OntoDoE:hasDesignVariable.OntoDoE:DesignVariable
$\top$ $\sqsubseteq$ $\forall$ OntoDoE:hasDoETemplate.OntoDoE:DesignOfExperiment
$\top$ $\sqsubseteq$ $\forall$ OntoDoE:hasDomain.OntoDoE:Domain
$\top$ $\sqsubseteq$ $\forall$ OntoDoE:hasFixedParameter.OntoDoE:FixedParameter
$\top$ $\sqsubseteq$ $\forall$ OntoDoE:hasSystemResponse.OntoDoE:SystemResponse
$\top$ $\sqsubseteq$ $\forall$ OntoDoE:proposesNewExperiment.OntoReaction:ReactionExperiment
$\top$ $\sqsubseteq$ $\forall$ OntoDoE:refersToExperiment.OntoReaction:ReactionExperiment
$\top$ $\sqsubseteq$ $\forall$ OntoDoE:refersToQuantity.om:Quantity
$\top$ $\sqsubseteq$ $\forall$ OntoDoE:usesStrategy.OntoDoE:Strategy
$\top$ $\sqsubseteq$ $\forall$ OntoDoE:utilisesHistoricalData.OntoDoE:HistoricalData

Data properties:
$\exists$ OntoDoE:hasLevel.$\top$ $\sqsubseteq$ OntoDoE:CategoricalVariable
$\exists$ OntoDoE:lowerLimit.$\top$ $\sqsubseteq$ OntoDoE:ContinuousVariable
$\exists$ OntoDoE:maximise.$\top$ $\sqsubseteq$ OntoDoE:SystemResponse
$\exists$ OntoDoE:nGenerations.$\top$ $\sqsubseteq$ OntoDoE:TSEMO
$\exists$ OntoDoE:nRetries.$\top$ $\sqsubseteq$ OntoDoE:TSEMO
$\exists$ OntoDoE:nSpectralPoints.$\top$ $\sqsubseteq$ OntoDoE:TSEMO
$\exists$ OntoDoE:numOfNewExp.$\top$ $\sqsubseteq$ OntoDoE:HistoricalData
$\exists$ OntoDoE:populationSize.$\top$ $\sqsubseteq$ OntoDoE:TSEMO
$\exists$ OntoDoE:positionalID.$\top$ $\sqsubseteq$ (OntoDoE:ContinuousVariable $\sqcup$ OntoDoE:FixedParameter $\sqcup$ OntoDoE:SystemResponse)

$\exists$ OntoDoE:seed.$\top \sqsubseteq$ OntoDoE:LHS

$\exists$ OntoDoE:upperLimit.$\top \sqsubseteq$ OntoDoE:ContinuousVariable

$\top \sqsubseteq \forall$ OntoDoE:hasLevel.xsd:string

$\top \sqsubseteq \forall$ OntoDoE:lowerLimit.xsd:float

$\top \sqsubseteq \forall$ OntoDoE:maximise.xsd:boolean

$\top \sqsubseteq \forall$ OntoDoE:nGenerations.xsd:int

$\top \sqsubseteq \forall$ OntoDoE:nRetries.xsd:int

$\top \sqsubseteq \forall$ OntoDoE:nSpectralPoints.xsd:int

$\top \sqsubseteq \forall$ OntoDoE:numOfNewExp.xsd:int

$\top \sqsubseteq \forall$ OntoDoE:populationSize.xsd:int

$\top \sqsubseteq \forall$ OntoDoE:positionalID.xsd:string

$\top \sqsubseteq \forall$ OntoDoE:seed.xsd:int

$\top \sqsubseteq \forall$ OntoDoE:upperLimit.xsd:float

A.1.11.3 OntoLab

Classes:

OntoLab:ChemicalAmount $\sqsubseteq$ OntoCAPE_Behavior:MaterialAmount

OntoLab:ChemicalContainer $\sqsubseteq$ = 1 OntoLab:hasFillLevel.om:Volume

OntoLab:ChemicalContainer $\sqsubseteq$ = 1 OntoLab:hasMaxLevel.om:Volume

OntoLab:ChemicalContainer $\sqsubseteq$ = 1 OntoLab:hasWarningLevel.om:Volume

OntoLab:ChemicalContainer $\sqsubseteq \leq$ 1 OntoLab:isFilledWith.OntoLab:ChemicalAmount

OntoLab:Dried $\sqsubseteq$ OntoLab:PreparationMethod

OntoLab:DurationSetting $\sqsubseteq$ OntoLab:ParameterSetting

OntoLab:ExternalBattery $\sqsubseteq$ OntoLab:PowerSupply

OntoLab:ExternalDC $\sqsubseteq$ OntoLab:PowerSupply

OntoLab:FlowRateSetting $\sqsubseteq$ OntoLab:ParameterSetting

OntoLab:LabEquipment $\sqsubseteq$ saref:Device

OntoLab:LabEquipment $\sqsubseteq \geq$ 1 OntoLab:hasPowerSupply.OntoLab:PowerSupply

OntoLab:LabEquipment $\sqsubseteq \leq$ 1 OntoLab:hasHeight.om:Height

OntoLab:LabEquipment $\sqsubseteq \leq$ 1 OntoLab:hasLength.om:Length

OntoLab:LabEquipment $\sqsubseteq \leq$ 1 OntoLab:hasPrice.om:AmountOfMoney

OntoLab:LabEquipment $\sqsubseteq \leq$ 1 OntoLab:hasWeight.om:BodyMass

OntoLab:LabEquipment $\sqsubseteq \leq$ 1 OntoLab:hasWidth.om:Width

OntoLab:LabEquipment $\sqsubseteq \leq$ 1 OntoLab:isManagedBy.OntoAgent:Service

OntoLab:LithiumBattery $\sqsubseteq$ OntoLab:PowerSupply

OntoLab:NiMHRechargeableBattery $\sqsubseteq$ OntoLab:PowerSupply

OntoLab:ParameterSetting $\sqsubseteq \leq$ 1 OntoLab:hasQuantity.om:Quantity

OntoLab:ReagentBottle $\sqsubseteq$ OntoLab:ChemicalContainer

OntoLab:Repurified $\sqsubseteq$ OntoLab:PreparationMethod

OntoLab:SolarPowerPack $\sqsubseteq$ OntoLab:PowerSupply

OntoLab:Sparged $\sqsubseteq$ OntoLab:PreparationMethod

OntoLab:SynthesisedInHouse $\sqsubseteq$ OntoLab:PreparationMethod

OntoLab:TemperatureSetting $\sqsubseteq$ OntoLab:ParameterSetting

OntoLab:UsedAsReceived $\sqsubseteq$ OntoLab:PreparationMethod

OntoLab:Vial $\sqsubseteq$ OntoLab:ChemicalContainer

OntoLab:VolumeSetting $\sqsubseteq$ OntoLab:ParameterSetting

OntoLab:WasteBottle $\sqsubseteq$ OntoLab:ChemicalContainer

Object properties:

$\exists$ OntoLab:contains.$\top \sqsubseteq$ OntoLab:Laboratory
$\exists$ OntoLab:hasFillLevel.$\top \sqsubseteq$ OntoLab:ChemicalContainer
$\exists$ OntoLab:hasHeight.$\top \sqsubseteq$ OntoLab:LabEquipment
$\exists$ OntoLab:hasLength.$\top \sqsubseteq$ OntoLab:LabEquipment
$\exists$ OntoLab:hasMaxLevel.$\top \sqsubseteq$ OntoLab:ChemicalContainer
$\exists$ OntoLab:hasPowerSupply.$\top \sqsubseteq$ OntoLab:LabEquipment
$\exists$ OntoLab:hasPrice.$\top \sqsubseteq$ OntoLab:LabEquipment
$\exists$ OntoLab:hasQuantity.$\top \sqsubseteq$ OntoLab:ParameterSetting
$\exists$ OntoLab:hasSetting.$\top \sqsubseteq$ OntoLab:EquipmentSettings
$\exists$ OntoLab:hasWarningLevel.$\top \sqsubseteq$ OntoLab:ChemicalContainer
$\exists$ OntoLab:hasWeight.$\top \sqsubseteq$ OntoLab:LabEquipment
$\exists$ OntoLab:hasWidth.$\top \sqsubseteq$ OntoLab:LabEquipment
$\exists$ OntoLab:isFilledWith.$\top \sqsubseteq$ OntoLab:ChemicalContainer
$\exists$ OntoLab:isManagedBy.$\top \sqsubseteq$ OntoLab:LabEquipment
$\exists$ OntoLab:isPreparedBy.$\top \sqsubseteq$ OntoLab:ChemicalAmount
$\exists$ OntoLab:specifies.$\top \sqsubseteq$ OntoLab:EquipmentSettings
$\exists$ OntoLab:wasGeneratedFor.$\top \sqsubseteq$ OntoLab:EquipmentSettings
$\top \sqsubseteq \forall$ OntoLab:contains.OntoLab:LabEquipment
$\top \sqsubseteq \forall$ OntoLab:hasFillLevel.om:Volume
$\top \sqsubseteq \forall$ OntoLab:hasHeight.om:Height
$\top \sqsubseteq \forall$ OntoLab:hasLength.om:Length
$\top \sqsubseteq \forall$ OntoLab:hasMaxLevel.om:Volume
$\top \sqsubseteq \forall$ OntoLab:hasPowerSupply.OntoLab:PowerSupply
$\top \sqsubseteq \forall$ OntoLab:hasPrice.om:AmountOfMoney
$\top \sqsubseteq \forall$ OntoLab:hasQuantity.om:Quantity
$\top \sqsubseteq \forall$ OntoLab:hasSetting.OntoLab:ParameterSetting
$\top \sqsubseteq \forall$ OntoLab:hasWarningLevel.om:Volume
$\top \sqsubseteq \forall$ OntoLab:hasWeight.om:BodyMass
$\top \sqsubseteq \forall$ OntoLab:hasWidth.om:Width
$\top \sqsubseteq \forall$ OntoLab:isFilledWith.OntoLab:ChemicalAmount
$\top \sqsubseteq \forall$ OntoLab:isManagedBy.OntoAgent:Service
$\top \sqsubseteq \forall$ OntoLab:isPreparedBy.OntoLab:PreparationMethod
$\top \sqsubseteq \forall$ OntoLab:specifies.OntoLab:LabEquipment
$\top \sqsubseteq \forall$ OntoLab:wasGeneratedFor.OntoReaction:ReactionExperiment

Data properties:

$\exists$ OntoLab:containsUnidentifiedComponent.$\top \sqsubseteq$ OntoLab:ChemicalAmount
$\exists$ OntoLab:stateLastUpdatedAt.$\top \sqsubseteq$ saref:State
$\top \sqsubseteq \forall$ OntoLab:containsUnidentifiedComponent.xsd:boolean
$\top \sqsubseteq \forall$ OntoLab:stateLastUpdatedAt.xsd:decimal

A.1.11.4 OntoVapourtec

Classes:

OntoVapourtec:AutoSampler $\sqsubseteq$ OntoLab:LabEquipment
OntoVapourtec:AutoSamplerCommand $\sqsubseteq$ saref:Command
OntoVapourtec:AutoSamplerFunction $\sqsubseteq$ saref:Function
OntoVapourtec:AutoSamplerSite $\sqsubseteq \leq 1$ OntoVapourtec:holds.OntoLab:Vial
OntoVapourtec:AutoSamplerTask $\sqsubseteq$ saref:Task
OntoVapourtec:CleanReactor $\sqsubseteq$ OntoVapourtec:VapourtecFunction
OntoVapourtec:CleaningReaction $\sqsubseteq$ OntoVapourtec:VapourtecState
OntoVapourtec:ClearReactions $\sqsubseteq$ saref:Command
OntoVapourtec:ConnectToFlowCommander $\sqsubseteq$ saref:Command
OntoVapourtec:Connection $\sqsubseteq$ OntoVapourtec:VapourtecFunction
OntoVapourtec:DryRunState $\sqsubseteq$ OntoVapourtec:VapourtecState
OntoVapourtec:ExpFilePath $\sqsubseteq$ OntoLab:Argument
OntoVapourtec:FaultRecovery $\sqsubseteq$ OntoVapourtec:VapourtecFunction
OntoVapourtec:FaultRecoveryCommand $\sqsubseteq$ saref:Command
OntoVapourtec:Faulty $\sqsubseteq$ OntoVapourtec:VapourtecState
OntoVapourtec:FinalCleaning $\sqsubseteq$ OntoVapourtec:VapourtecState
OntoVapourtec:FlowChemistry $\sqsubseteq$ saref:Task
OntoVapourtec:FlowCommander $\sqsubseteq$ saref:Service
OntoVapourtec:FractionCollector $\sqsubseteq$ OntoVapourtec:CollectionMethod
OntoVapourtec:GetCommand $\sqsubseteq$ saref:Command
OntoVapourtec:GetState $\sqsubseteq$ OntoVapourtec:VapourtecFunction
OntoVapourtec:Idle $\sqsubseteq$ OntoVapourtec:VapourtecState
OntoVapourtec:Inactive $\sqsubseteq$ OntoVapourtec:VapourtecState
OntoVapourtec:Initialising $\sqsubseteq$ OntoVapourtec:VapourtecState
OntoVapourtec:Launch $\sqsubseteq$ OntoVapourtec:VapourtecFunction
OntoVapourtec:LoadExperiment $\sqsubseteq$ saref:Command
OntoVapourtec:Null $\sqsubseteq$ OntoVapourtec:VapourtecState
OntoVapourtec:PumpSettings $\sqsubseteq$ OntoLab:EquipmentSettings
OntoVapourtec:PumpSettings $\sqsubseteq \leq 1$ OntoVapourtec:hasFlowRateSetting.OntoLab:FlowRateSetting
OntoVapourtec:PumpSettings $\sqsubseteq \leq 1$ OntoVapourtec:hasSampleLoopVolumeSetting.OntoVapourtec:SampleLoopVolumeSetting
OntoVapourtec:PumpSettings $\sqsubseteq \leq 1$ OntoVapourtec:hasStoichiometryRatioSetting.OntoVapourtec:StoichiometryRatioSetting
OntoVapourtec:ReactionCompleted $\sqsubseteq$ OntoVapourtec:VapourtecState
OntoVapourtec:ReactorSettings $\sqsubseteq$ OntoLab:EquipmentSettings
OntoVapourtec:ReactorSettings $\sqsubseteq \leq 1$ OntoVapourtec:hasReactorTemperatureSetting.OntoVapourtec:ReactorTemperatureSetting
OntoVapourtec:ReactorSettings $\sqsubseteq \leq 1$ OntoVapourtec:hasResidenceTimeSetting.OntoVapourtec:ResidenceTimeSetting
OntoVapourtec:ReactorTemperatureSetting $\sqsubseteq$ OntoLab:TemperatureSetting
OntoVapourtec:ResidenceTimeSetting $\sqsubseteq$ OntoLab:DurationSetting
OntoVapourtec:RunReactor $\sqsubseteq$ OntoVapourtec:VapourtecFunction
OntoVapourtec:RunningReaction $\sqsubseteq$ OntoVapourtec:VapourtecState
OntoVapourtec:SampleLoopVolumeSetting $\sqsubseteq$ OntoLab:VolumeSetting
OntoVapourtec:SingleReceptacle $\sqsubseteq$ OntoVapourtec:CollectionMethod
OntoVapourtec:StartFlowCommander $\sqsubseteq$ saref:Command

OntoVapourtec:StoichiometryRatioSetting ⊑ OntoLab:ParameterSetting
OntoVapourtec:VapourtecFunction ⊑ saref:Function
OntoVapourtec:VapourtecR2Pump ⊑ OntoLab:LabEquipment
OntoVapourtec:VapourtecR2PumpCommand ⊑ saref:Command
OntoVapourtec:VapourtecR2PumpFunction ⊑ saref:Function
OntoVapourtec:VapourtecR2PumpTask ⊑ saref:Task
OntoVapourtec:VapourtecR4Reactor ⊑ OntoLab:LabEquipment
OntoVapourtec:VapourtecR4Reactor ⊑ = 1 OntoVapourtec:hasInternalDiameter.om:Diameter
OntoVapourtec:VapourtecR4Reactor ⊑ = 1 OntoVapourtec:hasReactorLength.om:Length
OntoVapourtec:VapourtecR4Reactor ⊑ = 1 OntoVapourtec:hasReactorTemperatureLowerLimit.om:CelsiusTemperature
OntoVapourtec:VapourtecR4Reactor ⊑ = 1 OntoVapourtec:hasReactorTemperatureUpperLimit.om:CelsiusTemperature
OntoVapourtec:VapourtecR4Reactor ⊑ = 1 OntoVapourtec:hasReactorVolume.om:Volume
OntoVapourtec:VapourtecR4ReactorCommand ⊑ saref:Command
OntoVapourtec:VapourtecR4ReactorFunction ⊑ saref:Function
OntoVapourtec:VapourtecR4ReactorTask ⊑ saref:Task
OntoVapourtec:VapourtecRS400 ⊑ OntoLab:LabEquipment
OntoVapourtec:VapourtecRS400 ⊑ = 1 OntoVapourtec:hasCollectionMethod.OntoVapourtec:CollectionMethod
OntoVapourtec:VapourtecRS400 ⊑ = 1 OntoVapourtec:recommendedReactionScale.om:Volume
OntoVapourtec:VapourtecState ⊑ saref:State

Object properties:
OntoVapourtec:hasFlowRateSetting ⊑ OntoLab:hasSetting
OntoVapourtec:hasReactorTemperatureSetting ⊑ OntoLab:hasSetting
OntoVapourtec:hasResidenceTimeSetting ⊑ OntoLab:hasSetting
OntoVapourtec:hasSampleLoopVolumeSetting ⊑ OntoLab:hasSetting
OntoVapourtec:hasStoichiometryRatioSetting ⊑ OntoLab:hasSetting
∃ OntoVapourtec:hasCollectionMethod.⊤ ⊑ OntoVapourtec:VapourtecRS400
∃ OntoVapourtec:hasFlowRateSetting.⊤ ⊑ OntoVapourtec:PumpSettings
∃ OntoVapourtec:hasInternalDiameter.⊤ ⊑ OntoVapourtec:VapourtecR4Reactor
∃ OntoVapourtec:hasReactorLength.⊤ ⊑ OntoVapourtec:VapourtecR4Reactor
∃ OntoVapourtec:hasReactorMaterial.⊤ ⊑ OntoVapourtec:VapourtecR4Reactor
∃ OntoVapourtec:hasReactorTemperatureLowerLimit.⊤ ⊑ OntoVapourtec:VapourtecR4Reactor
∃ OntoVapourtec:hasReactorTemperatureSetting.⊤ ⊑ OntoVapourtec:ReactorSettings
∃ OntoVapourtec:hasReactorTemperatureUpperLimit.⊤ ⊑ OntoVapourtec:VapourtecR4Reactor
∃ OntoVapourtec:hasReactorVolume.⊤ ⊑ OntoVapourtec:VapourtecR4Reactor
∃ OntoVapourtec:hasReagentSource.⊤ ⊑ OntoVapourtec:VapourtecR2Pump
∃ OntoVapourtec:hasResidenceTimeSetting.⊤ ⊑ OntoVapourtec:ReactorSettings
∃ OntoVapourtec:hasSampleLoopVolumeSetting.⊤ ⊑ OntoVapourtec:PumpSettings
∃ OntoVapourtec:hasSite.⊤ ⊑ OntoVapourtec:AutoSampler
∃ OntoVapourtec:hasStoichiometryRatioSetting.⊤ ⊑ OntoVapourtec:PumpSettings

∃ OntoVapourtec:hasVapourtecInputFile.⊤ ⊑ OntoReaction:ReactionExperiment
∃ OntoVapourtec:holds.⊤ ⊑ OntoVapourtec:AutoSamplerSite
∃ OntoVapourtec:pumpsLiquidFrom.⊤ ⊑ OntoVapourtec:PumpSettings
∃ OntoVapourtec:recommendedReactionScale.⊤ ⊑ OntoVapourtec:VapourtecRS400
∃ OntoVapourtec:sampleLoopVolume.⊤ ⊑ OntoVapourtec:AutoSampler
∃ OntoVapourtec:toReceptacle.⊤ ⊑ OntoVapourtec:SingleReceptacle
⊤ ⊑ ∀ OntoVapourtec:hasCollectionMethod.OntoVapourtec:CollectionMethod
⊤ ⊑ ∀ OntoVapourtec:hasFlowRateSetting.OntoLab:FlowRateSetting
⊤ ⊑ ∀ OntoVapourtec:hasInternalDiameter.om:Diameter
⊤ ⊑ ∀ OntoVapourtec:hasReactorLength.om:Length
⊤ ⊑ ∀ OntoVapourtec:hasReactorMaterial.OntoCAPE_Behavior:MaterialAmount
⊤ ⊑ ∀ OntoVapourtec:hasReactorTemperatureLowerLimit.om:CelsiusTemperature
⊤ ⊑ ∀ OntoVapourtec:hasReactorTemperatureSetting.OntoVapourtec:ReactorTemperatureSetting
⊤ ⊑ ∀ OntoVapourtec:hasReactorTemperatureUpperLimit.om:CelsiusTemperature
⊤ ⊑ ∀ OntoVapourtec:hasReactorVolume.om:Volume
⊤ ⊑ ∀ OntoVapourtec:hasReagentSource.OntoLab:ReagentBottle
⊤ ⊑ ∀ OntoVapourtec:hasResidenceTimeSetting.OntoVapourtec:ResidenceTimeSetting
⊤ ⊑ ∀ OntoVapourtec:hasSampleLoopVolumeSetting.OntoVapourtec:SampleLoopVolumeSetting
⊤ ⊑ ∀ OntoVapourtec:hasSite.OntoVapourtec:AutoSamplerSite
⊤ ⊑ ∀ OntoVapourtec:hasStoichiometryRatioSetting.OntoVapourtec:StoichiometryRatioSetting
⊤ ⊑ ∀ OntoVapourtec:hasVapourtecInputFile.OntoVapourtec:VapourtecInputFile
⊤ ⊑ ∀ OntoVapourtec:holds.OntoLab:Vial
⊤ ⊑ ∀ OntoVapourtec:pumpsLiquidFrom.(OntoLab:ReagentBottle ⊔ OntoVapourtec:AutoSamplerSite)
⊤ ⊑ ∀ OntoVapourtec:recommendedReactionScale.om:Volume
⊤ ⊑ ∀ OntoVapourtec:sampleLoopVolume.om:Volume
⊤ ⊑ ∀ OntoVapourtec:toReceptacle.OntoLab:WasteBottle

Data properties:
∃ OntoVapourtec:lastLocalModifiedAt.⊤ ⊑ OntoVapourtec:VapourtecInputFile
∃ OntoVapourtec:lastUploadedAt.⊤ ⊑ OntoVapourtec:VapourtecInputFile
∃ OntoVapourtec:localFilePath.⊤ ⊑ OntoVapourtec:VapourtecInputFile
∃ OntoVapourtec:locationID.⊤ ⊑ (OntoVapourtec:AutoSamplerSite ⊔ OntoVapourtec:VapourtecR2Pump ⊔ OntoVapourtec:VapourtecR4Reactor)
∃ OntoVapourtec:remoteFilePath.⊤ ⊑ OntoVapourtec:VapourtecInputFile
⊤ ⊑ ∀ OntoVapourtec:lastLocalModifiedAt.xsd:decimal
⊤ ⊑ ∀ OntoVapourtec:lastUploadedAt.xsd:decimal
⊤ ⊑ ∀ OntoVapourtec:localFilePath.xsd:string
⊤ ⊑ ∀ OntoVapourtec:locationID.xsd:string
⊤ ⊑ ∀ OntoVapourtec:remoteFilePath.xsd:anyURI

A.1.11.5 OntoHPLC

Classes:

OntoHPLC:ChromatogramMeasurement ⊑ saref:Function
OntoHPLC:ChromatogramMeasurementCommand ⊑ saref:Command
OntoHPLC:ChromatogramPoint ⊑ = 1 OntoHPLC:atRetentionTime.OntoHPLC:RetentionTime
OntoHPLC:ChromatogramPoint ⊑ = 1 OntoHPLC:hasPeakArea.OntoHPLC:PeakArea
OntoHPLC:HighPerformanceLiquidChromatography ⊑ OntoLab:LabEquipment
OntoHPLC:InternalStandard ⊑ OntoCAPE_Phase_System:PhaseComponent
OntoHPLC:LiquidChromatography ⊑ saref:Task
OntoHPLC:PeakArea ⊑ om:Quantity
OntoHPLC:ResponseFactor ⊑ om:QuantityOfDimensionOne
OntoHPLC:RetentionTime ⊑ om:Duration

Object properties:

∃ OntoHPLC:atRetentionTime.⊤ ⊑ OntoHPLC:ChromatogramPoint
∃ OntoHPLC:characterises.⊤ ⊑ OntoHPLC:HPLCJob
∃ OntoHPLC:generatedFor.⊤ ⊑ OntoHPLC:HPLCReport
∃ OntoHPLC:hasJob.⊤ ⊑ OntoHPLC:HighPerformanceLiquidChromatography
∃ OntoHPLC:hasPastReport.⊤ ⊑ OntoHPLC:HighPerformanceLiquidChromatography
∃ OntoHPLC:hasPeakArea.⊤ ⊑ OntoHPLC:ChromatogramPoint
∃ OntoHPLC:hasReport.⊤ ⊑ OntoHPLC:HPLCJob
∃ OntoHPLC:hasResponseFactor.⊤ ⊑ OntoHPLC:HPLCMethod
∃ OntoHPLC:hasRetentionTime.⊤ ⊑ OntoHPLC:HPLCMethod
∃ OntoHPLC:indicatesComponent.⊤ ⊑ OntoHPLC:ChromatogramPoint
∃ OntoHPLC:records.⊤ ⊑ OntoHPLC:HPLCReport
∃ OntoHPLC:refersToSpecies.⊤ ⊑ (OntoHPLC:ResponseFactor ⊔ OntoHPLC:RetentionTime)
∃ OntoHPLC:reportExtension.⊤ ⊑ OntoHPLC:HighPerformanceLiquidChromatography
∃ OntoHPLC:usesInternalStandard.⊤ ⊑ OntoHPLC:HPLCMethod
∃ OntoHPLC:usesMethod.⊤ ⊑ OntoHPLC:HPLCJob
⊤ ⊑ ∀ OntoHPLC:atRetentionTime.OntoHPLC:RetentionTime
⊤ ⊑ ∀ OntoHPLC:characterises.OntoReaction:ReactionExperiment
⊤ ⊑ ∀ OntoHPLC:generatedFor.OntoLab:ChemicalAmount
⊤ ⊑ ∀ OntoHPLC:hasJob.OntoHPLC:HPLCJob
⊤ ⊑ ∀ OntoHPLC:hasPastReport.OntoHPLC:HPLCReport
⊤ ⊑ ∀ OntoHPLC:hasPeakArea.OntoHPLC:PeakArea
⊤ ⊑ ∀ OntoHPLC:hasReport.OntoHPLC:HPLCReport
⊤ ⊑ ∀ OntoHPLC:hasResponseFactor.OntoHPLC:ResponseFactor
⊤ ⊑ ∀ OntoHPLC:hasRetentionTime.OntoHPLC:RetentionTime
⊤ ⊑ ∀ OntoHPLC:indicatesComponent.OntoCAPE_Phase_System:PhaseComponent
⊤ ⊑ ∀ OntoHPLC:records.OntoHPLC:ChromatogramPoint
⊤ ⊑ ∀ OntoHPLC:refersToSpecies.OntoSpecies:Species
⊤ ⊑ ∀ OntoHPLC:reportExtension.yago:WikicatFilenameExtensions
⊤ ⊑ ∀ OntoHPLC:usesInternalStandard.OntoHPLC:InternalStandard
⊤ ⊑ ∀ OntoHPLC:usesMethod.OntoHPLC:HPLCMethod

Data properties:
$\exists$ OntoHPLC:lastLocalModifiedAt.$\top \sqsubseteq$ OntoHPLC:HPLCReport
$\exists$ OntoHPLC:lastUploadedAt.$\top \sqsubseteq$ OntoHPLC:HPLCReport
$\exists$ OntoHPLC:localFilePath.$\top \sqsubseteq$ OntoHPLC:HPLCMethod
$\exists$ OntoHPLC:localFilePath.$\top \sqsubseteq$ OntoHPLC:HPLCReport
$\exists$ OntoHPLC:localReportDirectory.$\top \sqsubseteq$ OntoHPLC:HighPerformanceLiquidChromatography
$\exists$ OntoHPLC:remoteFilePath.$\top \sqsubseteq$ OntoHPLC:HPLCMethod
$\exists$ OntoHPLC:remoteFilePath.$\top \sqsubseteq$ OntoHPLC:HPLCReport
$\exists$ OntoHPLC:retentionTimeMatchThreshold.$\top \sqsubseteq$ OntoHPLC:HPLCMethod
$\exists$ OntoHPLC:unidentified.$\top \sqsubseteq$ OntoHPLC:ChromatogramPoint
$\top \sqsubseteq \forall$ OntoHPLC:lastLocalModifiedAt.xsd:decimal
$\top \sqsubseteq \forall$ OntoHPLC:lastUploadedAt.xsd:decimal
$\top \sqsubseteq \forall$ OntoHPLC:localFilePath.xsd:string
$\top \sqsubseteq \forall$ OntoHPLC:localReportDirectory.xsd:string
$\top \sqsubseteq \forall$ OntoHPLC:remoteFilePath.xsd:anyURI
$\top \sqsubseteq \forall$ OntoHPLC:retentionTimeMatchThreshold.xsd:float
$\top \sqsubseteq \forall$ OntoHPLC:unidentified.xsd:boolean

A.1.11.6 OntoGoal

Classes:
OntoGoal:DesignOfExperiment $\sqsubseteq$ OntoGoal:Step
OntoGoal:DesignOfExperiment $\sqsubseteq \forall$ OntoGoal:hasNextStep.OntoGoal:RxnExpExecution
OntoGoal:Goal $\sqsubseteq \geq 1$ OntoGoal:hasPlan.OntoGoal:Plan
OntoGoal:Goal $\sqsubseteq \leq 1$ OntoGoal:desiresGreaterThan.om:Quantity
OntoGoal:Goal $\sqsubseteq \leq 1$ OntoGoal:desiresLessThan.om:Quantity
OntoGoal:GoalSet $\sqsubseteq \geq 1$ OntoGoal:hasGoal.OntoGoal:Goal
OntoGoal:GoalSet $\sqsubseteq = 1$ OntoGoal:hasRestriction.OntoGoal:Restriction
OntoGoal:Plan $\sqsubseteq \geq 1$ OntoGoal:hasStep.OntoGoal:Step
OntoGoal:PostProcessing $\sqsubseteq$ OntoGoal:Step
OntoGoal:Result $\sqsubseteq = 1$ OntoGoal:refersTo.om:Quantity
OntoGoal:RxnExpExecution $\sqsubseteq$ OntoGoal:Step
OntoGoal:RxnExpExecution $\sqsubseteq \forall$ OntoGoal:hasNextStep.OntoGoal:PostProcessing
OntoGoal:RxnOptPlan $\sqsubseteq$ OntoGoal:Plan
OntoGoal:Step $\sqsubseteq \geq 1$ OntoGoal:canBePerformedBy.OntoAgent:Service

Object properties:
OntoGoal:desiresGreaterThan $\sqsubseteq$ OntoGoal:desires
OntoGoal:desiresLessThan $\sqsubseteq$ OntoGoal:desires
$\exists$ OntoGoal:canBePerformedBy.$\top \sqsubseteq$ OntoGoal:Step
$\exists$ OntoGoal:desires.$\top \sqsubseteq$ OntoGoal:Goal
$\exists$ OntoGoal:desiresGreaterThan.$\top \sqsubseteq$ OntoGoal:Goal
$\exists$ OntoGoal:desiresLessThan.$\top \sqsubseteq$ OntoGoal:Goal
$\exists$ OntoGoal:hasGoal.$\top \sqsubseteq$ OntoGoal:GoalSet
$\exists$ OntoGoal:hasNextStep.$\top \sqsubseteq$ OntoGoal:Step

$\exists$ OntoGoal:hasPlan.$\top$ $\sqsubseteq$ OntoGoal:Goal
$\exists$ OntoGoal:hasRestriction.$\top$ $\sqsubseteq$ OntoGoal:GoalSet
$\exists$ OntoGoal:hasResult.$\top$ $\sqsubseteq$ OntoGoal:Goal
$\exists$ OntoGoal:hasStep.$\top$ $\sqsubseteq$ OntoGoal:Plan
$\exists$ OntoGoal:hasStep.$\top$ $\sqsubseteq$ OntoGoal:RxnOptPlan
$\exists$ OntoGoal:refersTo.$\top$ $\sqsubseteq$ OntoGoal:Result
$\top \sqsubseteq \forall$ OntoGoal:canBePerformedBy.OntoAgent:Service
$\top \sqsubseteq \forall$ OntoGoal:desires.om:Quantity
$\top \sqsubseteq \forall$ OntoGoal:desiresGreaterThan.om:Quantity
$\top \sqsubseteq \forall$ OntoGoal:desiresLessThan.om:Quantity
$\top \sqsubseteq \forall$ OntoGoal:hasGoal.OntoGoal:Goal
$\top \sqsubseteq \forall$ OntoGoal:hasNextStep.OntoGoal:Step
$\top \sqsubseteq \forall$ OntoGoal:hasPlan.OntoGoal:Plan
$\top \sqsubseteq \forall$ OntoGoal:hasRestriction.OntoGoal:Restriction
$\top \sqsubseteq \forall$ OntoGoal:hasResult.om:Quantity
$\top \sqsubseteq \forall$ OntoGoal:hasStep.OntoGoal:Step
$\top \sqsubseteq \forall$ OntoGoal:hasStep.(OntoGoal:DesignOfExperiment $\sqcup$ OntoGoal:PostProcessing $\sqcup$ OntoGoal:RxnExpExecution)
$\top \sqsubseteq \forall$ OntoGoal:refersTo.om:Quantity

Data properties:

$\exists$ OntoGoal:cycleAllowance.$\top$ $\sqsubseteq$ OntoGoal:Restriction
$\exists$ OntoGoal:deadline.$\top$ $\sqsubseteq$ OntoGoal:Restriction
$\top \sqsubseteq \forall$ OntoGoal:cycleAllowance.xsd:int
$\top \sqsubseteq \forall$ OntoGoal:deadline.xsd:string

A.2 Agent framework

This section describes the agents developed in this work. Firstly, we provide a brief overview of the derived information framework and explain how it was adopted in this study. We then delve into the internal logic of each of the agents involved and illustrate their unified modelling language (UML) activity diagram. We also present their distributed deployment that is networked via the knowledge graph. The section concludes by showcasing the derived information stepping process that snapshots different stages in the workflow.

A.2.1 Derived information framework

This study employs the asynchronous mode of the derived information framework [19] as a workflow management system, where each component in the workflow is realised as a 'derivation agent', a job request is named as a 'derivation', and the data flow is considered as 'derived information'. The framework manages agents' communication with the knowledge graph when retrieving inputs and populating outputs. The framework also standardises the deployment process using containers with environment variables to configure specific agent settings. Developers only need to provide agent logic that transforms inputs into outputs. After deployment, agents register themselves in the knowledge graph and operate autonomously to handle job requests and pass on information. More technical details can be found in the Python version of the framework, *i.e.* `pyderivationagent` package [24].

A.2.2 DoE Agent

Supplementary Figure S8 provides a UML activity diagram of the Design of Experiments (DoE) Agent (v1.2.0), which utilises `OntoDoE:DesignOfExperiment` to configure the metadata of DoE studies and `OntoLab:Laboratory` to locate the lab where the experiment is expected to be carried out. The relevant information is queried from the knowledge graph and used to form Python objects required by the `summit` package [17]. In this iteration, the TSEMO [25] algorithm is used to propose the next experiment condition. The suggestions are then parsed to construct a Python object of `OntoReaction:ReactionExperiment` and translated to triples. Notably, the possible `OntoReaction:InputChemical` is located based on the chemicals present in the chemical containers available in the specified laboratory. The derived information framework populates these statements back into the knowledge graph and connects the IRI of the created experiment as input to the next steps in the workflow. Integration tests are provided in folder `DoEAgent/doeagent/tests` that simulate the DoE studies, accounting for situations both with and without prior experiments.

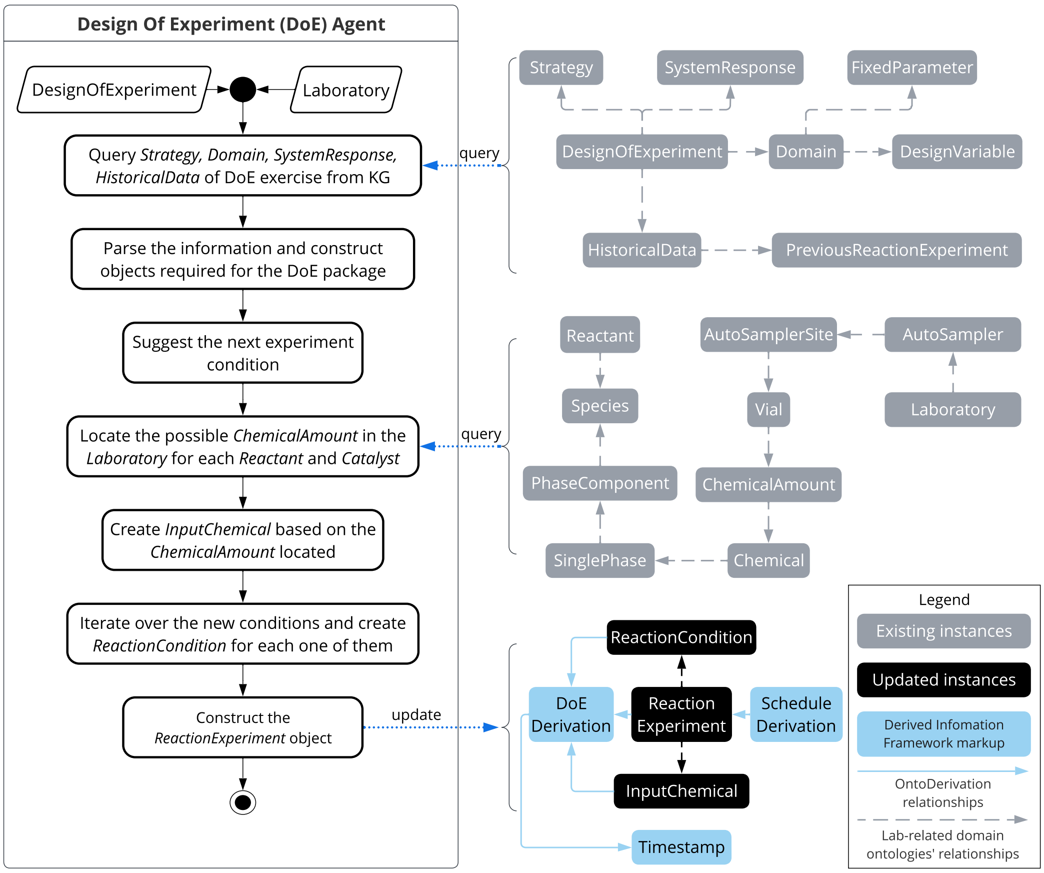

Supplementary Figure S8: *UML activity diagram of Design of Experiment (DoE) Agent. The blue arrows denote the data query and update between objects held in memory by the agent and instances in the knowledge graph.*

A.2.3 VapourtecSchedule Agent

Supplementary Figure S9 illustrates the scheduling process of experiments by Vapourtec Schedule Agent (v1.2.0) to support collaboration between multiple laboratories located in different geographical areas. The experiment to be carried out and the target laboratory for experimentation are defined by the input instances of `OntoReaction:ReactionExperiment` and `OntoLab:Laboratory`, respectively. The scheduler uses the First In First Out (FIFO) algorithm, also known as First Come First Serve (FCFS), for assigning the reaction experiment to the digital twin of a suitable reactor that has the required chemicals and is capable of conducting the reaction conditions. This is done by creating the job requests (‘derivation’) for the agents responsible for managing the hardware. The scheduler agent monitors the progress of the experiment by periodically checking if the new HPLC report is generated. The new report is then passed on to the post-processing step as input. More sophisticated scheduling algorithms may be implemented in future versions. Integration tests are provided in folder `VapourtecScheduleAgent/vapourtecscheduleagent/tests` that simulate the experiment scheduling process with dummy equipment digital twins.

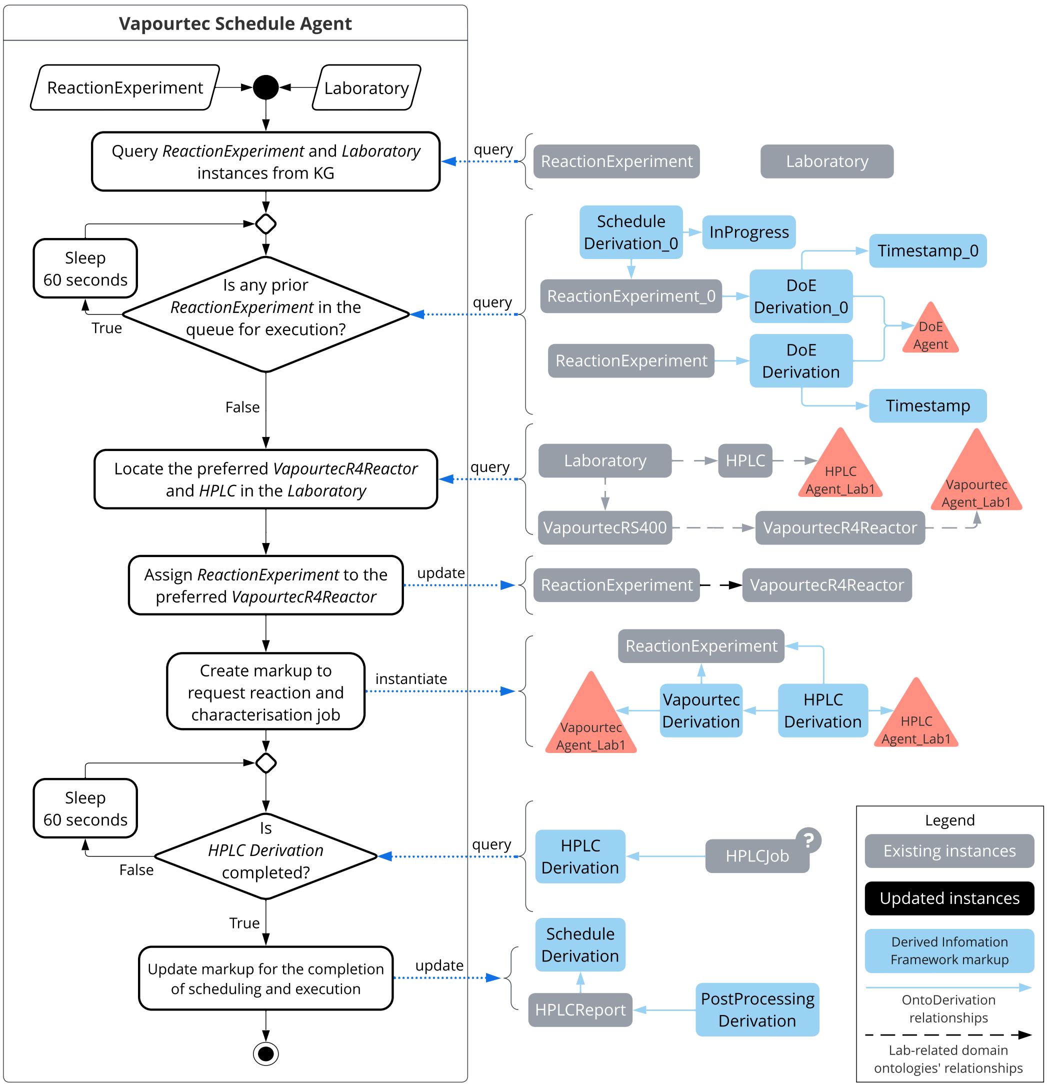

Supplementary Figure S9: *UML activity diagram of Vapourtec Schedule Agent. The blue arrows denote the data query and update between objects held in memory by the agent and instances in the knowledge graph. The question mark refers to the process of querying if the instance is available in the knowledge graph.*

A.2.4 Vapourtec Agent

Supplementary Figure S10 presents the internal logic of the Vapourtec Agent (v1.2.0), which liaises between the physical setup and its digital counterpart by surrounding the software provided by the hardware vendor, *i.e.* `FlowCommander` (v1.12). The primary usage of `FlowCommander` is for manual control and inspection, therefore, only limited high-level functions are provided by its API.

The only data that can be programmatically retrieved from the hardware is the state of the entire system. These states indicate the stage of the reaction execution, *e.g.* initialising, running reaction, cleaning, *etc.*, and are updated in the knowledge graph every 30 seconds. The live data collected from the temperature and pressure sensors are only displayed in the graphical interface of `FlowCommander`.

Upon an assigned `OntoReaction:ReactionExperiment`, the Vapourtec Agent translates the reaction conditions to a list of equipment settings to configure the Vapourtec equipment it manages. This enables tailored pump settings when locating the inlet streams that match with the concentrations of `OntoReaction:InputChemical` as we use different sourcing methods in the two labs, *i.e.* autosampler in Cambridge and reagent bottles in Singapore. Once all configurations are prepared, the agent compiles a CSV file and saves a copy in the file server for the record. The command for execution is sent when the hardware is idle to ensure no interruption to the cleaning steps of the previous experiment.

During the reaction, the agent updates the liquid level of the chemical containers in the digital twin. Together with the labelled warning level, this allows the agent to only select the vials that still have enough chemicals in the following reactions, *i.e.* those above the warning level, as well as inform researchers if any containers need a refill.

As the reactor is physically tubed to the HPLC via a four-way VICI switching valve, `FlowCommander` is configured with an external analysis trigger to time the sample injection. The material flow for the reaction and characterisation will run in the physical world without any further intervention required. The agent then creates an instance of `OntoLab:ChemicalAmount` referring to the reactor outlet. Populating its triples to the knowledge graph and assigning its IRI as the input to the HPLC Agent are again managed by the derived information framework.

Integration tests are provided in folder `VapourtecAgent/vapourtecagent/tests`, including the generation of Vapourtec job files, equipment configuration translation, liquid level calculations, and hardware status monitoring.

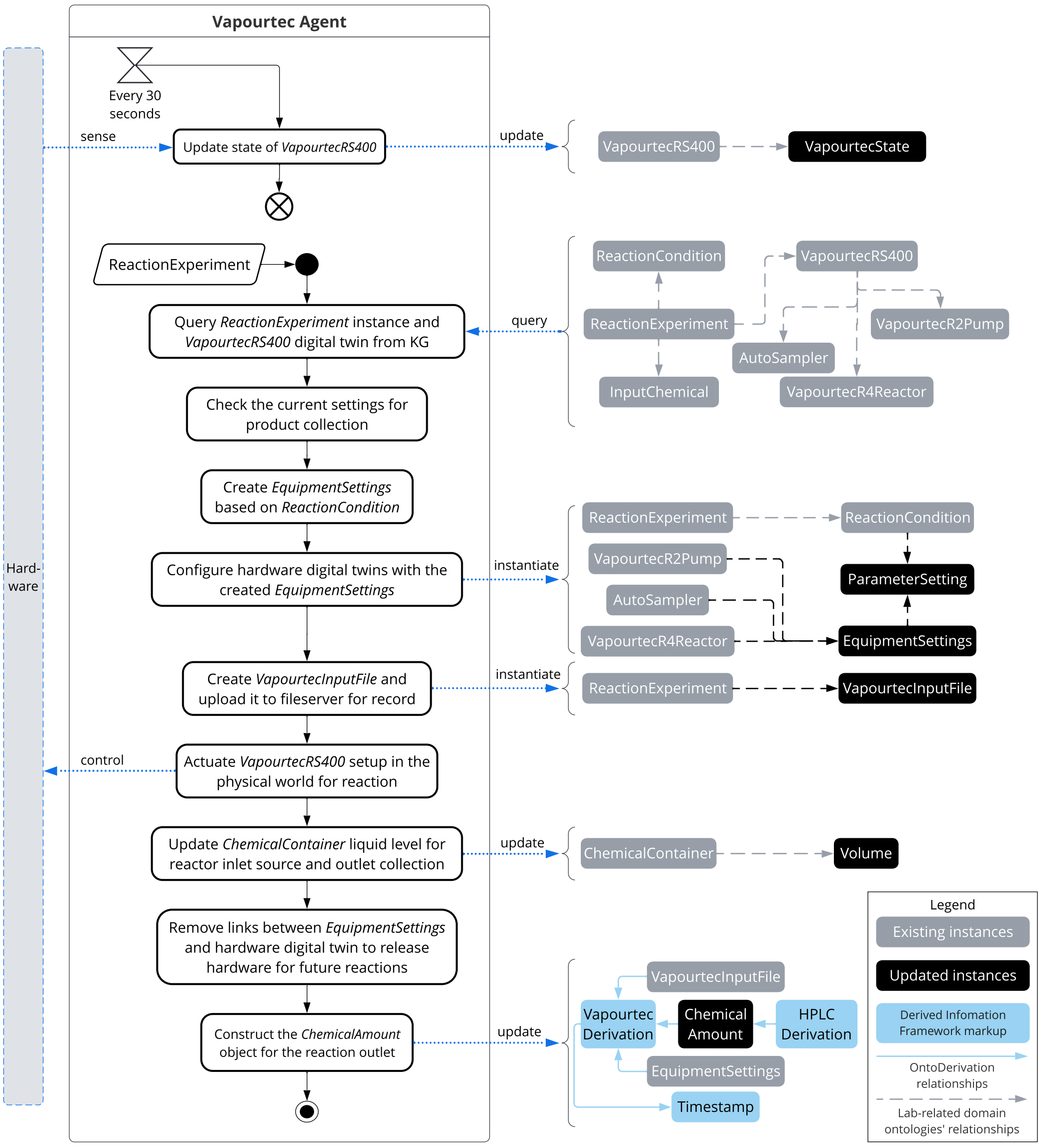

Supplementary Figure S10: *UML activity diagram of Vapourtec Agent. The blue arrows on the left-hand side refer to commands and data exchanged between the agent and the hardware to the agent, whereas those on the right-hand side denote the data query and update between objects held in memory of the agent and instances in the knowledge graph.*

A.2.5 HPLC Agent

Supplementary Figure S11 illustrates the functionalities of the HPLC Agent (v1.2.0), which is responsible for monitoring HPLC reports and uploading newly generated reports to the knowledge graph. The purpose of this agent is not to provide complete control of HPLC analysis, as it is physically triggered by the injection from the four-way VICI switching valve. We refer interested readers to Nambiar et al. [26] for codes on a more comprehensive control of HPLC.

The connection between the newly generated report and the reaction experiment it characterises is initiated by the job request once the instance of `OntoLab:ChemicalAmount` is instantiated by the Vapourtec Agent. If a new HPLC report is generated after the analysis has commenced, a new instance of `OntoHPLC:HPLCJob` is constructed and populated back to link the relevant instances.

Integration tests are provided in folder `HPLCAgent/hplcagent/tests`, including monitoring local report generation when new HPLC jobs are initiated, uploading local reports to a remote file server, and the registration of agents with the digital twin they oversee.

A.2.6 HPLCPostPro Agent

Supplementary Figure S12 depicts the procedures involved in post-processing `OntoHPLC:HPLCReport` by HPLCPostPro Agent (v1.2.0) that utilises a hypothetical model of the reactor and its inlet/outlet streams. Firstly, the agent constructs the reactor model and its inlet streams. It then identifies the chemical species from the raw HPLC report and calculates their concentrations to form the reactor outlet. The performance indicators currently available include yield, conversion, run material cost, space-time yield (STY), and environmental factor (E-factor). Integration tests are provided in folder `HPLCPostProAgent/hplcpostproagent/tests`. These tests simulate various scenarios involving the processing of different raw HPLC reports, such as handling missing product peaks, missing side-product peaks, and the presence of unidentified peaks.

Supplementary Figure S13 provides the abstraction adopted in the hypothetical models used for post-processing. The data stored in the ontological objects is transferred to these models and employed to compute the performance indicators. The class of `DimensionalQuantity` is employed to support automated unit conversion when writing the calculated objectives back to the knowledge graph.

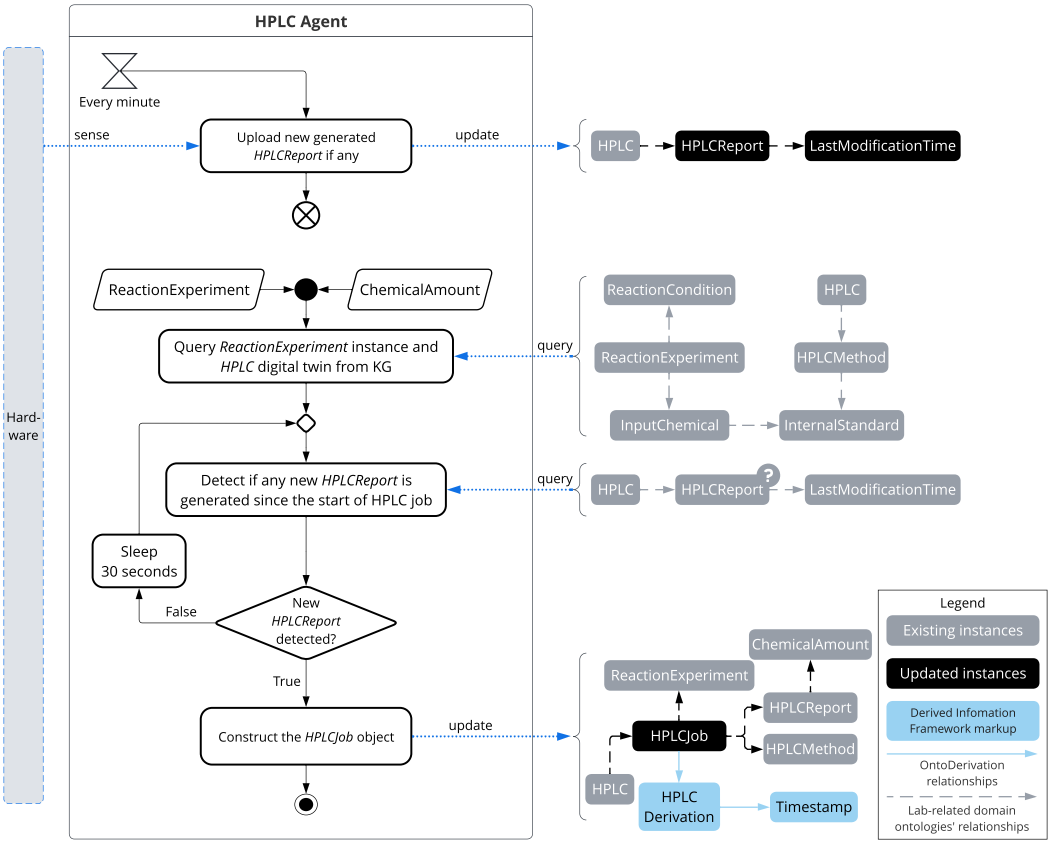

Supplementary Figure S11: *UML activity diagram of HPLC Agent. The blue arrow on the left-hand side refers to data transmitted from the hardware to the agent, whereas those on the right-hand side denote the data query and update between objects held in memory of the agent and instances in the knowledge graph. The question mark refers to the process of querying if the instance is available in the knowledge graph.*

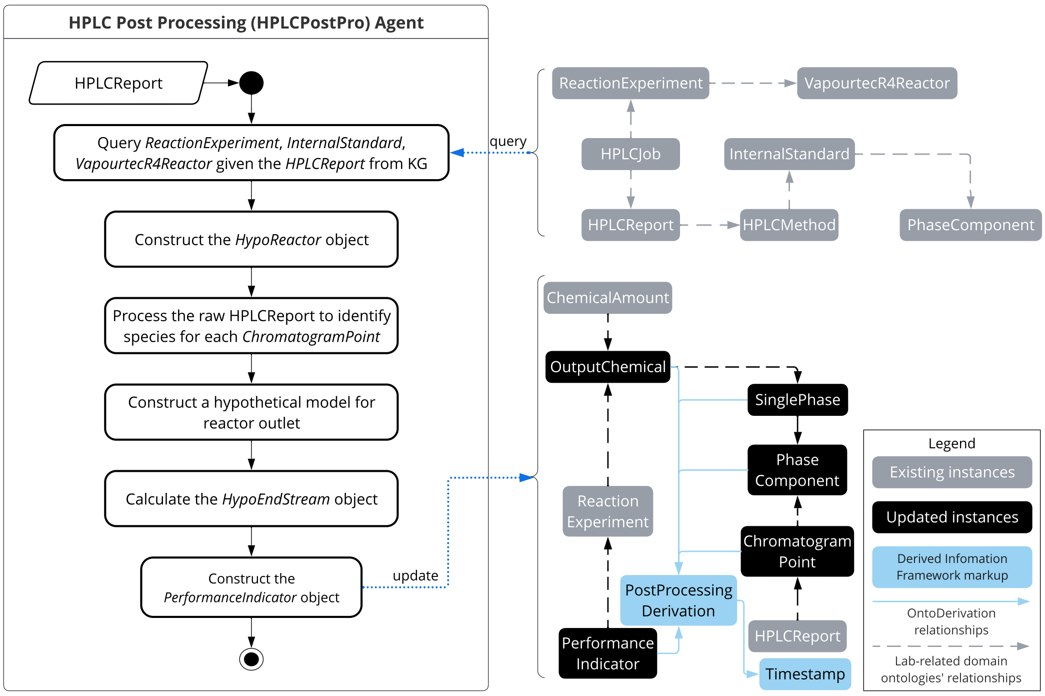

Supplementary Figure S12: *UML activity diagram of HPLC Post Processing (HPLCPost-Pro) Agent. The blue arrows denote the data query and update between objects held in memory by the agent and instances in the knowledge graph.*

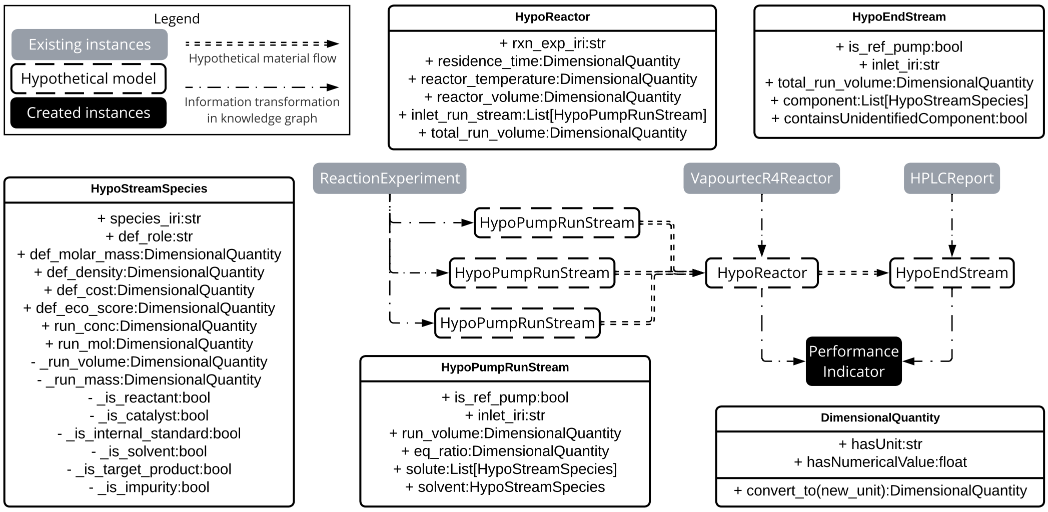

Supplementary Figure S13: *Hypothetical models for post-processing.*

The logic of the species identification from the raw HPLC report follows below steps:

1. Iterate through the list of peaks and remove any peaks that are outside the threshold range of available retention time for all species, labelling these peaks as unidentified.
2. For the remaining peaks that fall within the retention time threshold range, identify the peak with the largest area for each species and mark any unselected peaks as unidentified.
3. Calculate the concentration of each identified species by using the response factor and the corresponding peak area.

With the concentrations identified in the reaction end stream, the objectives can be calculated following the below equations:

$$\text{Yield} = \frac{\text{Actual } n_{\text{product}}}{\text{Theoretical } n_{\text{product}}} \times 100\% = \frac{\text{Actual } c_{\text{product}}}{\text{Theoretical } c_{\text{product}}} \times 100\%, \tag{S1}$$

where n_{product} refers to the number of moles of product produced and c_{product} refers to its concentration in the reaction stream. It should be noted that the theoretical amount that could be produced is calculated based on the limiting reactant.

$$\text{Conversion} = \frac{\text{Initial } n_{\text{limiting reactant}} - \text{Final } n_{\text{limiting reactant}}}{\text{Initial } n_{\text{limiting reactant}}} \times 100\%, \tag{S2}$$

where $n_{\text{limiting reactant}}$ is the amount of limiting reactant presented in the reaction mixture.

$$\text{Run Material Cost} = \frac{\sum_{i=1}^{k_{chemicals}} C_i V_i}{V_{\text{reaction scale}}}, \tag{S3}$$

where C_i is the cost of chemical i sourced from the pumps, V_i is the volume of chemical i used in the reaction, $k_{\text{chemicals}}$ is the number of chemicals sourced from the pumps (excluding the internal standard), and $V_{\text{reaction scale}}$ is the reaction scale for the pump that contains primary starting material, *i.e.* benzaldehyde.

$$\text{STY} = \frac{m_{\text{product}}}{V_{\text{reactor}} \times t_{\text{res}}}, \tag{S4}$$

where m_{product} is the mass of the product, V_{reactor} is the reactor volume, and t_{res} is the residence time.

$$\text{E-factor} = \frac{m_{\text{waste}}}{m_{\text{product}}}, \tag{S5}$$

where m_{waste} is the mass of waste generated and m_{product} is the mass of product produced. Note that if no product is produced, the E-factor will be assigned an infinity value.

A.2.7 ROGI Agent

Supplementary Figure S14 displays the functions of the Reaction Optimisation Goal Iteration (ROGI) Agent (v1.2.0), which oversees each DMTA cycle involved in the iterative pursuit of research goals.

In each iteration of the closed-loop optimisation process, the ROGI Agent initiates by creating an instance of `OntoDoE:DesignOfExperiment` by converting the specified goals in the `OntoGoal:GoalSet` into design objectives and filtering the prior results of `OntoReaction:ReactionExperiment` to compile the object of historical data. It then generates a sequence of job requests that consists of designing, scheduling, and post-processing a new experiment. As the workflow progresses, the knowledge graph automatically evolves. The ROGI Agent periodically monitors whether the objectives are computed and marks them as `OntoGoal:Result`, which is used to determine whether to proceed to the next round of optimisation.

It is worth noting that an instance of `Goal Iteration Derivation` is created per instance of `OntoLab:Laboratory` involved in the optimisation campaign. This design enables each SDL to function independently, but they can collaborate in case multiple SDLs are participating.

Integration tests are provided in folder `RxnOptGoalIterAgent/rxnoptgoaliteragent/tests`. These tests simulate a single DMTA cycle in two scenarios: one with prior experimental data and one without.

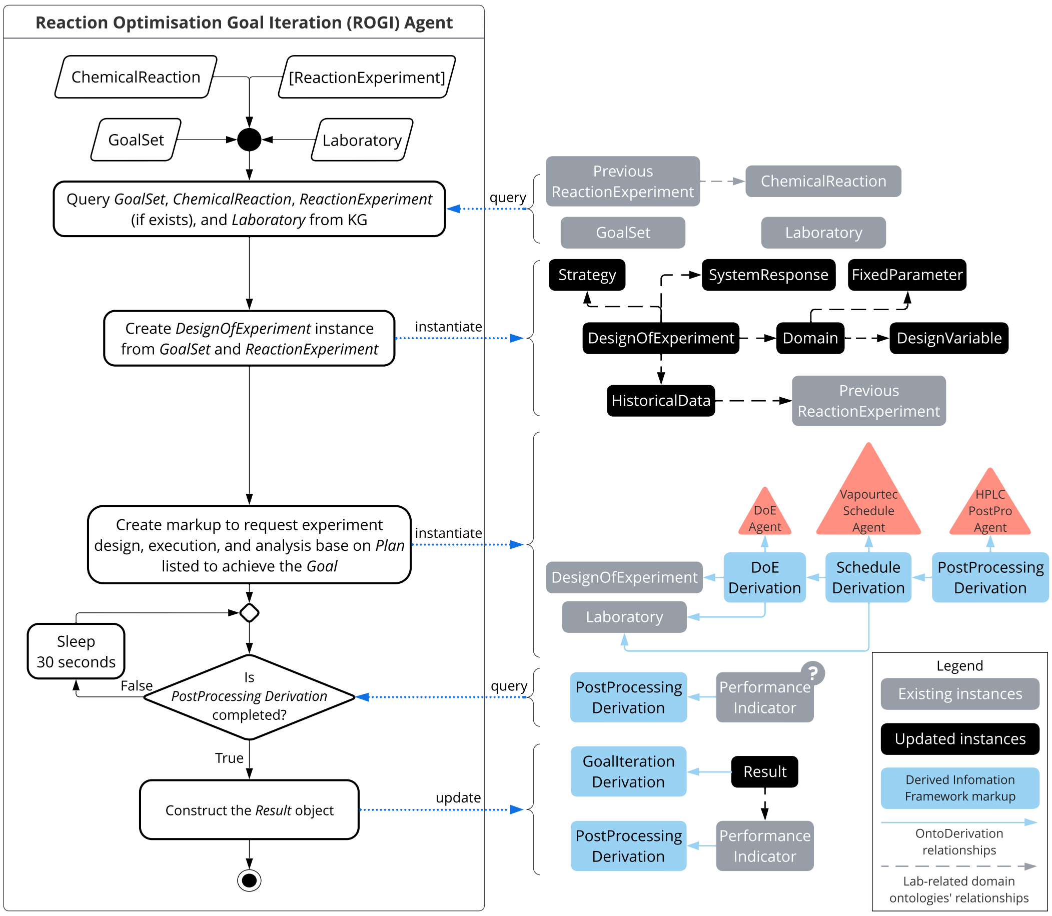

Supplementary Figure S14: *UML activity diagram of Reaction Optimisation Goal Iteration (ROGI) Agent. The blue arrows denote the data query and update between objects held in memory by the agent and instances in the knowledge graph. The question mark refers to the process of querying if the instance is available in the knowledge graph.*

A.2.8 ROG Agent

BDI agent architecture and consequently multi-agent system is well studied in the literature and its development has been surveyed in [27, 28]. There are different types of goals: test goals, achievement goals, and maintenance goals. In this study, the reaction optimisation problem can be seen as setting targets for achievement goals. Each step made by the agent towards achieving the goal can be seen as agents making rational decisions based on the status of its environment, *i.e.* the knowledge graph. As the system interacts with the physical world, it is also important to set resources and deadlines for goals. In the current development, these settings are provided by human researchers as a goal request from a web front end and subsequently handled by Reaction Optimisation Goal (ROG) Agent (v1.0.0).

Supplementary Figure S14 captures the two most important functionalities of the ROG Agent, *i.e.* handling the goal request (Supplementary Fig. S14(a)) and monitoring the subsequent iteration progress (Supplementary Fig. S14(b)). Upon receiving the goal request from the researchers, the ROG Agent first validates all request parameters and then translates them to actionable ontological representations based on concepts defined in OntoGoal, *e.g.* instance of `OntoGoal:GoalSet`. It then queries the knowledge graph to identify historical reaction experiments performed for the chemical reaction specified in the goal request. Should the current best-performing reaction experiment fail to meet the goal, the ROG Agent instantiates a `Goal Iteration Derivation` per `OntoLab:Laboratory` which will later be picked up by the ROGI Agent to orchestrate the actual workflow of the reaction experiment. The ROG Agent also adds a periodical job to monitor the currently active goal set to determine if one iteration is finished. If the goals have not been achieved and the resources are still available, the ROG Agent shares the latest results with all ongoing `Goal Iteration Derivation` instances. A request will then be sent to those finished derivations to trigger the next round of reaction. This ensures minimum downtime of the equipment and enables data sharing among all the involved laboratories.

Throughout the iterative process, the ROG Agent notifies developers about the latest progress. Supplementary Figure S15 shows an example email sent to the developer when it enters the next iteration of the goal pursuit.

Unit and integration tests are provided in folder `RxnOptGoalAgent/tests`. These tests can be categorised into two groups: *in silico* and physical. The *in silico* tests encompass various scenarios with increasing complexity, such as:

- Validity of all triples in the dummy digital lab.
- The ROG Agent creating the ROGI derivation and adding a periodic job for monitoring DMTA progress upon receiving a goal request.
- The ROGI Agent orchestrating other agents to perform one iteration of DMTA based on the derivation markup (excluding the ROG Agent).
- All agents running one iteration upon a goal request handled by the ROG Agent.
- All agents running multiple iterations upon a goal request until resources are exhausted.
- A collaborative optimisation involving two SDLs.

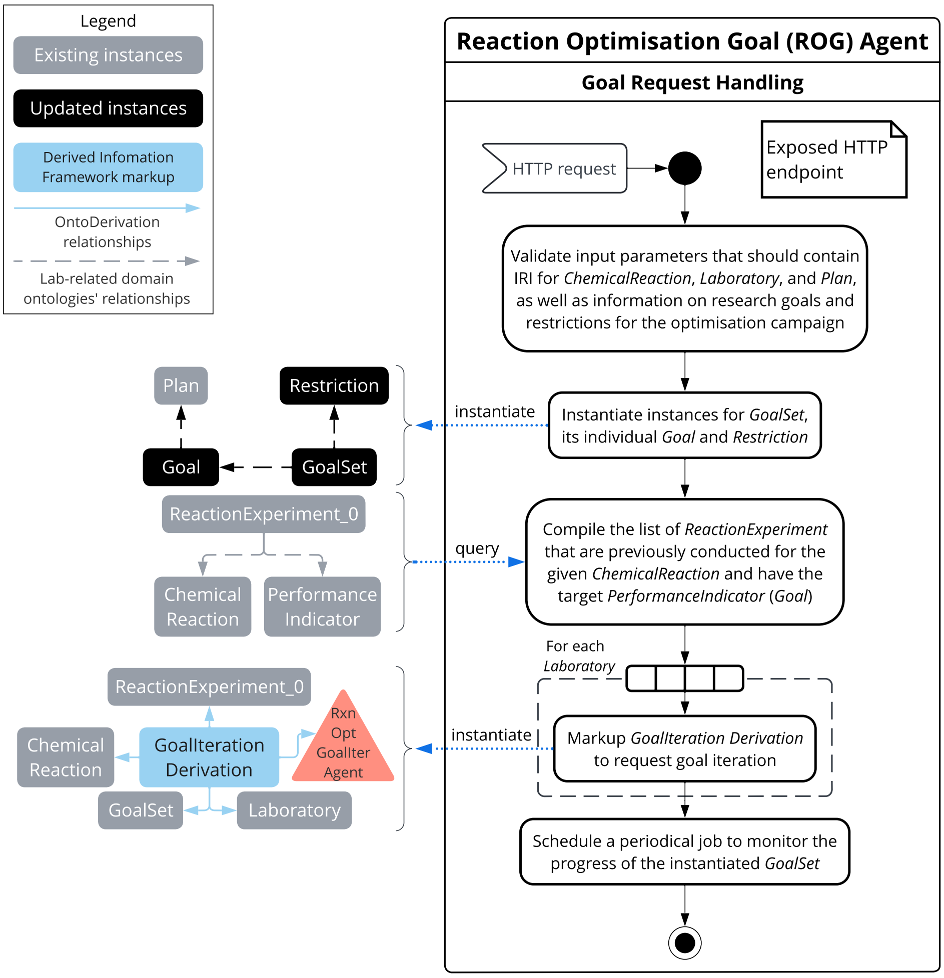

(a) The goal request handling occurs upon an HTTP request is issued from a web front end.

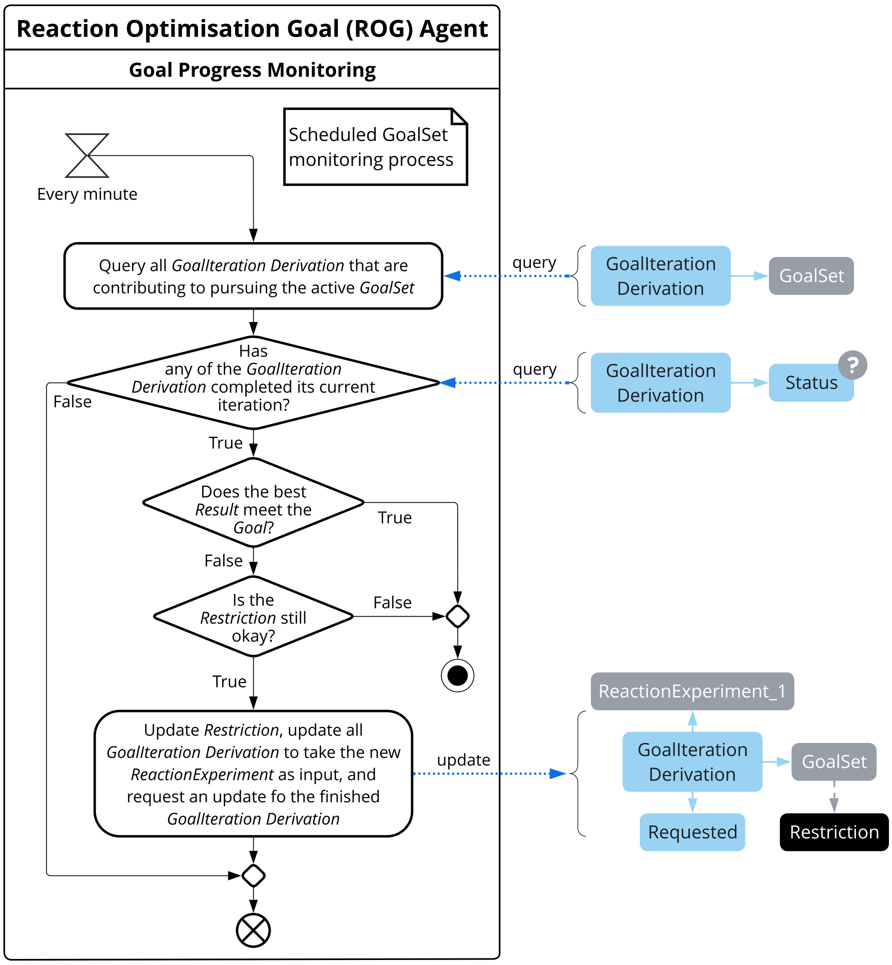

(b) The goal progress monitoring occurs as a periodical job following the processing of a goal request.

Supplementary Figure S14: *UML activity diagram of Reaction Optimisation Goal (ROG) Agent. The blue arrows denote the data query and update between objects held in memory by the agent and instances in the knowledge graph. The question mark refers to the process of querying if the instance is available in the knowledge graph.*

The physical test evaluates the DMTA cycles upon a goal request to the ROG Agent using real chemicals and resource constraints for a single experiment.

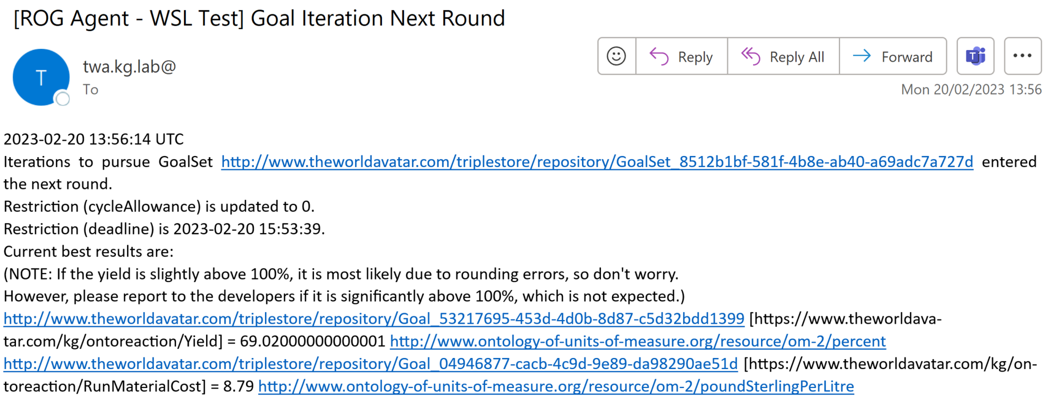

[ROG Agent - WSL Test] Goal Iteration Next Round

twa.kg.lab@

To

Reply | Reply All | Forward

Mon 20/02/2023 13:56

2023-02-20 13:56:14 UTC
Iterations to pursue GoalSet http://www.theworldavatar.com/triplestore/repository/GoalSet_8512b1bf-581f-4b8e-ab40-a69adc7a727d entered the next round.
Restriction (cycleAllowance) is updated to 0.
Restriction (deadline) is 2023-02-20 15:53:39.
Current best results are:
(NOTE: If the yield is slightly above 100%, it is most likely due to rounding errors, so don't worry.
However, please report to the developers if it is significantly above 100%, which is not expected.)
http://www.theworldavatar.com/triplestore/repository/Goal_53217695-453d-4d0b-8d87-c5d32bdd1399 [https://www.theworldavatar.com/kg/ontoreaction/Yield] = 69.02000000000001 http://www.ontology-of-units-of-measure.org/resource/om-2/percent
http://www.theworldavatar.com/triplestore/repository/Goal_04946877-cacb-4c9d-9e89-da98290ae51d [https://www.theworldavatar.com/kg/ontoreaction/RunMaterialCost] = 8.79 http://www.ontology-of-units-of-measure.org/resource/om-2/poundSterlingPerLitre

Supplementary Figure S15: *Example email notification for the progress of goal iteration.*

A.2.9 Distributed deployment

Supplementary Figure S16 presents the standard practice of the knowledge graph deployment adopted in the World Avatar. Being a decentralised system by design, the deployment of the agents in the World Avatar is distributed over the internet and networked via the knowledge graph. The public-facing part of the knowledge graph is often located at an internet-accessible location, whereas confidential data and agents are deployed within a local area network (LAN). The agents are developed to align with the modularised design that is normally referred to as low-coupling and high-cohesion in the microservices architecture.

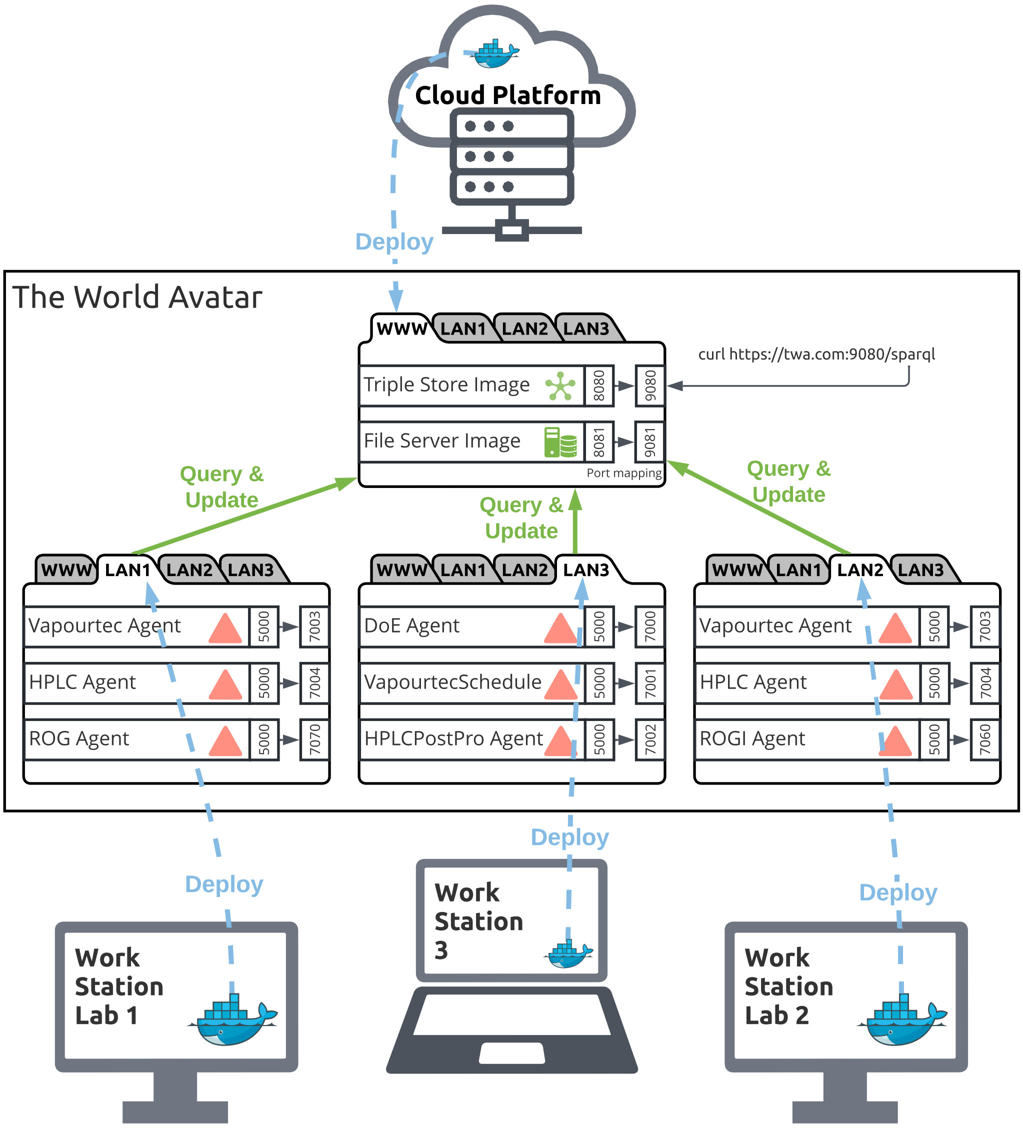

Supplementary Figure S16: *Schematic of the distributed deployment philosophy adopted in the World Avatar. The illustration of docker was created using flickr.com.*

In this distributed lab project, the triplestore and file server are hosted on a cloud platform with password-protected endpoints. The agents managing the hardware, *i.e.* Vapourtec and

HPLC Agent, are locally deployed in each lab, while the remaining agents are deployed across labs and a third location to demonstrate the decentralised design. All agents need an internet connection to access the knowledge graph and execute assigned job requests. Unlike many other remote control implementations, this design eliminates direct agent-to-agent communication, which would otherwise require exposing an internet-accessible server on the lab's desktop – often considered dangerous by IT staff.

A.2.10 Derived information stepping

Supplementary Figure S17 illustrates the technical implementation in the knowledge graph during the three stages of goal-driven agent dynamics. Supplementary Figure S17(a) illustrates the initialisation of the optimisation campaign upon the goal request by the scientist. It starts with ROG Agent parsing the resources and restrictions set for the optimisation. A goal set is formulated to include all objectives. To reach the target value of each goal, an optimisation plan is employed which is composed of a sequence of agents' actions, *i.e.* steps in an experimental workflow. A `Goal Iteration Derivation` instance is created per laboratory and requested for execution by ROGI Agent.

Supplementary Figure S17(b) presents the information flow between the agents within one iteration of pursuing the goal set. It starts with ROGI Agent parsing its inputs to formulate a `DesignOfExperiment` instance and creating tasks for corresponding agents listed in the optimisation plan, involving `DoE Derivation`, `Schedule Derivation`, and `PostProcessing Derivation`. The DoE Agent takes in the metadata for the DoE and proposes a new experiment. It is then scheduled by the Vapourtec Schedule Agent to the relevant hardware contained in the specified laboratory via creating tasks for the set of agents managing the hardware, *i.e.* `Vapourtec Derivation` for Vapourtec Agent and `HPLC Derivation` for HPLC Agent. These conditions are then populated to configure and actuate the hardware in the physical world. Once the HPLC report is generated, the HPLCPostPro Agent post-processes the chromatogram peaks to compute the objectives. These values are then attached to the output of `Goal Iteration Derivation` as the latest results.

Supplementary Figure S17(c) sketches the process of goal evaluation when determining the next steps after each iteration. The latest results are compared with the research goals by the ROG Agent to decide if the iteration should be progressed into the next round. If the goals are not met and resources are still available, the executed reaction experiment in this iteration will be attached as input to the `Goal Iteration Derivation`. ROG Agent will then issue a new request to the ROGI Agent for another iteration and update the allowance accordingly. This process repeats until either all goals are met or all resources are consumed.

The evolution of the derivation subgraph when performing a DMTA cycle in one laboratory is exemplified in Supplementary Fig. S18.

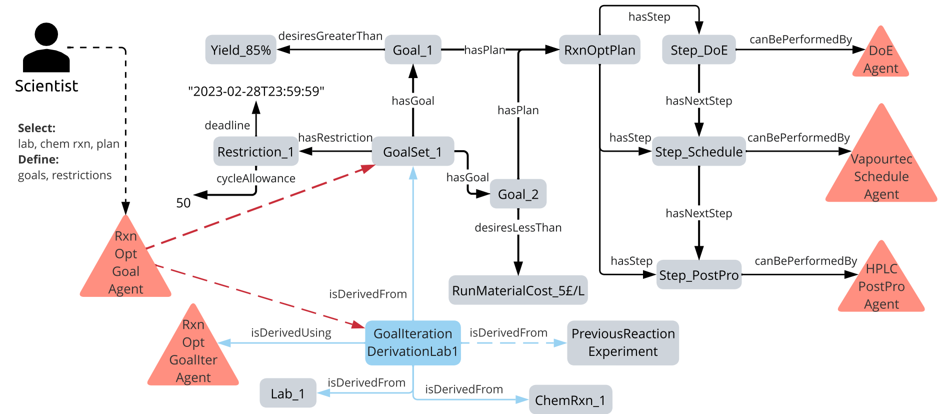

(a) Initialisation of goal iteration based on goal request from scientists.

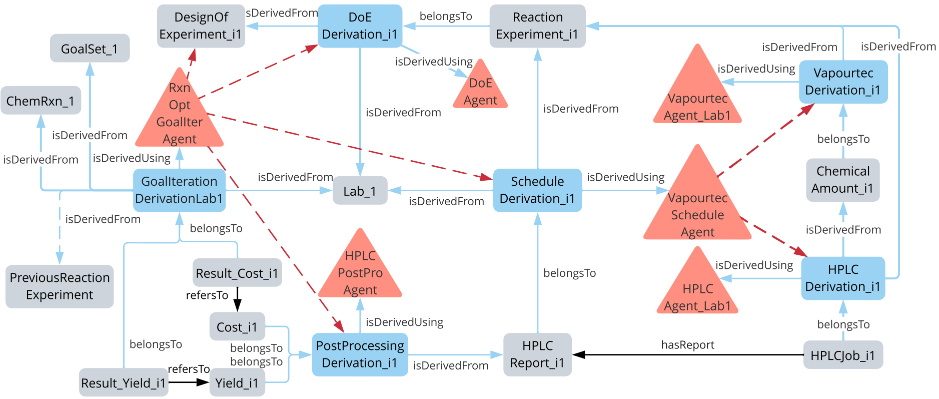

(b) Experiment workflow in one iteration of progressing towards the goal.

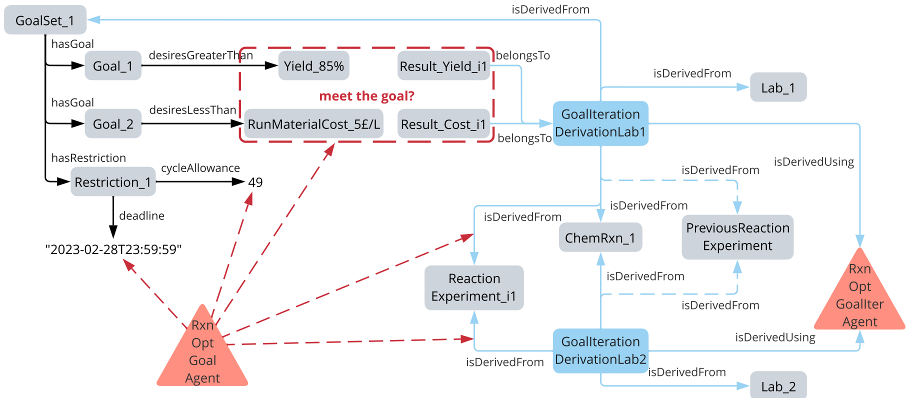

(c) Evaluation procedure when determining whether to progress to the next iteration.

Supplementary Figure S17: *Instantiation, iteration, and evaluation of the goal request from the scientist. Tasks in the workflow are denoted as "derivation". The red dashed lines refer to instantiation by the respective agent. The links to previous experiment results only exist should historical data be available.*

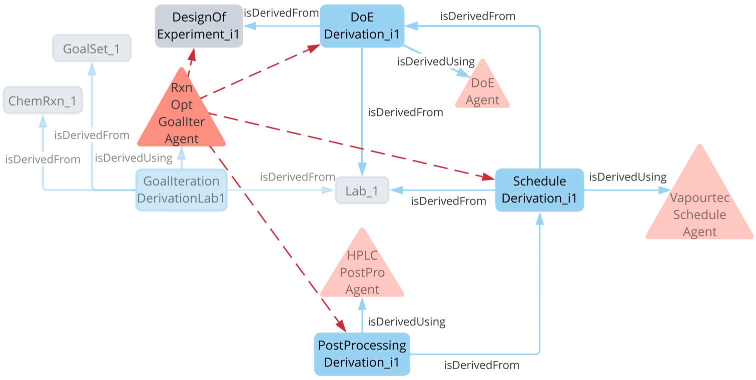

(a) Step 1: Initial workflow requested for the DMTA cycle.

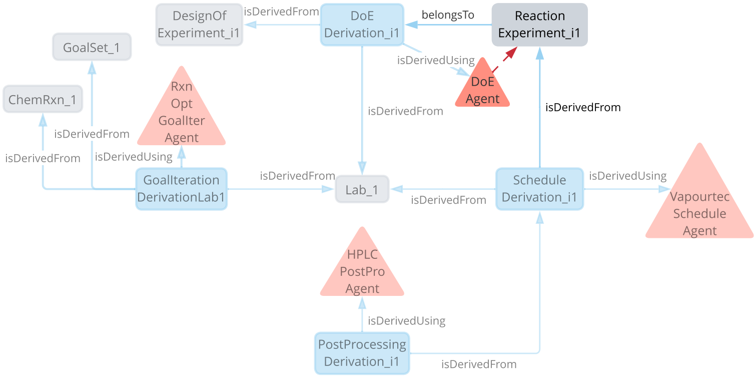

(b) Step 2: New experiment conditions suggested by the DoE Agent.

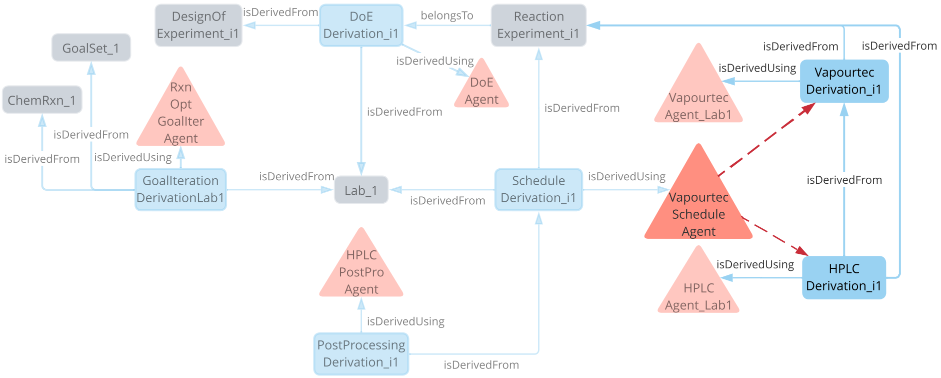

(c) Step 3: Experimentation and characterisation scheduled in the specified laboratory.

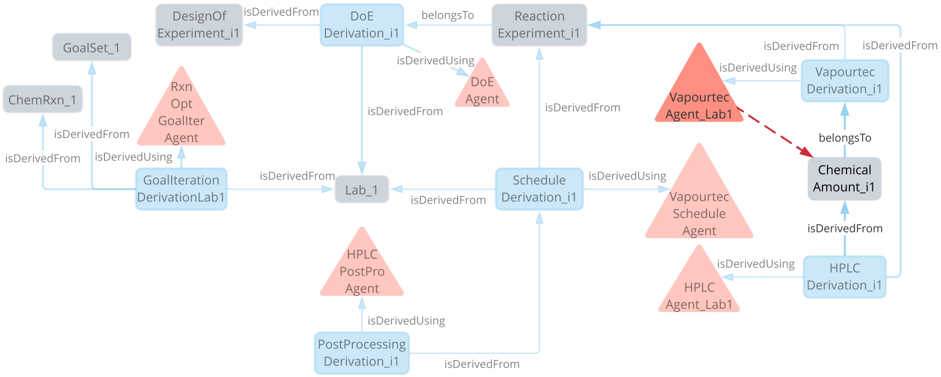

(d) Step 4: Reaction completed and sample injected into HPLC for analysis.

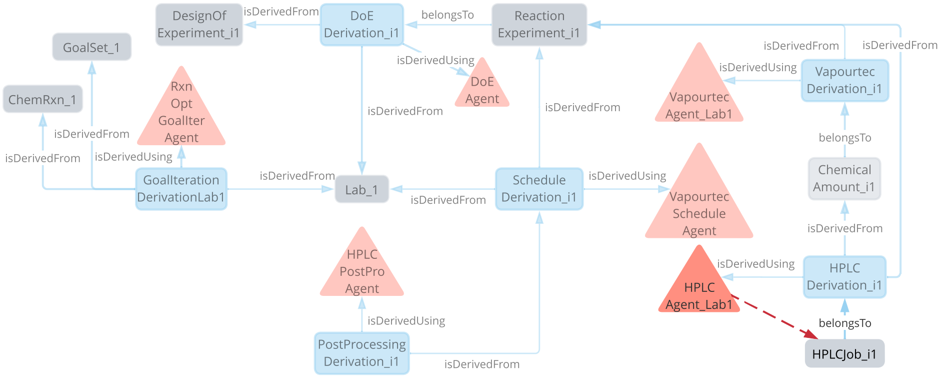

(e) Step 5: HPLC analysis completed with the raw report generated.

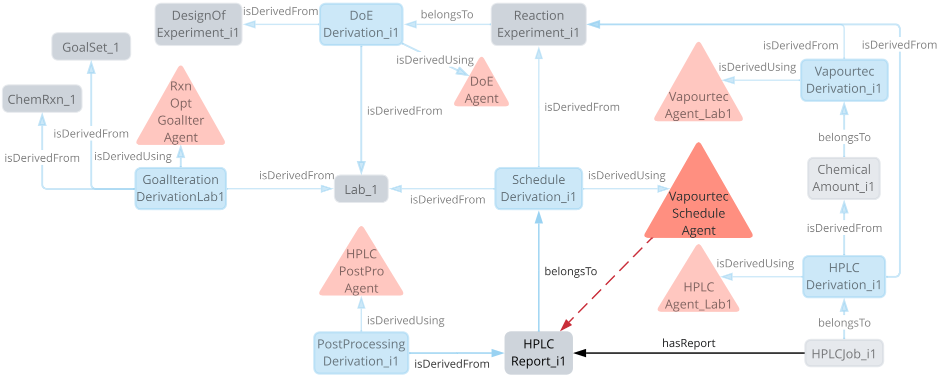

(f) Step 6: HPLC report delegated by the scheduler for post-processing.

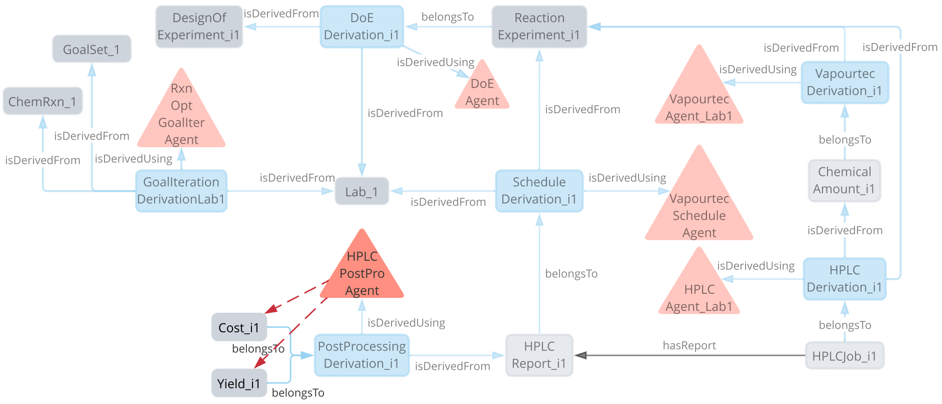

(g) Step 7: Performance indicators computed for the reaction experiment.

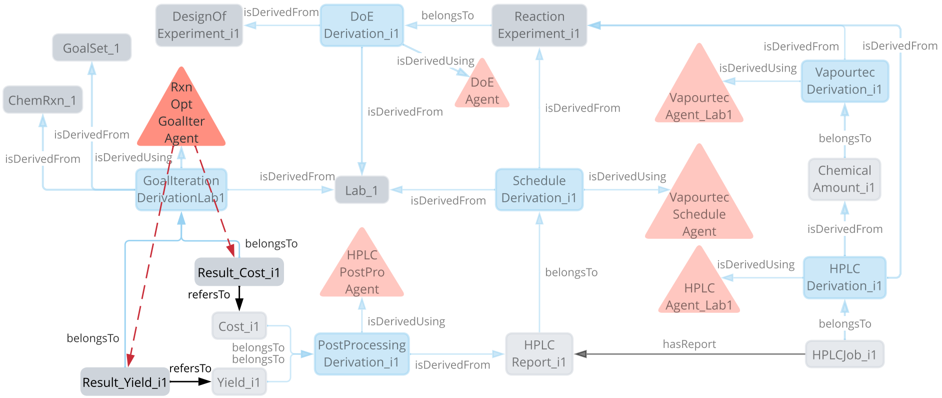

(h) Step 8: Objectives of interest added as iteration results and returned to decide the next step.

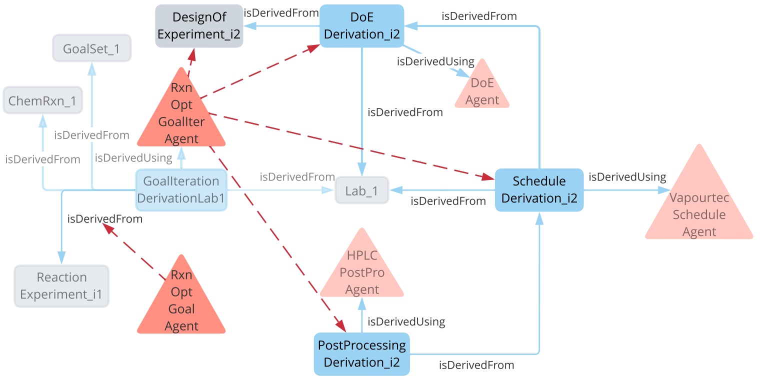

(i) Step 9: Goal iteration entered the next round with the previous experiment as historical data.

Supplementary Figure S18: *Stepping of the derived information generated in the knowledge graph during the initial goal iteration, assuming no prior data. The red dashed lines refer to instantiation by the respective agent. For simplicity, only instances that are necessary to connect the information chain are presented. The loop continues until the research goals are met or the resources are used up. Please refer to Supplementary Data for complete provenance records.*

A.3 Experimental

This section provides a detailed account of the experimental results. We begin by presenting the HPLC calibration data and the cost of chemicals used to calculate the yield and cost objectives. Next, we report the results obtained under control conditions, and finally present the results obtained during the self-optimisation campaign.

A.3.1 HPLC calibration for benzylideneactone

The concentration of actual product in Supplementary Eq. S1, *i.e.* Actual c_{product}, is calculated using the concentration of the internal standard (IS) as follows:

$$\text{Actual } c_{\text{product}} = \frac{1}{rf} \times \text{Actual } c_{\text{IS}} \times \frac{\text{Area}_{\text{product}}}{\text{Area}_{\text{IS}}}, \tag{S6}$$

where rf (response factor) is the slope obtained from a linear calibration curve of the form $y = rf \times x$ with zero intercept:

$$\frac{\text{Area}_{\text{product}}}{\text{Area}_{\text{IS}}} = rf \times \frac{c_{\text{product}}}{c_{\text{IS}}} \tag{S7}$$

Details regarding the HPLC calibration in each lab are given below. Supplementary Table S2 and Supplementary Fig. S19(a) refer to the Cambridge lab where biphenyl is adopted as IS. Supplementary Table S3 and Supplementary Fig. S19(b) refer to the Singapore lab where naphthalene is adopted as IS.

Supplementary Table S2: *HPLC calibration data for the analyte benzylideneacetone (4) with biphenyl as the internal standard in the Cambridge lab.*

[4] (M)	[IS] (M)	[4]/[IS]	Peak Area 4	Peak Area IS	Peak Area Ratio 4/IS
0.289	0.018	16.13	20397712	3803050	5.36
0.208	0.019	10.76	15315627	4065088	3.77
0.155	0.018	8.51	12198929	3996727	3.05
0.075	0.019	3.95	6062851	3922075	1.55

Supplementary Table S3: *HPLC calibration data for the analyte benzylideneacetone (4) with naphthalene as the internal standard in the Singapore lab.*

[4] (M)	[IS] (M)	[4]/[IS]	Peak Area 4	Peak Area IS	Peak Area Ratio 4/IS
0.503	0.05	10.05	5317	1604	3.31
0.404	0.05	8.08	4901	1741	2.82
0.308	0.05	6.16	3628	1783	2.03
0.204	0.05	4.08	2628	1849	1.42
0.096	0.05	1.92	1295	1922	0.67

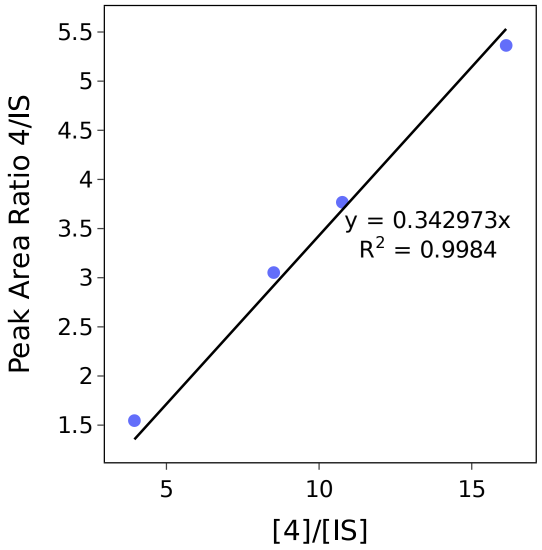

(a) Cambridge lab with biphenyl as the internal standard.

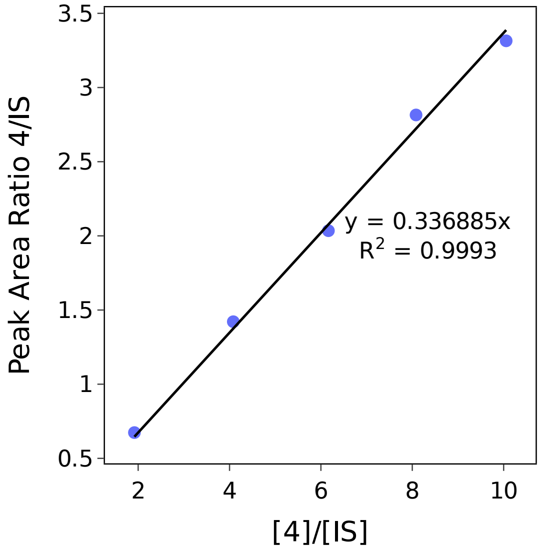

(b) Singapore lab with naphthalene as the internal standard.

Supplementary Figure S19: *HPLC calibration curve for the analyte benzylideneacetone (**4**) in the two labs.*

A.3.2 Cost of chemicals

Supplementary Table S4 lists the costs of reactants, catalyst, and solvents used to calculated the cost objective with Supplementary Eq. S3. These costs are obtained on the Cambridge side, while all chemicals used on the Singapore side are purchased from Sigma-Aldrich.

Supplementary Table S4: *Cost for chemicals used in the objective calculation.*

Chemical	Percentage Purity	Quantity	Cost (£ L^{-1})
Benzaldehyde	*ReagentPlus*®, ≥99%	£83.60 / 2.5 L[a]	33.44
Acetone	Laboratory Reagent, ≥99.5%	£28.45 / 1 L[b]	28.45
NaOH	≥97.0%	£39.90 / 500 g[c]	169.97[d]
Acetonitrile	HPLC Grade, ≥99.9%	£127.00 / 1 L[e]	127
Ethanol	≥99.8%	£327.5 / 2.5 L[f]	131

All below links were accessed on 21 Mar 2023.

[a] https://www.sigmaaldrich.com/GB/en/product/sial/b1334.

[b] https://www.fishersci.co.uk/shop/products/acetone-laboratory-reagent-99-5-honeywell-8/15691640#?keyword=67641.

[c] https://www.fishersci.co.uk/shop/products/sodium-hydroxide-white-pellets-fisher-bioreagents/10192863#?keyword=1310732

[d] Calculated using density 2.13 kg L^{-1} from https://pubchem.ncbi.nlm.nih.gov/compound/14798#section=Density&fullscreen=true.

[e] https://www.fishersci.co.uk/shop/products/acetonitrile-hplc-fisher-chemical-8/10754361?searchHijack=true&searchTerm=A%2F0626%2F17&searchType=RAPID&matchedCatNo=A%2F0626%2F17.

[f] https://uk.vwr.com/store/product/733157/ethanol-absolute-99-8-analar-normapur-acs-reag-ph-eur-analytical-reagent.

A.3.3 Reproducibility across laboratories

Supplementary Table S5 presents the two control conditions from both laboratories to ensure they produce consistent results.

Supplementary Table S5: *Control reactions and reproducibility for the lab in Cambridge and Singapore.*

Equiv. 2	Equiv. 3	Res. Time (min)	Temperature (°C)	Yield (%)	Lab
22.5	0.12	10	50	70.94	Cambridge
				71.44	
				71.35	
				70.62	Singapore
				72.61	
				71.43	
14.92	0.16	8.3	69	59.92	Cambridge
				60.43	Singapore

A.3.4 Self-optimisation campaign

Supplementary Table S6 lists the boundaries of the continuous variables involved in the self-optimisation campaign. These ranges cover most of the design space explored by Jeraal et al. [5] with both molar equivalents adjusted to ensure reliable pump flow rates in the Cambridge lab. Experiments were suggested by the TSEMO algorithm packaged in `summit` package [17] (0.8.9) with input parameters: `n_spectral_points=4000`, `n_retries=10`, `generations=1000`, and `pop_size=100`. All other parameters remained as their default values.

Supplementary Table S6: *Lower and upper limits used for the continuous variables in the self-optimisation campaign.*

Limits	Equiv. 2	Equiv. 3	Res. Time (min)	Temperature (°C)
Lower	5	0.05	5	30
Upper	40	0.2	15	70

Supplementary Figure S20 presents the hypervolume trajectory computed as a measure for the optimal trade-offs between cost-yield objectives, where a larger hypervolume corresponds to a more optimal Pareto front. Notably, the hypervolume did not show significant improvement after 24 iterations, which is approximately 12 hours since starting the campaign. Therefore, we consider the optimisation campaign has reached the optimal solution for this reaction.

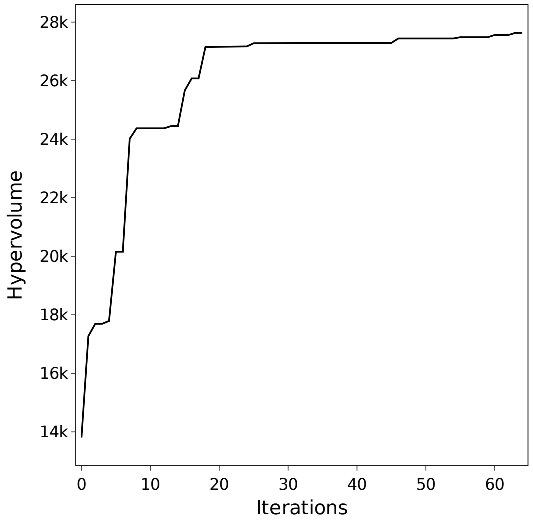

Supplementary Figure S20: *Hypervolume trajectory of the optimisation campaign with a reference point of 0% for yield and 500 £ L^{-1} for cost.*

Supplementary Table S7 lists all experiments executed during the self-optimisation campaign. For the complete knowledge graph please see Supplementary Data. An interactive version of the design of experiments animated over the timestamps is provided in Supplementary Movie 3. Notably, the namespaces adopted in the actual optimisation can be different from the example instantiations presented in previous sections of this Supplementary Information.

Supplementary Table S7: *Chronological summary of experiments recorded in the knowledge graph during the optimisation campaign, including autosampler sites for chemicals sourced in runs conducted at the Cambridge lab. Data is provided as a Source Data file.*

Reaction Partial IRI	Equiv. 2	Equiv. 3	Res. Time (min)	Temperature (°C)	Yield (%)	Cost (£ L^{-1})	Equiv. 1 Site	Equiv. 2 Site	Equiv. 3 Site	Lab
2a39187f-d2e3-4d89-819b-a980f83f9c5a*	11.08	0.06	13.78	66	50.24	225.19	2	14	15	Cambridge
412135fc-a3ca-4336-a50b-42a7498d58f9**	22.5	0.12	10	50	70.7	330.58				Singapore
2cf1175d-d7b9-4d5a-9315-8934fcf49b68	22.5	0.12	10	50	73.18	330.58				Singapore
473233f1-5ad7-4f1e-ad21-91e567bbf826	7.3	0.12	5.38	69	47.42	242.66				Singapore
b7f43e93-678f-4e06-ba4c-02a22b91b759	22.58	0.18	11.6	55	73.93	370.38				Singapore
0c015baa-942e-4075-aaef-6bba8627a141	30.35	0.12	12.91	60	93	375.99	1	14	21	Cambridge
9b02806a-006f-4b4c-9923-446117c34ef8	36.4	0.1	13.87	42	67.66	397.87				Singapore
743b9664-d886-4dda-82d1-09ae2df4a6f4	17.65	0.17	5.29	47	82.08	335.31				Singapore
3e87773f-5960-4c83-8139-070c296cc047	5.06	0.06	13.73	55	65.28	190.36	1	14	21	Cambridge
b07ebae5-2b76-4c1e-8075-a773db5f8ae2	14.21	0.08	10.73	53	60.61	256.4				Singapore
60f02bf0-97b9-430b-acde-451521776670	8.5	0.15	15	45	56.69	269.27				Singapore
a85a4029-3692-4461-8f92-9891d7c171e0	38.43	0.08	5.56	34	41.22	396.5	1	8	19	Cambridge
fa86bdfa-b900-40e5-abbb-16e1a96d8d03	7.97	0.05	10.17	49	0	200.64				Singapore
23d7f9c3-0f0a-45d6-813f-35cd2a60e722	15.86	0.14	9.95	69	68.22	305.28	1	8	19	Cambridge
b0dda4a0-0469-4120-abe6-a41c6ea601db	35.7	0.18	13.12	63	52.76	446.27				Singapore
e957ae0a-f48c-4cc1-9ceb-c2eacc219932	11.57	0.05	5	50	71.01	221.47				Singapore
61ebe163-2268-4f6c-8539-9d05372f6dec	22.78	0.06	13.07	60	89.53	292.86	1	8	19	Cambridge
cb5fe529-8f7b-4d39-8232-488ad06b71a6	7.83	0.16	5.42	31	38.71	271.95				Singapore
3c72a62a-16dd-46d5-a60e-e44fcab37485	8.62	0.05	8.11	62	52.56	204.4				Singapore
44d86f92-6ece-42c6-b468-9b394e868e8c	12.81	0.06	7.61	66	89.6	235.19	1	8	19	Cambridge
3ad34730-dd25-4894-9ad8-ecad05591cde	5	0.07	9.2	62	29.43	196.57				Singapore
4059a808-1401-4514-ab3e-048b2295e227	5	0.1	13.89	33	61.14	216.24	1	8	19	Cambridge
1245c932-7945-4cad-959b-633c407d1927	9.1	0.09	13.86	56	*3558.59*†	233.4				Singapore
bcd0150a-0e1c-4275-bbfb-3329272a8dee	8.41	0.2	10.3	69	66.38	301.53	1	8	19	Cambridge
c04c0ffb-9f2b-4e8a-ac17-07379ad776ef	6.07	0.05	7.1	45	22.15	189.65	1	8	19	Cambridge
13e12d18-8b6a-4e3b-ad07-de3bbff82370	5	0.05	12.74	33	17.4	183.46	1	8	19	Cambridge
205fedf7-8b45-4aa3-a71f-28b1d940062c	5.82	0.1	14.2	69	59.59	220.98	1	8	19	Cambridge
5cabcc77-f225-4bb8-a302-aad5b55e112c	35.89	0.05	15	55	41.37	362.14	1	8	19	Cambridge
1a5ec07d-5081-403a-ad70-1e8b118fbb23	15.18	0.05	8.65	68	33.95	242.35	2	12	18	Cambridge
48a7ca2f-dd1c-48fb-89a8-3a99e74162a1	5	0.07	5	34	1.19	196.57	2	12	18	Cambridge
90db1c6a-b000-4d4b-98d2-a2008587f196	19.43	0.07	13.18	64	87.39	280.04	6	12	18	Cambridge
e766d2c7-e15a-4fc3-8bf9-0f04ac3bad04	20.26	0.05	10.5	69	71.26	271.73	6	12	18	Cambridge
c765eb8c-c677-48ed-b631-9c727d22af33	16.24	0.07	9.11	30	34.14	261.59	6	12	18	Cambridge
0d6d3049-4582-4f41-8068-6b974845d027	7.26	0.06	5	68	44.04	203.09				Singapore

Continued

Supplementary Table S7: *(Continued)*

Reaction Partial IRI	Equiv. 2	Equiv. 3	Res. Time (min)	Temperature (°C)	Yield (%)	Cost (£ L^{-1})	Equiv. 1 Site	Equiv. 2 Site	Equiv. 3 Site	Lab
0c805777-0eac-4f77-b29b-a01022686b30	27.29	0.05	9.92	62	34.85	312.4	6	14	16	Cambridge
3d0fb333-f192-4763-ae4b-500711630692	5	0.06	8.04	69	61.64	190.02	6	14	16	Cambridge
8c0d6a77-c601-45eb-840a-4a1065729ac5	17.99	0.1	14.64	61	71.67	291.38	6	14	16	Cambridge
92151378-b70c-4907-99ca-2c0ccf839831	18.19	0.05	12.25	61	76.88	259.76	6	14	16	Cambridge
60953e65-1fe7-4666-bfbf-d3d5f5c9ba93	13.41	0.12	11.8	61	68	278	6	14	16	Cambridge
a07840d1-000d-457d-b198-67355065a4fe	19.23	0.05	13.5	63	*0*††	265.77	6	14	16	Cambridge
6f6514cb-299c-4686-a5fe-4755facdb2f1	5.08	0.2	12.61	49	59.59	282.26	6	14	16	Cambridge
b4a438bf-3fe0-44de-8dc3-f54a28ba0564	5	0.05	15	30	6.86	183.46	6	14	16	Cambridge
632f3d6c-e258-4051-99f7-5dc9a2ee3499	5	0.08	13.71	43	15.98	203.13	6	13	16	Cambridge
65a3db4b-9bdf-40dd-8f24-277eb599e5d7	5	0.05	6.15	30	2.75	183.46	7	13	16	Cambridge
3f28b2cf-dbb9-4426-99a4-a980f5bf2d9d	19.06	0.05	15	61	76.34	264.79	7	13	16	Cambridge
76aa4623-1747-4802-b9bf-f57dd79f047e	8.36	0.16	12.17	56	67.19	275.01	7	13	16	Cambridge
d42cd029-7be7-42bb-ae37-dc10e4f700a9	17.65	0.15	14.46	67	57.4	322.19	7	13	16	Cambridge
5ead68d1-9b19-4685-b602-f3329cc3b3bf	5	0.05	15	52	40.53	183.46	7	13	21	Cambridge
c2cb9ad0-42e2-4f4e-81ca-9058a6498812	23.93	0.09	15	62	82.83	319.18	5	12	17	Cambridge
c31c4dea-6edb-44bc-9e61-406b7db8e4ac	18.5	0.05	5	68	47.28	261.55	5	12	17	Cambridge
917da8be-6620-4cc7-b1df-95cb3d78ad49	22.29	0.1	12.37	58	72.64	316.25	5	12	17	Cambridge
52321782-ca81-4f7e-8296-c38ea2731e93	21.08	0.15	15	46	73.15	342.03	5	12	17	Cambridge
38323622-6232-40e0-b8ee-80b21466eb99	5.02	0.17	8.35	54	56.75	262.25	5	12	17	Cambridge
cd283f42-f882-43bc-ad3f-cf20896a0a0b	12.16	0.12	7.16	69	62.44	270.77	5	12	17	Cambridge
73f449aa-34ff-47d0-aa39-052d3cad34fb	5	0.11	14.69	69	47.93	222.8	5	12	17	Cambridge
c477e3d6-eea5-454c-b1f1-2c6ee528c05f	21.49	0.12	12.97	56	66.08	324.74	5	8	17	Cambridge
9c7bdf7c-b090-4896-afa6-fec99ab6e808	5.72	0.05	13.38	69	58.75	187.63	5	8	17	Cambridge
a85b964a-1e3b-4e45-8080-f64215698d01	17.83	0.07	14.54	48	32.41	270.79	5	8	17	Cambridge
e02256cd-dffe-4645-8080-dde8eb2dad19	5	0.09	15	66	48.93	209.69	5	13	17	Cambridge
50872203-0a25-42db-b8c5-aee440e7263c	5.63	0.07	12.9	57	27.28	200.22	5	13	17	Cambridge
8c8e54eb-4e73-4ddf-9a6d-abfd9ce8e299	22.52	0.12	9.85	65	70.59	330.7	4	13	18	Cambridge
b854e692-f6f4-494a-b6ac-f2103f4bda7f	8.23	0.05	15	69	69.21	202.14	4	13	18	Cambridge
98d72768-16d0-4a22-a89f-86c4ff9741d5	14.45	0.13	6.28	60	72.69	290.57	4	13	18	Cambridge
298eff02-90e0-42a1-95b7-e8d307547606	19.19	0.13	12	45	72.57	317.99	4	13	18	Cambridge
80a25214-4ea7-4a35-b255-f2042b75f16e	5	0.05	10.41	69	58	183.46	4	13	18	Cambridge
c253af7f-3bd6-43c5-9dcc-9d1c89dc5846	8.6	0.07	5	66	40.61	217.4	4	11	18	Cambridge

* *The complete IRI starts with* `https://www.theworldavatar.com/kg/lab_auto/derivation/ReactionExperiment_`

** *The complete IRI for all of the remaining rows starts with* `https://www.theworldavatar.com/kg/lab_auto/derivation/ReactionVariation_`

† *This yield is considered abnormal and therefore not utilised by the DoE algorithm, i.e. not included in the interactive animation. The correct yield should be 41.43%, which is still dominated.*

†† *Unfortunately, this wrong yield is utilised by the DoE algorithm. The correct yield should be 71.22%, which is still dominated.*

Supplementary References

[1] Morbach, J., Yang, A. & Marquardt, W. OntoCAPE - A Large-Scale Ontology for Chemical Process Engineering. *Eng. Appl. Artif. Intell.* **20**, 147–161 (2007).

[2] Farazi, F. *et al.* OntoKin: An Ontology for Chemical Kinetic Reaction Mechanisms. *J. Chem. Inf. Model.* **60**, 108–120 (2020).

[3] Bai, J. *et al.* Automated Calibration of a Poly(oxymethylene) Dimethyl Ether Oxidation Mechanism Using the Knowledge Graph Technology. *J. Chem. Inf. Model.* **61**, 1701–1717 (2021).

[4] Mosbach, S. *et al.* Multiscale Cross-Domain Thermochemical Knowledge-Graph. *J. Chem. Inf. Model.* **60**, 6155–6166 (2020).

[5] Jeraal, M. I., Sung, S. & Lapkin, A. A. A Machine Learning-Enabled Autonomous Flow Chemistry Platform for Process Optimization of Multiple Reaction Metrics. *Chem. Methods* **1**, 71–77 (2021).

[6] Garay-Ruiz, D. & Bo, C. Chemical Reaction Network Knowledge Graphs: The OntoRXN Ontology. *J. Cheminf.* **14**, 29 (2022).

[7] Krdzavac, N. *et al.* An Ontology and Semantic Web Service for Quantum Chemistry Calculations. *J. Chem. Inf. Model.* **59**, 3154–3165 (2019).

[8] Kearnes, S. M. *et al.* The Open Reaction Database. *J. Am. Chem. Soc.* **143**, 18820–18826 (2021).

[9] Pistoia Alliance. Unified Data Model (2020). URL https://github.com/PistoiaAlliance/UDM. Accessed 30 May 2023.

[10] Swain, M. C. & Cole, J. M. ChemDataExtractor: A Toolkit for Automated Extraction of Chemical Information from the Scientific Literature. *J. Chem. Inf. Model.* **56**, 1894–1904 (2016).

[11] Guo, J. *et al.* Automated Chemical Reaction Extraction from Scientific Literature. *J. Chem. Inf. Model.* (2021).

[12] EMBL-EBI. Name Reaction Ontology (2021). URL https://www.ebi.ac.uk/ols/ontologies/rxno. Accessed 30 May 2023.

[13] Rijgersberg, H., Van Assem, M. & Top, J. Ontology of Units of Measure and Related Concepts. *Semant. Web* **4**, 3–13 (2013).

[14] Soldatova, L. N. & King, R. D. An Ontology of Scientific Experiments. *J. R. Soc., Interface* **3**, 795–803 (2006).

[15] Blondet, G., Le Duigou, J., Boudaoud, N. & Eynard, B. An Ontology for Numerical Design of Experiments Processes. *Comput. Ind.* **94**, 26–40 (2018).

[16] Garud, S. S., Karimi, I. A. & Kraft, M. Design of Computer Experiments: A Review. *Comput. Chem. Eng.* **106**, 71–95 (2017).

[17] Felton, K. C., Rittig, J. G. & Lapkin, A. A. Summit: Benchmarking Machine Learning Methods for Reaction Optimisation. *Chemistry-Methods* **1**, 116–122 (2021).

[18] Daniele, L., Garcia-Castro, R., Lefrançois, M. & Poveda-Villalon, M. SAREF: The Smart Applications REFerence ontology (2020). URL `https://saref.etsi.org/core/v3.1.1/`. Accessed 21 Feb 2023.

[19] Bai, J. *et al.* A Derived Information Framework for a Dynamic Knowledge Graph and its Application to Smart Cities. *Future Gener. Comput. Syst.* **152**, 112–126 (2024).

[20] Rao, A. S. & Georgeff, M. P. Modeling Rational Agents within a BDI-Architecture. In *Proceedings of the Second International Conference on Principles of Knowledge Representation and Reasoning*, KR'91, 473–484 (Morgan Kaufmann Publishers Inc., San Francisco, CA, USA, 1991).

[21] Rao, A. S. & Georgeff, M. P. BDI Agents: From Theory to Practice. In *Proceedings of the First International Conference on Multi-Agent Systems*, ICMAS-95, 312–319 (1995).

[22] Bratman, M. *Intention, Plans, and Practical Reason* (Cambridge: Cambridge, MA: Harvard University Press, 1987).

[23] Bai, J. & Mosbach, S. `chemistry-and-robots`: Dataclasses and SPARQL Queries for Concepts related to Chemistry and Robots (2023). URL `https://pypi.org/project/chemistry-and-robots/`. Accessed 15 March 2023.

[24] Bai, J., Lee, K. F. & Mosbach, S. `pyderivationagent`: A Python Wrapper for Derivation Agents (2023). URL `https://pypi.org/project/pyderivationagent/`. Accessed 14 March 2023.

[25] Bradford, E., Schweidtmann, A. M. & Lapkin, A. Efficient Multiobjective Optimization Employing Gaussian Processes, Spectral Sampling and A Genetic Algorithm. *J. Glob. Optim.* **71**, 407–438 (2018).

[26] Nambiar, A. M. *et al.* Bayesian Optimization of Computer-Proposed Multistep Synthetic Routes on an Automated Robotic Flow Platform. *ACS Cent. Sci.* **8**, 825–836 (2022).

[27] Bordini, R. H. *et al.* A Survey of Programming Languages and Platforms for Multi-Agent Systems. *Informatica (Ljubljana)* **30**, 33–44 (2006).

[28] De Silva, L., Meneguzzi, F. R. & Logan, B. BDI Agent Architectures: A Survey. In *Proceedings of the 29th International Joint Conference on Artificial Intelligence (IJCAI), 2020, Japão.* (2020).
