# Supplementary material for: A dynamic knowledge graph approach to distributed self-driving laboratories
Source: Nat Commun. 2024 Jan 23;15:462. doi: 10.1038/s41467-023-44599-9 (PMC10805810; doi:10.1038/s41467-023-44599-9)
Supplement: Supplementary file 3 — Description of Additional Supplementary Files [file 41467_2023_44599_MOESM3_ESM.docx]

**Description of Additional Supplementary Files**

**Supplementary Data 1:** Complete records of the collected knowledge graph triples during the collaborative optimisation campaign, including both experimental data and their provenance. The digital twins of the laboratory hardware are anonymised. This file is accessible through common plain text editors, such as Notepad, Notepad++, and Sublime Text.

**Supplementary Movie 1:** An interactive animation of the progress of the Pareto front advancement (Fig. 6(a)) during the collaborative optimisation campaign.

**Supplementary Movie 2:** An interactive version of the 3D plot for the cost objective (Fig. 6(b)) during the collaborative optimisation campaign.

**Supplementary Movie 3:** An interactive version of the 3D plot for the yield objective (Fig. 6(c)) during the collaborative optimisation campaign.
